# Modulation properties of factors released by bone marrow stromal cells on activated microglia: an *in vitro* study

**DOI:** 10.1038/srep07514

**Published:** 2014-12-19

**Authors:** Dasa Cizkova, Stéphanie Devaux, Françoise Le Marrec-Croq, Julien Franck, Lucia Slovinska, Juraj Blasko, Jan Rosocha, Timea Spakova, Christophe Lefebvre, Isabelle Fournier, Michel Salzet

**Affiliations:** 1 Laboratoire PRISM: Protéomique, Réponse Inflammatoire, Spectrométrie de Masse, -U1192 INSERM, Bât SN3, 1^er^ étage, Université de Lille 1, F-59655 Villeneuve d'Ascq, France; 2 Institute of Neurobiology, Slovak Academy of Sciences, Center of Excellence for Brain Research, Soltesovej 4-6, 040 01 Kosice, Slovakia; 3 Associated Tissue Bank of the Pavol Jozef Safarik University, Faculty of Medicine, University Hospital of. Louis Pasteur in Kosice, Trieda SNP 1, 040 66 Kosice, Slovakia

## Abstract

In the present paper we develop a new non-cell based (cell-free) therapeutic approach applied to BV2 microglial cells and spinal cord derived primary microglia (PM) using conditioned media from rat bone marrow stromal cells (BMSCs-CM). First we collected conditioned media (CM) from either naive or injured rat spinal cord tissue (SCI-CM, inflammatory stimulation agent) and from rat bone marrow stromal cells (BMSCs-CM, therapeutic immunomodulation agent). They were both subsequently checked for the presence of chemokines and growth, neurotrophic and neural migration factors using proteomics analysis. The data clearly showed that rat BMSCs-CM contain *in vitro* growth factors, neural migration factors, osteogenic factors, differentiating factors and immunomodulators, whereas SCI-CM contain chemokines, chemoattractant factors and neurotrophic factors. Afterwards we determined whether the BMSCs-CM affect chemotactic activity, NO production, morphological and pro-apoptotic changes of either BV2 or PM cells once activated with SCI-CM. Our results confirm the anti-migratory and NO-inhibitory effects of BMSCs-CM on SCI-CM-activated microglia with higher impact on primary microglia. The cytotoxic effect of BMSCs-CM occurred only on SCI-CM-stimulated BV2 cells and PM, not on naive BV2 cells, nor on PM. Taken together, the molecular cocktail found in BMSCs-CM is favorable for immunomodulatory properties.

Bone Marrow Stromal Stem Cells (BMSCs) are a population of heterogeneous cells derived from the non-blood forming fraction of the bone marrow^1^. Under physiological conditions they provide stromal support for developing hematopoietic cells^2^ through the continuous release of erythropoietin (EPO) and granulocyte-colony stimulating factor (G-CSF). This continuous release of EPO and G-CSF provides the stromal support for developing hematopoietic cells. Although initially found in the bone marrow, adult stem cells capable of self-renewal and differentiation into various mesodermal cell lineages have been identified in many other organs and tissues including adipose tissue, umbilical cord, blood, skin, teeth, testes, gut, liver and ovarian epithelium^3^,^4^,^5^. Unlike other stem cells such as Keratinocyte Stem cells^6^,^7^,^8^, or pancreatic islet-derived stem cells^9^, BMSCs derived from the bone marrow produce low levels or none of class I and II Major Histocompatibility Complex (MHC) antigens and lack CD40, CD80 and CD86, co-stimulatory molecules required for activation of T cells. Furthermore, BMSCs are able to migrate to the site of inflammation and suppress the function of lymphocytes (T and B)^10^,^11^, natural killer cells^12^, dendritic cells^13^ and neutrophils^14^. The immunosuppressive properties of BMSCs give them a privileged role in ameliorating chronic inflammation-related neuronal damage in various central nervous system (CNS) disease models^15^,^16^,^17^,^18^. In the case of spinal cord lesion, after initial primary injury caused by direct mechanical insult, the spinal cord tissue progressively undergoes pathological changes that are associated with secondary damage affecting intact, neighboring tissue^19^,^20^. One of the key events of secondary processes is related to the development of acute inflammation characterized by fluid accumulation (edema) and the recruitment of immune cells (neutrophils, T-cells, macrophages and monocytes)^20^,^21^. The beneficial and detrimental effects of inflammation have been compared to glial scar, which is actively formed after spinal cord injury (SCI)^22^. Much evidence suggests that glial scar plays an important role in the immediate response to injury, corresponding to the acute phase^23^,^24^. Glial scar ensures sealing of the injury site, restoring homeostasis, preserving spared tissue and modulating immunity, however these roles become noxious for the recovery, neurogenesis and axonal growth in the later phases. Among the immune cells activated during inflammatory processes within the brain and spinal cord, microglia cells are one of the major effectors of immunity^25^. In response to injury, microglia proliferate and secrete cytotoxic nitric oxide (NO) and pro-inflammatory cytokines such as Tumor Necrosis Factor alpha (TNF-α) and Interleukin 1-beta (IL-1β)^26^,^27^ or neuroprotective molecules^28^. Thus, modulating reactive microglial cells via BMSCs-based therapy may limit chronic inflammation and tissue damage within the CNS^29^,^30^,^31^,^32^.

Moreover, BMSCs have been transplanted into rodent models of SCI by various research groups^33^,^34^,^35^,^36^. Despite considerable variation, a clear functional beneficial effects can be expected after transplantation of BMSCs into the injured spinal cord^37^,^38^,^39^,^40^. These effects include improvements in locomotion, sensorimotor function, promotion of axonal regeneration, and preservation of neural tissue. However, knowledge of the underlying mechanisms is essential to increase the overall outcomes. The question which arise is which molecules and receptors are involved in spinal cord repair? In this context, the content of BMSCs products was investigated at a proteomic level. Furthermore, in order to further understand the mechanism of BMSCs-mediated down-regulation of CNS inflammation, it is important to examine the influence of BMSCs on the activated microglia. Here we have developed a new non-cell based (cell-free) therapeutic approach applied to SCI-CM-stimulated BV2 cells and primary microglia (PM) isolated from rat spinal cord microglia. First we collected conditioned media from injured spinal cord tissue (Spinal Cord Injury Conditioned Media (SCI-CM): inflammatory stimulatory agent) and from rat BMSCs (BMSCs-CM: therapeutic immune-modulating agent) and checked their molecular pattern for the presence of inflammatory cytokines, chemokines and neurotrophic factors using proteomic analysis. Afterwards we evaluated the BMSCs-CM effects on chemotactic activity, and morphological and pathological changes of BV2 cells and PM following stimulation with SCI-CM. Our results confirmed the anti-migratory and cytotoxic effects of BMSCs-CM on BV2 cells and PM, and also Nitric Oxide (NO)-inhibitory effects on PM activated with SCI-CM, but not on control microglia. Furthermore, the molecules found in BMSCs-conditioned media via proteomics are favorable for the pathways involved in their immunomodulatory properties.

## Experimental Procedures

### Chemicals

All chemicals were of the highest purity obtainable. Water, formic acid (FA), trifluoroacetic acid (TFA), acetonitrile (ACN), and methanol (MeOH) were purchased from Biosolve B.V. (Valkenswaard, the Netherlands). Sequencing grade, modified porcine trypsin was purchased from Promega (Charbonnieres, France).

### Animals

The study was performed with the approval and according to the guidelines of the Institutional Animal Care and Use Committee of the Slovak Academy of Sciences and with the European Communities Council Directive (2010/63/EU) regarding the use of animals in Research, Slovak Law for Animal Protection No. 377/2012 and 436/2012.

### BMSCs culture and conditioned media collection

BMSCs were isolated from the bone marrow of three adult male Wistar rats (300 g), collected from the long bones (femur and tibia)^36^,^41^. The bone marrow was dissected into small pieces, gently homogenized, and filtered (70 μm) to remove bone fragments. Mononuclear cells (MNCs) were isolated by Ficoll density gradient centrifugation (1.077 g/mL; Sigma-Aldrich, Steinheim, Germany) at 400 g for 20 min. MNCs were collected from the interface, washed with alpha-MEM (LONZA, Walkersville Inc.), and centrifuged at 600 g for 10 min. The cell pellet was re-suspended in 1 mL of alpha-MEM, the pooled cells were counted, and their viability was assessed using the trypan blue dye exclusion method. MNCs were subsequently re-suspended in culture medium composed of alpha-Minimum essential media (MEM) supplemented with 10% of fetal calf serum (FCS)(GIBCO Laboratories, Grand Island, NY) and antibiotics (10,000 units/mL penicillin, 10,000 μg/mL streptomycin, and 25 μg/mL amphotericin B; Invitrogen, Carlsbad, CA), and plated at a density of 30.000 cells/cm^2^ in tissue culture flasks. The cells were incubated in a humidified atmosphere with 5% CO_2_ at 37°C. Non-adherent cells were removed after 4–5 days by medium change and the remaining cells were fed twice per week. When the cultures reached 80% of confluence, the BMSCs were passaged with 0.25% trypsin/0.53 mM Ethylene diamine tetra acetic (EDTA) (Invitrogen), centrifuged, and re-plated at a density of 5000 cells/cm^2^. The BMSCs were expanded 3 times to achieve the desired cell numbers. Cells at passages 3 cultured in Dulbecco's modification of Eagle's medium (DMEM) with low glucose and without fetal bovine serum were incubated in a humidified atmosphere with 5% CO_2_ at 37°C for 24 h and used for BMSCs conditioned media (BMSCs-CM) collection, using a similar protocol as in the previous study^42^.

### Characterization of rat bone marrow derived BMSCs

Before experimental use, the ability of BMSCs (from passage 3) to differentiate into adipocytes and osteoblasts was tested. To induce adipogenic differentiation, confluent adherent cells were cultured in alpha-MEM, supplemented with 10% of FCS, 1 μM dexamethasone (Sigma-Aldrich, Inc., USA), 500 μM 3-isobutyl-1-methylxanthine (Sigma-Aldrich, Inc., USA), 100 μM Indomethacin (Sigma-Aldrich, Inc., USA), and 10 μg/mL insulin (Sanofi-Aventis Deutschland GmbH), and the medium was replaced every 3 days. Oil Red O staining was used to identify adipocyte-differentiated BMSCs. To induce osteogenic differentiation, the alpha-MEM medium was supplemented with 10% of FBS, 0.1 μM dexamethasone, 10 mM beta-glycerophosphate (Sigma-Aldrich, Inc., USA), and 50 μM sodium L-ascorbate (Sigma-Aldrich, Inc., USA), and the medium was also replaced every 3 days. Cell differentiation into hydroxyapatite-producing osteoblasts was confirmed by Alizarin Red staining. BMSCs were maintained for 3 passages in alpha-MEM (LONZA, Walkersville Inc.), supplemented with 10% (v/v) FBS (LONZA, Walkersville Inc.) and 1% (v/v) antibiotic/antimycotic solution before being used for further analysis.

### Flow cytometry

The phenotypic properties of BMSCs were determined on the basis of the expression of CD90, CD29 and the absence of the pan-hematopoietic marker CD45 at passage 1 and 3. Briefly, BMSCs (0.2 × 10^6^ cells) were incubated with Phycoerythrin (PE)-conjugated antibodies or isotype-matched control immunoglobulin Gs (IgGs, 1 μg each) diluted in PBS containing 2% FCS, 2 mM EDTA, and 0.01% sodium azide (PFEA buffer) at 4°C for 45 minutes (BD Biosciences). For flow cytometry, the following antibodies were employed according to the supplier's recommendations: PE anti-mouse/rat CD29 (Clone: HMβ1-1, BioLegend); PE anti-rat CD45 (Clone: OX-1, BioLegend), and PE anti-rat CD90 (Clone: OX-7, BioLegend, all from San Diego, CA) and their isotype controls: PE Armenian Hamster IgG (CD29) and PE Mouse IgG1 (CD45, CD90) from Biolegend.

Samples were analyzed using a flow cytometer FACS Calibur (BD Biosciences) operated by CellQuest software and at least 20,000 events were collected per sample. Data were analyzed using WinMDI software (Version 2.8). Forward and side scatter profiles were obtained from the same samples.

### Conditioned media collection from spinal cord injury tissue

#### Spinal cord trauma

The spinal cord injury (SCI) was induced using the modified balloon compression technique in adult male Wistar rats (n = 4), weighing between 300 and 320 g, according to our previous study^43^. Manual bladder expression was required for 3 days after the injury. No antibiotic treatment was used. In the sham group/control (n = 4), a 2-French Fogarty catheter was inserted at the same level of spinal cord, but the balloon was not inflated and no lesion was made. All animals survived three days.

### Conditioned media collection from spinal cord injury tissue

Experimental SCI rats (n = 4) and control rats (n = 4) were sacrificed by isoflurane anesthesia followed by decapitation. The spinal cord was pressure flushed out by injecting sterile saline (10 mL) throughout the vertebral canal, along the caudo-rostral axis. Each spinal cord was macroscopically observed and the central lesion distinguished at Th7–Th10 level. Samples (approximately 1.0 cm) taken from the central lesion (were additionally chopped into 0.3 cm-thick sections/3per segment) and deposited into a 12-well culture plate, containing 2 mL of DMEM without FBS and without antibiotics. After 24 hours incubation in a humidified atmosphere with 5% CO_2_ at 37°C, 2 mL samples of SCI–CM were collected and centrifuged 30 min at 15.000 rpm at 4°C. The same procedure was performed for obtaining CM from control spinal cord tissue. A 50-μL aliquot from the 2 mL sample was used for trypsin digestion (24 h, 37°C). The portion was desalted using a solid-phase extraction procedure employing Millipore ZipTips. The solution was then dried again using the SpeedVac and re-suspended in water containing 5% of acetonitrile and 0.1% of formic acid before injection into nano Liquid Chromatography (LC).

### Microglia culture

Highly purified spinal cord derived primary microglia cultures (PM) were prepared using post natal day (P) 2–3 Wistar rats (Velaz, CZ) that were anesthetized on ice and afterwards sacrificed by decapitation^44^. The entire spinal cords were removed; meninges were dissected away; spinal cord tissue was minced with a sterile microsurgical scissors and digested with 1 mL trypsin trypsin/EDTA 1 × (Mediatech, Herndon, VA, USA) and 1 mL PBS for 15 min at 37°C. After centrifuging at 300 × g for 3 min, the cells were plated into 75 cm^2^ flasks which had been coated with poly-L-lysine. Mixed glial cells were cultured in DMEM containing 10% FBS at 37°C in 5% CO2 in air and 95% humidity. The culture medium, one-half of the volume was replaced with an equal volume of fresh growth medium after 6–7 days. After 12–14 days in vitro (DIV 13), the flasks were confluent with astrocytes and microglia. Flasks were agitated on shaker in laminar box (230 rpm, 37°C for 3 hours) supernatants were removed and centrifuged at 300 g for 10 min, plated into 75 cm^2^ flasks, after 30 min the supernatant containing mixed glial population (astrocytes + microglia) was removed and the adherent, highly enriched microglia was cultured with fresh media for 7–10 DIV. Adherent microglia enriched cultures were found to be 98.3 ± 0.52% microglia by staining with Iba1 antibody a marker for the microglia, while mixed glial population contained high percentage of astrocytes 95.8 ± 1.38%. The BV2 cells (Species: mouse, C57BL/6; Tissue: brain, microglial cells) were purchased from the IRCCS Azienda Ospedaliera, Universita San Martino (Italy)^45^.

### Experimental groups

The PM and BV2 cells were divided into five experimental groups: 1) Control group, cells were incubated in DMEM containing 2% fetal bovine serum (FBS) and 2) Conditioned media (CM) groups, where cells were incubated in DMEM containing 2% FBS (DMEM) and control spinal cord tissue SC-CM (DMEM:SC-CM (2:1), and 3) DMEM + BMSCs-CM (2:1), and 4) DMEM + SCI-CM (2:1), and 5) DMEM + SCI-CM + BMSCs-CM (1:1:1).

### Chemotaxis assays

The effects of conditioned media i) BMSCs-CM, ii) SC-CM (control spinal tissue/Th7–10 segment-conditioned medium), iii) SCI-CM (spinal cord injury tissue central lesion/Th7–10 segment-conditioned medium), and iv) BMSCs-CM + SCI-CM on microglial cell recruitment were determined using Boyden chambers (Cell Biolabs, CytoSelect™ 24-Well Cell Migration Assay, 5 μm)^46^. The BV2 cells were initially cultured in RPMI medium supplemented with 10% of FBS and 1% of penicillin/streptomycin (P/S), and split twice a week to obtain a sufficient number of BV2 cells. Before the experiment, the cells were replaced in a medium appropriate for the assay, which was DMEM with P/S (all reagents from Invitrogen). BV2 cells and PM at a concentration of 50000 per insert were plated into the upper chamber, while a different combination of CM (SC-CM, SCI-CM, SCI + BMSCs/CM, and BMSCs-CM) was filled into the lower one and then cultured for 3 hours. Each CM (1:2/CM:DMEM) concentration of total protein was measured using the Bradford protein assay (2.2 μg–2.8/10 μL/per each CM), and was centrifuged for 10 min at 1.500 rpm and sterilized through 0.2 μm filters prior to application. As positive control for microglial cell recruitment, ATP (10 μM) together with DMEM, the culture medium for microglia cells (negative control), was used. The migrating BV2 cells and PM were detected by Hoechst staining and the number was counted on dissected membranes transferred on glass slides and mounted with Vectashield mounting medium (*Vector Laboratories*, Inc. on LinkedIn). Three different counts under Nikon Eclipse Ti microscope with motorized stage were performed. Chemotaxis experiments were carried out in triplicate and their results are expressed as the mean of the microglial cell number ± SEM. *P < 0.01, *** P < 0.001, one-way ANOVA followed by Tukey-Kramer test (SigmaStat 3.11).

### Morphology and viability of Microglia

#### Morphology

To analyze the morphological changes of microglia after SCI-CM stimulation (DMEM:SCI-CM/2:1), SCI-CM co-cultured with BMSCs-CM (DMEM:SCI-CM:BMSCs-CM/1:1:1), or only with BMSCs-CM (DMEM:BMSCs-CM/2:1, the BV2cells and PM were plated in a concentration of 20000 cells per well of 24-well plates. Digital images of BV2 cells were taken at 3 h, 18 h, 24 h and 48 h and of PM at 24 h and 48 h in cultures stimulated with SCI-CM, SCI-CM+BMSCs/CM and BMSCs-CM (Nikon Ti). The percentage of BV2 ramified cells (spindle shaped or multipolar) over the total BV2 cells was calculated by using ImageJ software, at each time period within five sampling fields (500 × 500 μm) for each experimental group in triplicates. To confirm morphological characteristics of BV2 cells and PM after different CM treatment we have defined five following parameters: soma diameter, soma area, process diameter and length, and process length in relation to the soma diameter. Data were collected from measurements of 100 cells per CM treatment (Supplementary data).

### Immunohistochemistry

PM and BV2 cells after each CM treatment were fixed with 4% paraformaldehyde in phosphate buffered saline (PBS). After pre-incubation with 10% normal goat serum (NGS) in PBS for 60 min, the cells were washed 3 times with PBS and incubated with the primary antibody anti-Iba1 (a marker for microglia rabbit IgG, 1:1000; Wako Pure Chemical Industries, Osaka, Japan) (1:500) antibodies in PBS containing 2% NGS and 0.1% Triton X for 2 h. Cells were then washed 3 times and incubated with the secondary fluorescent antibody: goat anti-rabbit IgG conjugated with Texas Red (Alexa Flour 594). For nuclear staining, we used 4–6-diaminidino-2-phenylindol (DAPI) (1:200). Finally, cells were washed in 0.1 M PBS, mounted, and cover slipped with Vectashield mounting medium (Vector Laboratories, Inc.) and observed under a fluorescence microscope (Nikon Eclipse Ti, Japan) and confocal laser scanning microscope (Leica TCS SP5 AOBS, Leica Microsystems, Mannheim, Germany). The density analysis of Iba1 positive microglia was enrolled to evaluate possible morphological changes of activation form, based on the fact that hypertrophied/amoeboid microglia and its branched processes occupied larger micro-territory than resting type, in the identical sampling fields. Quantification for Iba1+ cells was performed at 40 × magnification and was analyzed by Image J software according to the previous protocol (Jones et al. 2002). In the monochrome 8-bit images we have determined the mean gray level number of black and white pixels (value 0–255, when 0 = white pixels, 255 = black pixels) within five identical sampling fields (500 × 500 μm) for each experimental group in triplicate. The threshold values were maintained at a constant level for all analyses. Data are represented as mean pixels ± SEM. *P < 0.1 **P < 0.01, *** P < 0.001, one-way ANOVA followed by Tukey-Kramer test.

### Viability and apoptosis

After 48 h incubation (each experimental group), the culture medium was aspirated, and the adherent BV2 cells and PM were harvested by trypsin-EDTA buffer at 37°C (5–7 min) followed by centrifugation. The cell pellets were washed twice in cold PBS (1.4 M NaCl, 27 mMKCl, 100 mM KH2PO4/K2HPO4, pH 7.2), suspended in 1 × binding buffer at 1 × 10^6^ cells/mL, and stained with Annexin V/Propidium Iodide labeling kit (Apoptosis Detection Kit I, BD Pharmingen, San Jose, CA) to determine dead and apoptotic cells. Labeling was done with 5 μL PI and 5 μL AV (PI, a standard probe used to distinguish viable cells from nonviable ones; AV, recombinant Annexin-V conjugated to green-fluorescent Alexa Fluor® 488 dye recognize the externalization of phosphatidylserine in apoptotic cells, BD Biosciences) at a concentration of 50 μg/mL. After standing for 15 min in the dark, the cells were transferred to cytospin slides by centrifugation of 200 μL sample/10 min 300 g, cover-slipped, mounted with Vectashield mounting medium with 1.5 μg/mL DAPI (Vector Laboratories). The percentage of necrotic AV−/PI+ and late apoptotic AV+/PI+ (BD Bioscience) cells to total DAPI stained nuclei were counted within five sampling fields (500 × 500 μm) for each experimental group in triplicates. The results are expressed as the mean % of ramified microglial cell number. Data are represented as mean ± SEM. *P < 0.1 **P < 0.01, *** P < 0.001, one-way ANOVA followed by Tukey-Kramer test.

### Griess assay for nitric oxide production

Nitric oxide production was assessed by using the Greiss Assay (Promega, Madison, WI, USA) following the manufacturer's protocol. Detection of nitrite was performed in 96-well plates, BV2 cells and PM (5 × 10^5^ cells/well) were incubated in DMEM containing 2% FBS alone or in combination with SC-CM, and BMSCs-CM, and SCI-CM, and SCI-CM + BMSCs-CM, ratio 2:1/DMEM:CM for 24 hours. NO was detected in the 50 μL of culture supernatant from each sample in triplicate and added with the same volume of Griess reagent (1% sulfanilamide/0.1% N-1- napthylethylenediaminedihydrochloride/2.5% phosphoric acid; all from Sigma-Aldrich, St. Louis, MO, USA). Absorbance was read at 530 nm (MRX II microplate reader, Dynex Technologies, VA, and USA) after 15 minute incubation. Nitrite concentration was calculated with reference to a standard curve of freshly prepared sodium nitrite (0 to 100 μM). All treatments were completed at least three times and data were expressed as mean μM concentration of NO_2_ ± SEM.

### Proteomic studies

Three different combinations were applied to the BMSCs-CM before analyzing them using on-line coupling of nanoLC with an ESI MS instrument (ESI-LTQ XL orbitrap, built in 2010) and each experiment was conducted in triplicate.

### Bottom-up analyses

- In the first set of analyses, 50 μL of the solution of BMSCs-CM was added to 25 μL of a solution of dithiotheitol (DTT) (50 mM) in ammonium bicarconate (NH_4_HCO_3_) buffer (50 mM) (pH = 8) and heated for 15 min at 55°C. After cooling, 25 μL of a solution of IAA (150 mM) in NH_4_HCO_3_ buffer (50 mM) was added and the mixture was incubated for 15 min at room temperature in the dark. 20 μL of a solution of trypsin (20 μg/mL) in NH_4_HCO_3_ (50 mM) was then added and the sample was incubated overnight at 37°C.
- In the second set of analyses, 900 μL of the solution of BMSCs-CM was filtered with Sep-Pak Shorty C18 (Waters Corporation, Milford MA, USA), dried under vacuum and then re-suspended in 50 μL of DTT (50 mM) in NH_4_HCO_3_ buffer (50 mM) (pH = 8), sonicated and heated for 30 min at 93°C. After cooling, 50 μL of a solution of IAA (150 mM) in NH_4_HCO_3_ buffer (50 mM) was added and the mixture was incubated for 20 min at room temperature in the dark. 50 μL of a solution of trypsin (20 μg/mL) in NH_4_HCO_3_ (50 mM) was then added and the sample was incubated overnight at 37°C. The digestion was stopped by adding 1 μL of trifluoro acetic acid (TFA), and the product was dried in a vacuum concentrator system.
- In the third set of analyses, 1 mL of the solution of BMSCs-CM was centrifuged at 12000 g for 10 min and the supernatant was collected and dried. 100 μL of Laemmli was used to re-suspend the pellet. The solution was incubated for 30 min at 93°C. The solution was then loaded into a 12% polyacrylamide gel, stacked at 70 V for 15 min and then separated at 120 V until the dye front reaches the other end of the gel. After migration, the gel was incubated in the gel fixative solution for 30 min and stained with Colloidal Coomassie brilliant blue overnight. The stain was removed by washing the gel four times with distilled deionized water. The gel was cut into eight pieces. The pieces were washed with 300 μL of distilled deionized water for 15 min, 300 μL of ACN for 15 min and 300 μL of 100 mM NH_4_HCO_3_ (pH8) for 15 min. A mix of 300 μL of NH_4_HCO_3_/ACN (1:1, v/v) was added for 15 min and 300 μL of ACN for 5 min. Band pieces were dried in a vacuum concentrator for 5 min. The reduction of cysteine residues was performed with 50 μL of 10 mM DTT in 100 mM NH_4_HCO_3_ (pH8) followed by incubation at 56°C for 1 hour. Alkylation of cysteine was obtained by addition of 50 μL of 50 mM IAA in 100 mM NH_4_HCO_3_ (pH8) followed by incubation at room temperature in the dark for 30 min. Band pieces were then washed once more with 300 μL of 100 mM NH_4_HCO_3_ (pH8) for 15 min, 300 μL of NH_4_HCO_3_/ACN (1:1, v/v) for 15 min and 300 μL of ACN for 5 min. Band pieces were dried in a vacuum concentrator for 5 min. Bands were digested by addition of trypsin (12.5 μg/mL) in 20 mM NH_4_HCO_3_ (pH8) (enough to cover pieces) followed by incubation at 37°C overnight. Peptides were then extracted on a shaking platform with 50 μL of 1% FA two times for 20 min and 150 μL of ACN for 10 min. The supernatant was transferred into the new tube and dried in a vacuum concentrator.

### NanoLC-MS & MS/MS

Samples from 3 sets of experiments were re-suspended in 20 μL of TFA 0.1%, then they were desalted on a C-18 Ziptip dried under vacuum and then re-suspended in AcN/0.1% FA, 8:2, v/v). The samples were separated by online reversed-phase chromatography using a Thermo Scientific Proxeon Easy-nLC system equipped with a Proxeon trap column (100 μm ID × 2 cm, Thermo Scientific) and C18 packed tip column (100 μm ID × 10 cm, NikkyoTechnos Co. Ltd). Elution was carried out using an increasing gradient of AcN (5% to 30% over 120 min) at a flow rate of 300 nL/min. A voltage of 1.6 kV was applied via the liquid junction of the nanospray source. The chromatography system was coupled to a Thermo Scientific LTQ-Orbitrap XL mass spectrometer programmed to acquire in data-dependent mode. The survey scans were acquired in the Orbitrap mass analyzer operated at 60,000 (FWHM) resolving power. A mass range of 300 to 2000 m/z and a target of 1E6 ions were used for the survey scans. Precursor ions observed with an intensity over 500 counts were selected “on the fly” for ion trap collision-induced dissociation (CID) fragmentation with an isolation window of 4 a.m.u. and a normalized collision energy of 35%. A target of 5000 ions and a maximum injection time of 200 ms were used for MS^2^ spectra. The method was set to analyze the top 20 most intense ions from the survey scan and dynamic exclusion was enabled for 20 s.

### Data analyses

All MS/MS samples were analyzed using Sequest (Thermo Fisher Scientific, San Jose, CA, USA; version 1.3.0.339) and X! Tandem (The GPM, thegpm.org; version CYCLONE (2010.12.01.1)). Sequest was set up to search *Rattus norvegicus* Uniprot ref proteome 112011.fasta assuming the digestion enzyme trypsin. X! Tandem was set up to search a subset of the RAT database also assuming trypsin. Sequest and X! Tandem were searched with a fragment ion mass tolerance of 0.50 Da and a parent ion tolerance of 10 ppm. Carbamidomethylation of cysteine was specified in Sequest and X! Tandem as a fixed modification. Glu->pyro-Glu of the n-terminus, ammonia-loss of the n-terminus, gln->pyro-Glu of the n-terminus, amidation of the c-terminus, oxidation of methionine, acetylation of the n-terminus and phosphorylation of tyrosine were specified in X! Tandem as variable modifications. Oxidation of methionine, acetylation of the n-terminus and phosphorylation of tyrosine were specified in Sequest as variable modifications. Scaffold (version Scaffold_4.0.6.1, Proteome Software Inc., Portland, OR) was used to validate MS/MS-based peptide and protein identifications.

### Label free quantification

For the validation of protein identifications obtained from Sequest and X! Tandem, the protein identifications were accepted if they could be established at greater than 99% probability and contained at least 2 identified peptides (FDR 0.1%). Protein probabilities were assigned by the Protein Prophet algorithm^47^,^48^. Peptide identifications were accepted if they could be established at greater than 95% probability by the Peptide Prophet algorithm^49^ with Scaffold delta-mass correction. Proteins that contained similar peptides and could not be differentiated based on MS/MS analysis alone were grouped to satisfy the principles of parsimony. Normalization followed by quantification was done on top 3 total ion current (TIC) in addition to spectral counting.

## Results and Discussion

### Characterization of bone marrow stromal stem cells (BMSCs)

**Figure 1:**
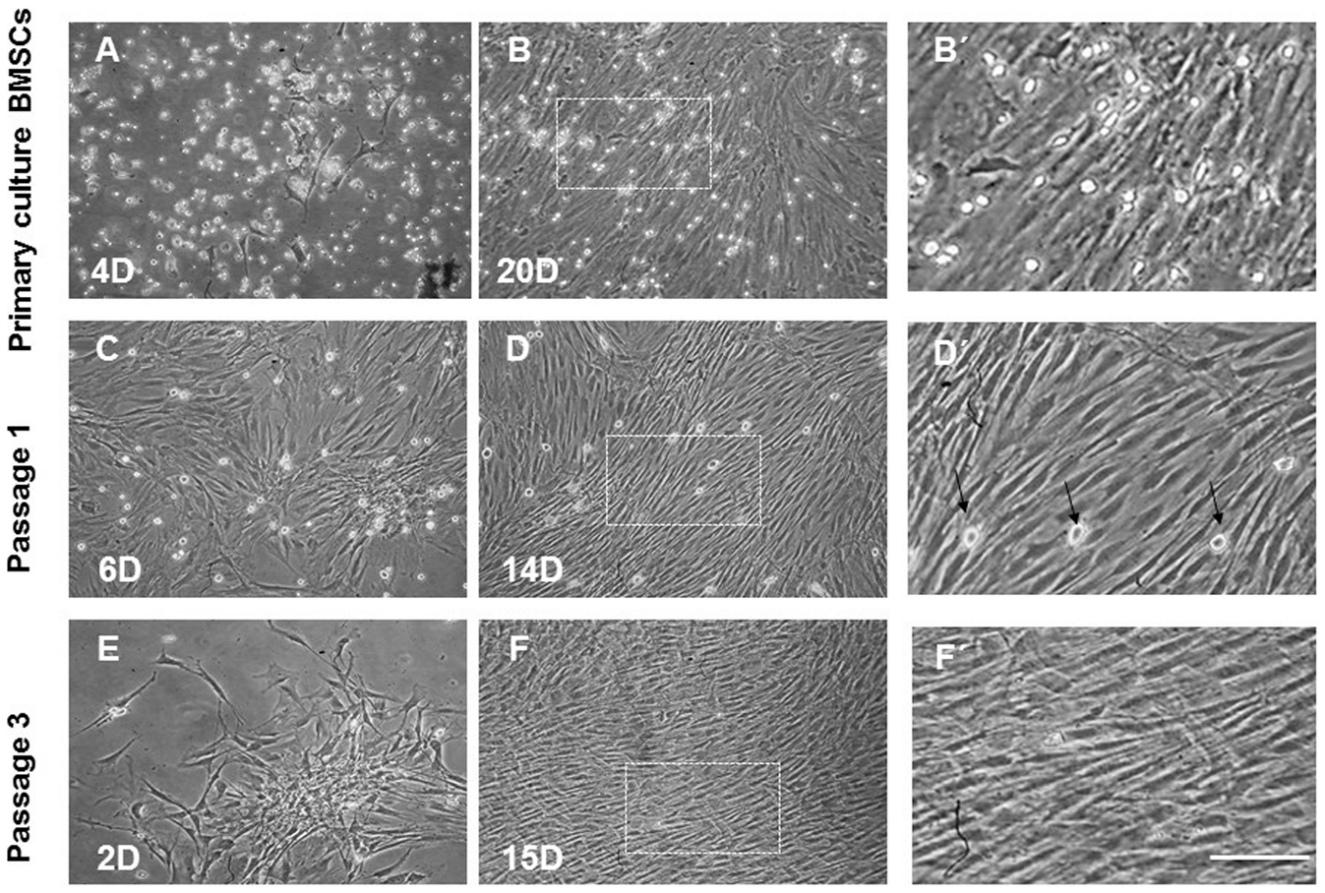
Representative Fields showing BMSCs morphologies in primary culture and after passaging. BMSCs showed diverse morphologies including ovoid, bipolar and large, flattened cells enriched with a large number of small, round-like floating light cells in the primary culture (A, B, B′). In passage 1, most of the BMSCs exhibited large, flattened or fibroblast-like morphology with sporadically occurring round, bright cells (C, D, D′, arrows) that were completely abolished after passage 3 (E, F, F′). Images B′, D′, F′ correspond to boxed areas from B, D, F. Scale bars A–F = 200 μm; B′, D′, F′ = 50 μm.

**Figure 2:**
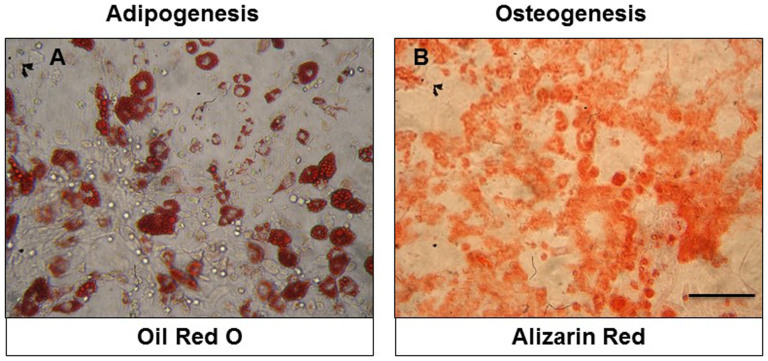
Micrographs documenting multipotent characteristics of BMSCs, differentiated into adipocytes (A) and osteocytes (B). Scale bars A, B = 100 μm.

BMSCs isolated from rat bone marrow were expanded in primary culture and passaged three times. Cultured cells at initial phases of growth contained attached spindle-shaped cells forming colonies and small, bright, round, floating cells (Figures 1A–D), reaching confluence approximately at day 14 (Figures 1B, D, F). The initial high number of bright floating cells in the primary culture of BMSCs (Figures 1A–B′) significantly decreased at passage 1 (Figure 1C–D′) or was completely abolished at passage 3 (Figures 1E–F′). To examine the multipotent differentiation potential, we showed that BMSCs generated Oil red-O positive fat cells, while for the osteoblasts, we visualized them with Alizarin Red Solution (Figures 2A and 2B). BMSCs were characterized immunophenotypically and confirmed throughout passages 1–3 using a panel of hematopoietic and non-hematopoietic markers (Table 1).

### Immunophenotyping of BMSCs

**Figure 3:**
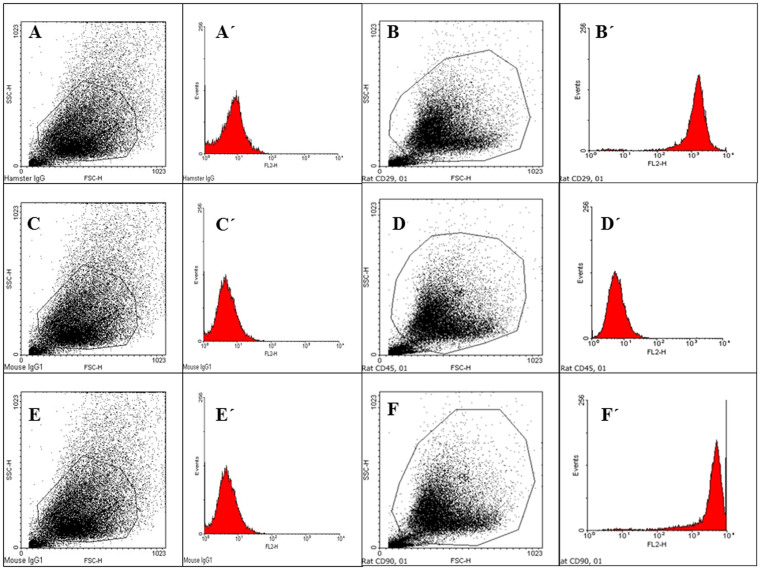
Representative flow cytometry analysis of cell surface markers (CD29, CD45, and CD90) expressed on BMSCs at passage 3.

BMSCs at passage 3 expressed CD29 (94%) and CD90 (96%) but not hematopoietic surface marker CD45 (Table 1, Figure 3) and maintained their typical phenotype throughout passages 4–5 (data not shown). As isotype controls, PE-conjugated Mouse IgG1 (C, C′, E, E′) was used for CD45 (D, D′) and CD90 (F, F′); PE-conjugated Armenian Hamster IgG (A, A′) was used for CD29 (B, B′).

### Proteomic studies: identification of BMSCs–CM content

**Figure 4:**
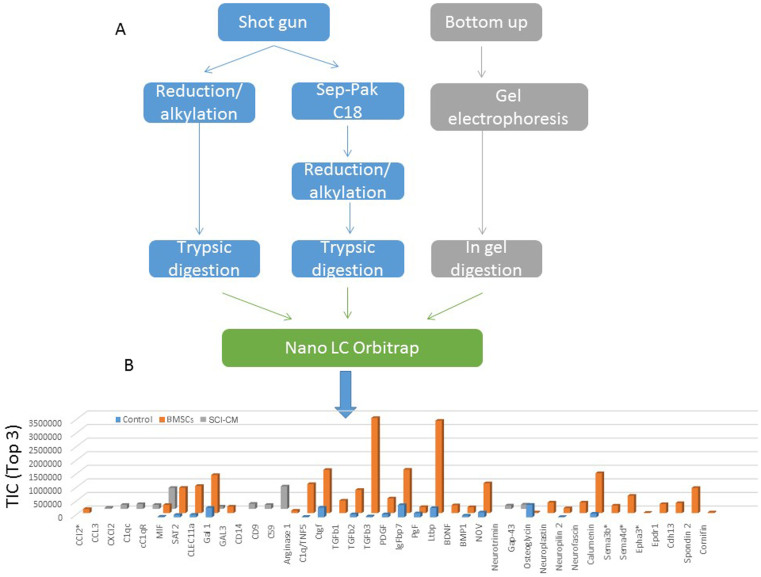
(A) Scheme of the methods used to analyze BMSCs Proteins identification was done on an orbitrap LTQ XL with a protein threshold of 99% (FDR 0.1%) and peptide threshold of 97% (FDR 0.1%) and contained at least 2 identified peptides as parameters (Supplementary data 1&2). (B) Label free quantification of immune modulators, neurotrophic and growth factors and apoptotic molecules identified in conditioned media obtained from BMSCs, spinal cord lesion (SCI) or control (non injured) using Scaffold_4.0.6.1. Label-free quantification was done on top3 of the total ion current (TIC). (BMP 1: Bone morphogenic protein 1, Cdh13: T-Cadherin, CLEC11a: C-type lectin domain family 11 member A CTGF: connective tissue growth factor, Epha 3: Ephrin A isoform 3, Epdr1: Ependymin related protein 1, FAM3C: family with sequence similarity 3, member C Gal 1: galectin 1; GAP-43: Growth associated protein 43, Ltbp: Latent TGF-beta binding protein, IgFbp 7: Insulin growth factor binding protein 7, MIF: macrophage inhibiting factor, PDGF: Platelet derived growth factor, PGF: placenta growth factor, spondin 2, SPARC, TIMP-1: Tissue inhibitor metalloproteinase 1 & 2).

BMSCs-CM collected after one day culture were centrifuged and the supernatant subjected to different procedures. These were combined (Figure 4A) and subjected to separation using a nano-LC coupled to an ESI-LTQ-Orbitrap XL instrument for LC MS &MS/MS analysis. We identified 658 proteins based on 99% probability and contained at least 2 identified peptides with a false discovery rate (FDR) of 0.1%. Each accession number, protein description, gene name and relative score associated with the selected proteins is reported in Table 2 (Supplementary data 1 & 2). Specific markers of BMSCs were identified *e.g.* osteogenic factors, like osteopotin, periostin, spondin 2 and osteoglycin, differentiating factors (SPARC, FAM3C, cornifin) as well as growth factors *i.e.* Placenta growth factor, Platelet derived growth factor, Insulin-like growth factor-binding protein 7, Transforming growth factor beta-1, 2 & 3, and Matricellular proteins of the CCN family (CYR61/CTGF/NOV). Moreover, proteins involved in immunomodulation (arginase 1, ST2, galectins, TIMP-1 and TIMP-2) and in chemotaxis (C-type lectin11a) were identified (supplementary data 1 & 2). The proteins identified in conditioned media obtained either from BMSCs, 3 days injured spinal cord (SCI) or non-injured spinal cord (Control) were compared after shot-gun analyses using scaffold proteome software 4.06. In addition a spectral counting and normalization based on total ion current was done in the three samples. The TOP3 in TIC was used for label-free quantification (Figure 4B). Data clearly reflected that BMSCs produce growth factors (CTGF, PDGF, PGF, TGFβ, IGF binding protein 7, BMP 1, C1q/TNF5), neural migration factors (NOV, neurosfascin, neuropilin 2, neuroplastin,) and immunomodulators (arginase 1, ST2, galectins, metalloproteinase inhibitors (supplementary data 1&2)) and chemoattractant factors (CLEC11a) (Table 2). These differences showed that BMSCs produce both factors involved in microglia chemoattraction (CCL2) and factors involved in inflammatory regulation (arginase 1, ST2, galectins), whereas SCI-CM contain chemokines (CCL3, CXCL2, MIF) and neurotrophic factors (GAP-43 and neurotrimin). According to Riffeld and collaborators^50^, BMSCs have been shown to secrete various growth factors, including brain-derived neurotrophic factor (BDNF), glial-derived neurotrophic factor (GDNF), vascular endothelial growth factor (VEGF), fibroblast growth factor 2 (FGF-2), nerve growth factor (NGF) and neurotrophin-3. Our proteomic data confirm and complete previous work. Thus, based on the secretion profile, BMSCs-CM. may contribute to neuroprotection in a direct manner by rescuing neural cells and switching the microglial cells/macrophages to a M2 polarization^33^.

### Modulation of stimulated microglia (BV2, PM) using BMSCs-CM

#### Attenuation of microglia recruitment

**Figure 5:**
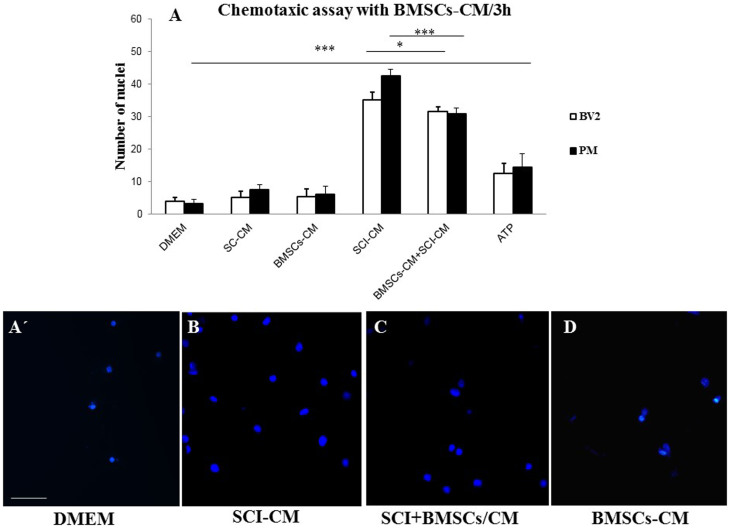
BMSCs-CM inhibition of BV2 cell and PM chemotaxis in the trans-well assay. Note that the high number of migrated BV2 cells and PM induced by SCI-CM (lesion site) (A, B) was attenuated when they were co-incubated with BMSCs conditioned media (SCI+BMSCs/CM BMSCs/CM) (A, C), as quantified by the number of Hoechst labeled cells. Incubation of BV2 cells and PM with BMSCs-CM or DMEM had low influence on their migration (A, D). Data are represented as mean ± SEM. *P < 0.01, *** P < 0.001, one-way ANOVA followed by Tukey-Kramer test. Scale bars A′ = 100 μm; B, C, D = 50 μm.

Proteins released from the central lesion segment of injured and control spinal cords were analyzed on microglial BV2 cells or PM using Boyden chambers in the presence or absence of BMSCs–CM. Activation of BV2 cells and PM was measured and quantified by counting the number of Hoechst labeled cells attached to the Boyden membrane. More than a 7-fold increase of attached BV2 cells (35.2 ± 2.2) and 8.5-fold increase of PM (42.5 ± 1.9) attached cells were obtained with the conditioned media from SCI (SCI-CM) compared to those from the control spinal cord SC-CM (5.2–7.5 ± 1.9), BMSCs-CM (5.5–6.2 ± 2.4) or DMEM (3.2–4.1 ± 1.1) (Figures 5A, A′, B–D). The simultaneous application of BMSCs-CM and SCI-CM (BMSCs-CM + SCI-CM), significantly diminished BV2 cells (31.6 ± 1.3,*P < 0.1) and PM microglial (30.8 ± 1.9, *** P < 0.001) mobility (Figures 5A, 5C) in comparison to SCI-CM alone (Figures 5A, 5B). Whereas, application of BMSCs-CM or DMEM had low influence on BV2 cells and PM migration (Figures 5A, 5D). These data confirmed the involvement of BMSCs in immune response modulation of microglial cells once activated.

### Stimulation of microglia: morphological changes and viability

**Figure 6:**
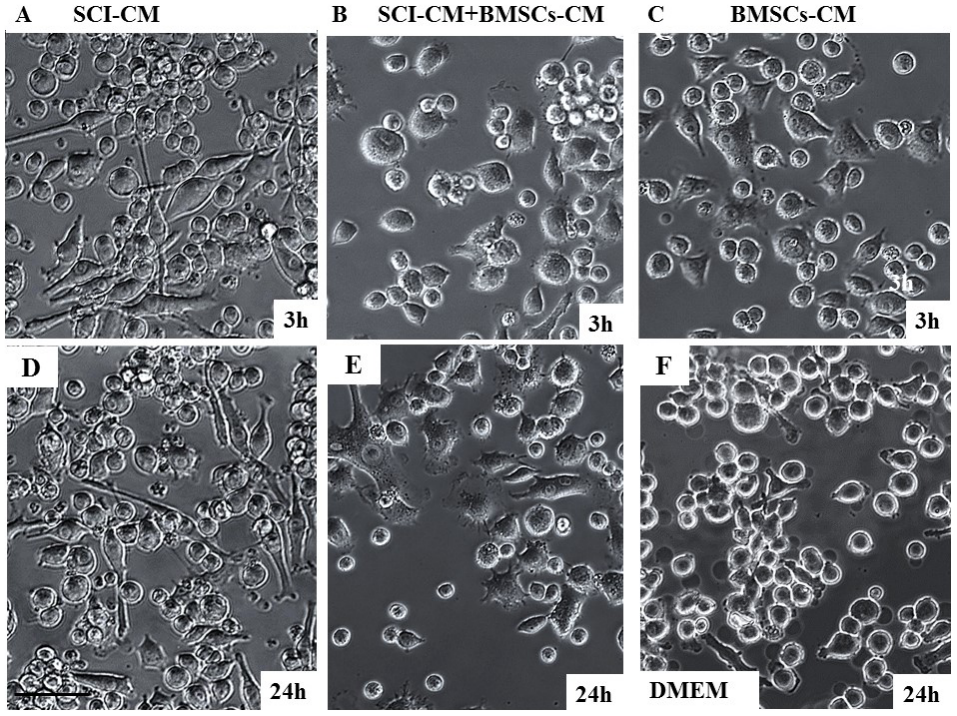
Representative images depicting morphological changes of BV2 microglial cells incubated with SCI-CM, SCI-CM + BMSCs-CM or BMSCs-CM from 3 h (A–C) to 24 h (D–E). Note the prolonged, bipolar-like morphology of BV2 cells after SCI-CM (A), while after SCI-CM + BMSCs-CM (B, E) or BMSCs-CM (C) incubation only few cells changed their shape into oval or stellate types during 3 h treatment. Most significant morphological changes of BV2 cells were observed after incubation with SCI-CM and SCI-CM + BMSCs-CM at 24 h, when BV2 cells revealed multipolar or prolonged cell shapes with hypertrophied cell bodies and ramified morphology (D,E), while treatment with BMSCs-CM resamble the same pattern as 3 h (C). BV2 cells incubated with DMEM during 24 h revealed oval shape, but occasional ramified cells occurred (F) Scale bars A–F = 50 μm.

**Figure 7:**
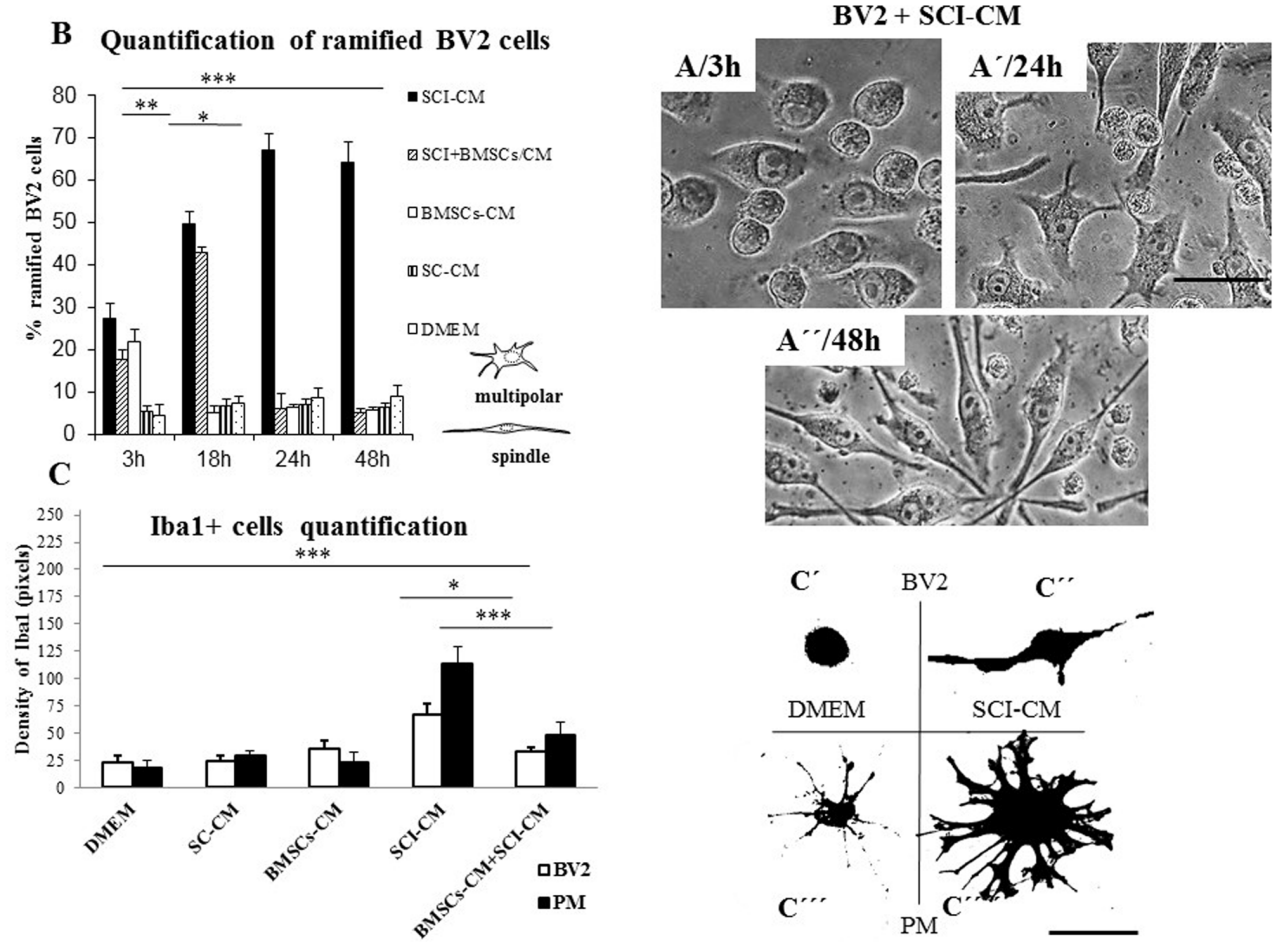
Quantification of stimulated BV2 cells based on their morphological changes (multipolar/spindle shape) (Figures 7A–A″) after incubation with SCI-CM, SCI-CM + BMSCs-CM, BMSCs-CM and SC-CM from 3 h to 48 h. Note the higher % of ramified BV2 cells in time after incubation with SCI-CM (B). Quantification of Iba1+ microglia morphological changes within identical fields (C′ and C′′′′) treated with DMEM, SCI-CM, SCI-CM + BMSCs-CM or BMSCs-CM (C). Note, representative morphology of BV2 cells with round oval shape (C′) and PM with small soma extending to few long thin processes treated with DMEM (C′′′), while after treatment with SCI-CM, BV2 cells with enlarged bipolar morphology (C″) and PM with hypertopied multipolar cell body and retracted thick processes occurred. Data are represented as mean ± SEM. *P < 0.1, **P < 0.01, *** P < 0.001, one-way ANOVA followed by Tukey-Kramer test. Scale bars A–A″, C′–C′′′′ = 20 μm.

Chemotaxis assays confirmed increased numbers of migrated microglia with significantly enlarged nuclei after SCI-CM treatment (Figure 5B) compared with microglia nuclei after DMEM treatment (Figure 5A′). Our next aim was therefore to investigate the morphological changes in stimulated microglia in a time-dependent manner. We compared the effects of: i) SCI-CM, ii) SCI-CM + BMSCs-CM and iii) BMSCs-CM administration on BV2 cell morphology from 3 h to 24 h (Figures 6 A–E). The first significant changes were observed in the presence of SCI-CM, where most BV2 cells revealed a prolonged bipolar-like or stellate-like shape (Figures 6A, 7A). Addition of SCI-CM + BMSCs-CM or BMSCs-CM alone stimulated a lower number of BV2 cells, the latter changing cells from round to oval or multipolar cells (Figures 6B, 6C). After further incubation (24 h) significant morphological variability in BV2 cells begins to show, corresponding to different conditioned media exposure. The most significant impact followed after SCI-CM incubation, when the majority of BV2 cells revealed multipolar or prolonged cell shapes with hypertrophied cell bodies and ramified morphology (Figures 6D, 7A′). A similar pattern was seen in SCI-CM + BMSCs-CM, but only in a small number of cells (Figure 6E). In the case of BMSCs-CM, round to oval cells dominated, apart from a few that had a differentiated pattern, similar as at 3 hr (Figure 6C). After 48 h incubation, activated BV2 cells with elongated morphology remained only after SCI-CM stimulation (Figure 7A″). We could follow the time-dependent (3 h–48 h) morphological changes in BV2 cells after SCI-CM stimulation (Figures 7 A–A″). The percentages of ramified (multipolar or spindle-shaped) BV2 cells stimulated with SCI-CM, SCI + BMSCs/CM, BMSCs-CM and SC-CM after 3 h were respectively: 27.4 ± 3.6, 17.6 ± 2.2, 21.78 ± 3.1, 5.2 ± 1.4; at 18 h: 49.77 ± 2.7, 42.9 ± 1.4, 5 ± 1.8, 6.7 ± 1.5; at 24 h: 67.14 ± 3.8, 6.01 ± 1.8, 6.2 ± 0.9, 7.1 ± 1.1; and at 48 h: 64.2 ± 4.9, 5 ± 1.1, 5.6 ± 0.8, 6.4 ± 0.9 (Figure 7B).

**Figure 8:**
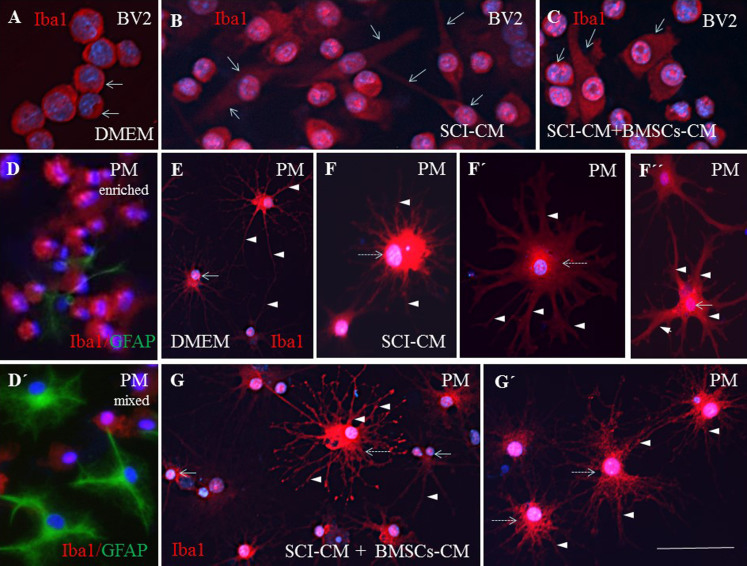
Immunohistochemistry of Iba1+ BV2 cells (A–C) and PM (E–G′) corresponding to different conditioned media exposure. The round BV2 cells treated with DMEM (A, arrows) changed after SCI-CM exposure to bipolar cells with thin processes (B, arrows), while after BMSCs-CM + SCI-CM cells were round or with partial elongated cell body, but the processes had disappeared (C, arrows). The PM treated with SCI-CM demonstrated a significant shift from resting microglia with a small soma and ramified spread out thin and long processes (E, arrowheads), to activated forms characterized by marked cellular and nuclear hypertrophy (F, dashed arrow) with retracted and thickened radially projecting processes (F′–F″, arrowheads). Note, following BMSCs-CM + SCI-CM incubation, many hypertrophied microglia (G,G′, intermitted arrows) with ramified processes retained (G–G′), while rich fine branches, closest to the cell soma, began to thicken. Evaluation of PM purity with Iba1 and GFAP antibodies (D, D′). Scale bars A–G′ = 50 μm.

To further compare the morphological changes in stimulated BV2 cells (Figures 8A–C) and PM (Figures 8E–G′), in each conditioned group we used Iba1 antibody, confirming the positivity in both control and stimulated populations after 24 h. The PM treated with SCI-CM demonstrated a significant shift from resting cells with small soma and ramified, spread-out, thin and long processes, (Figure 8E) to activated forms characterized by marked cellular hypertrophy and thick, short and radially-projecting processes (Figures 8 F′–F″). Similarly, PM incubated in BMSCs-CM and SC-CM showed many hypertrophied microglia with richly-ramified processes (Figures 8 G–G′). Morphological changes were further confirmed using quantification analysis of Iba1+ microglia response (Figure 7), also revealing morphological disparities between BV2 and PM microglia activation. While BV2 cells changed from oval to multipolar or spindle-like forms (Figures 7A,B), a large number of PM transformed from small, less-ramified cells to oval-amoeboid shaped Iba1+ microglia with hypertrophied soma and retracted gross processes with rich branching (Figures 7C–C′′′′). However, in all experimental groups we could detect common intermediate forms of microglia. We therefore quantified the density of all morphological forms of Iba1+ microglia (Figures 7C′–C′′′) that occurred after treatment with DMEM and different combinations of CM. Thus, the PM with thick processes (Figure 7C′′′′) or enlarged bipolar or multipolar BV2 cells (Figure 7C″) occupied more space (expressed in pixel values within identical fields) after SCI-CM (Figure 7C) than after the other CM treatments. To confirm morphological characteristics of microglia after different CM treatment we have defined five following parameters: soma diameter, soma area, process diameter and length, and process length in relation to the soma diameter (Supplementary data 3).

**Figure 9:**
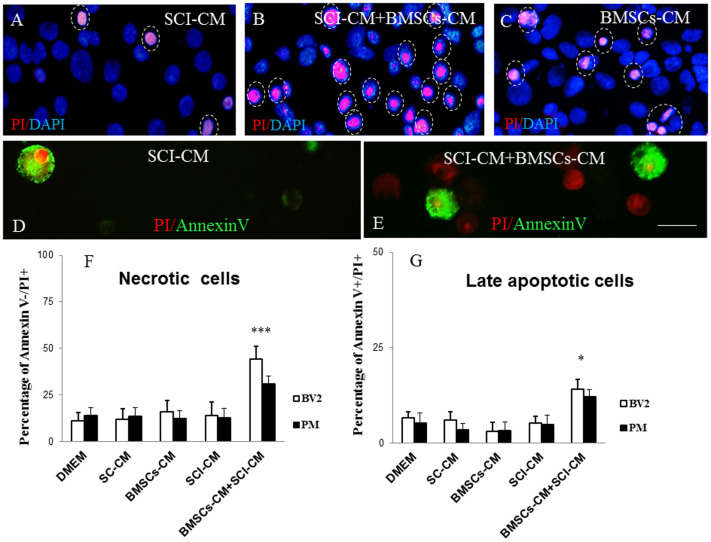
PI and Annexin V (AV) labeling of BV2 cells (D, E) counterstained with Dapi (A–C). Note, the highest number of late apoptotic or necrotic (PI+/AV+, PI+/AV−) BV2 cells exposed to SCI + BMSCs/CM (B, E) when compared to incubation with SCI-CM with occasional late apoptotic (PI+/AV+) and early apoptotic cells (PI−/AV+) (A,D) or BMSCs-CM (C) (purple, indicated by dashed circles) at 48 h. PI/red, Annexin V/green labeled BV2 cells (D,E). Bar graph reporting the percentage of necrotic and late apoptotic microglia cells after different CM treatment after 48h in vitro incubation. Data are represented as mean ± SEM. *P < 0.1 **P < 0.01, *** P < 0.001, one-way ANOVA followed by Tukey-Kramer test (F,G). Scale bars A-E = 20 μm.

The viability test with Propidium iodide (PI) and Annexin-V (AV) confirmed the time-dependent cytotoxic effect of BMSCs-CM when added in combination with SCI-CM (44 ± 7% of PI+ and 12 ± 2.5% of AV+ BV2 cells; 21 ± 4% of PI+ and 10.1 ± 3.9% of AV+ PM) (Figures 9B, E), but not alone (18 ± 6% of PI+ and 3 ± 2.3% AV+ BV2 cells; 12.3 ± 4.4% of PI+ and 5 ± 2.1% AV+ BV2 cells) (Figure 9C), whereas SCI-CM (14 ± 7% of PI+ and 7 ± 1.9% of AV+ BV2 cells; 12.7 ± 8.6% of PI+ and 8 ± 2.9% of AV+ PM) (Figures 9A, D) or DMEM (11.2 ± 4.4% PI+ and 8.6 ± 1.5% of AV+ BV2 cells; 14.1 ± 4.2% of PI+ and 5.2 ± 2.7% of AV+ PM) had a low influence on BV2 cell viability, as revealed by PI+ and AV+ cells. PI is a membrane-impairment dye that is generally excluded from viable cells, while phosphatidylserine cell surface membrane exposure is typical for cell apoptosis and could be detected by its binding to the protein Annexin-V. Thus, in our case it is most probably that PI labeling discriminated necrotic cells PI+/AV−) (Figure 9F) that had lost membrane integrity, while Annexin–V and PI labeling distinguished late apoptotic cells (PI+/AV+) (Figure 9G). Only occasional Annexin-V (AV+/PI− < 2%) labeled BV2 and PM cells were detected when cultured with SCI-CM or BMSCs-CM.

### Inhibition of NO with BMSCs-CM

**Figure 10:**
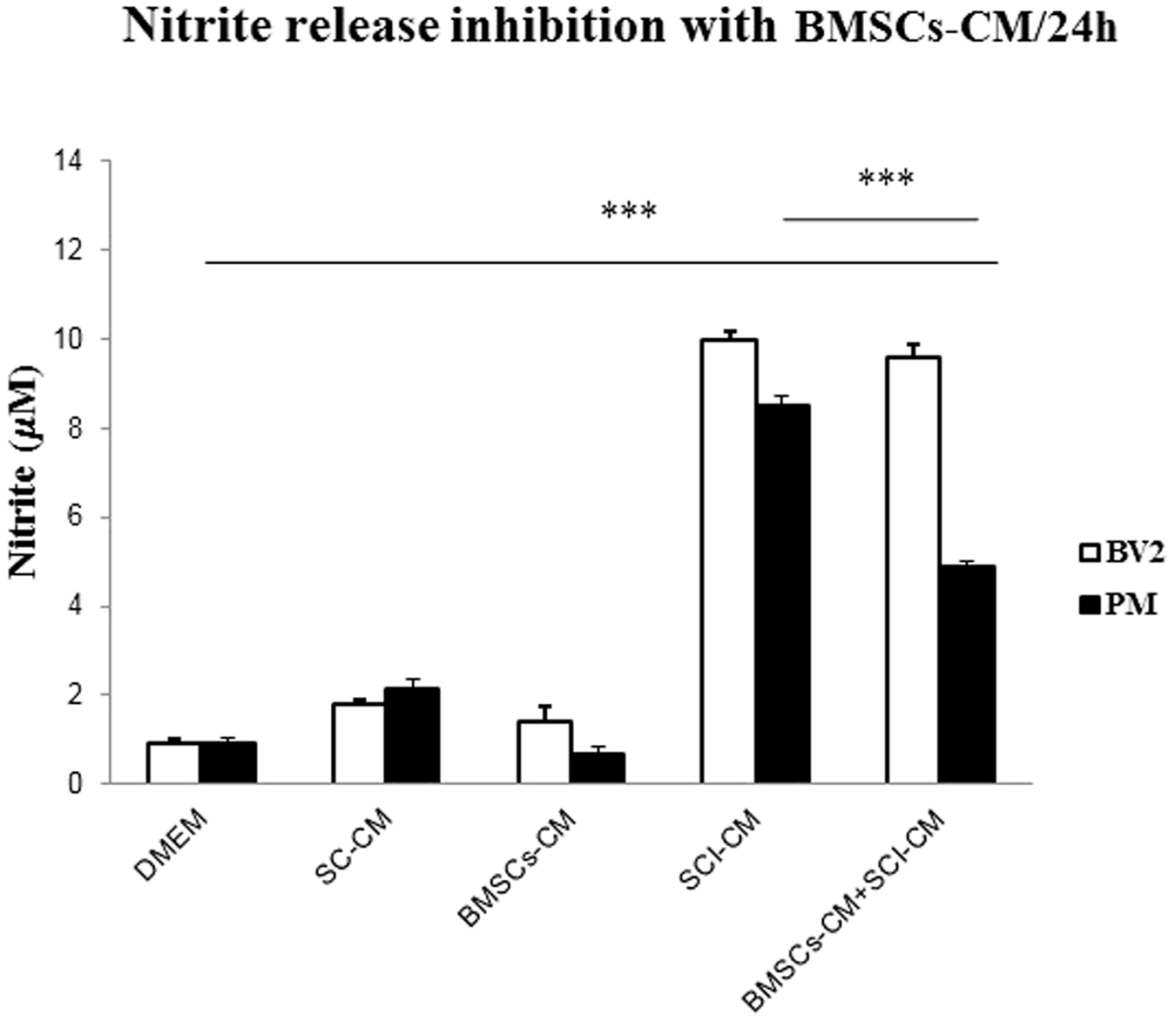
NO release from BV2 (A) cells and PM (B) into the culture media after different conditioned media exposure after 24 h. Note, significant increase of NO in BV2 cells and PM after SCI-CM when compared to DMEM, SC-CM, and BMSCs-CM incubation, while significant NO decrease in PM, not in BV2 cells occurred after BMSCs-CM + SCI-CM treatment. Data are represented as mean μM ± SEM. *P < 0.01, *** P < 0.001, one-way ANOVA followed by Tukey-Kramer test.

BV2 cells and PM robustly increased NO release into the culture media after SCI-CM treatment. Peak release of 9.98 ± 0.17 μM (p < 0.001) NO from BV2 cells and 8.5 ± 0.27 μM (p < 0.001) NO from PM at 24 h was significantly decreased in PM to 4.86 ± 0.13 μM (p < 0.001) after BMSCs-CM-CSI-CM treatment, but not in BV2 cells, which released 9.6 ± 0.2 μM (p < 0.001) NO. DMEM, SC-CM and BMSCs-CM induced low levels of NO in BV2 cells and PM respectively (0.91 ± 0.2, 1.82 ± 0.06, 1.41 ± 0.3 μM and 0.92 ± 0.12, 2.14 ± 0.21, 0.69 ± 0.1 μM) (Figure 10).

## Discussion

The early phase of cellular inflammation is comprised principally of neutrophils (peaking 1 day post-injury), macrophages/microglia (peaking 7 days post-injury) and T cells (peaking 9 days post-injury)^51^. This acute phase is decomposed by the production of pro-inflammatory mediators produced by resting microglia, and neutrophils (0 to 1 days), which is correlated to necrosis, followed by apoptosis processes (peaking 3 days post-injury) then demyelination (peaking 7 days post-injury). Anti-inflammatory cytokines are produced rapidly and peaking at 3 days. The late phase of cellular inflammation was detected after 14 days post-injury, peaked after 60 days post-injury and remained detectable throughout 180 days post-injury for macrophages/activated microglial cells and neutrophils^51^. Understanding this role is complicated by the observations that while some aspects of post-traumatic inflammation in the spinal cord are clearly detrimental, other delayed inflammatory aspects may facilitate repair mechanisms^52^. For example, the inflammatory response is critical for the clearance of cellular debris, which can prevent the regeneration of surviving neurons. However, over- activation of the inflammatory response can damage healthy tissue and exacerbate the injury^53^,^54^. Thus in this context, a therapeutic approach that has to be designed needs to be focused more on the secondary injury process where the chronic phase of inflammation occurs and needs to be controlled. Previously, it was proposed that, BMSCs could be potentially transplanted during both acute and chronic phases of SCI, because they modify the inflammatory response in the acute setting and may reduce the inhibitory effects of scar tissue in the subacute/chronic phase to provide a permissive environment for axonal extension. However, recently the main attention is focused on their delivery during acute inflammatory processes. Pre-clinical studies showed that acute transplantation of human BMSCs after SCI in rats increases axonal growth and improved locomotor function^55^. Similarly, in clinical trials using BMSCs (alone or in combination with granulocyte-macrophage colony stimulating factor) with over 10 weeks of follow-up moderate functional recovery was noticed in acute and subacute SCI groups^56^,^57^,^58^, while no improvement was detected in chronically treated patients^59^. On the other hand some experimental studies showed that chronically injured spinal cord axons can regenerate through the gliotic scar only in the presence of local growth-stimulating factors. For example, genetically modified BMSCs secreting neurotrophin-3 (NT-3) injected into the central lesion site were able to induce penetration of modest number of axons through the scar tissue^60^. In general, BMSCs represent a safe, feasible, and reliable method of cellular transplantation for SCI with no fear of tumor formation.

In this context, we focus our attention on factors that can be produced by BMSCs. BMSCs factors are known to ameliorate disease in various animal models of neuroinflammation could control *in vitro* SCI-CM–induced BV2 chemotaxis^29^,^58^, NO release, as well as morphological changes in activated microglia. Similarly to microglia, BMSCs are attracted towards areas of tissue damage, indicating that microglia may primarily serve as a homing signal^61^. Here we show that BMSCs-soluble factors significantly down-regulated SCI-CM-induced BV2 and PM migration, which confirms their modulatory properties^58^,^60^. Because the chemotactic response was associated with enlarged nuclei as well, we also examined temporal changes in BV2 cell morphology and PM. Our results provide further evidence of a link between migratory response and morphological changes in microglial cells upon exposure to different conditioned media. SCI-CM (lesion site) served as a strong trigger for microglia migration and also caused BV2 cell transformation from round-shaped, semi-adherent cell lines into adherent, stellate–like or long bipolar cells with filopodia production^61^, revealing typical inflammatory response during the entire time period. However, BMSCs that clearly attenuated microglia migration showed time-dependent cytotoxic effects on BV2 cells. A similar study has confirmed that BMSCs inhibit the proliferation of lipopolysaccharide (LPS)-activated BV2 microglia by various effects, which may correlate with our cytotoxic findings^58^. It is well known that BMSCs and microglia cells modulate SCI inflammation and regeneration processes.

The proteomic analysis of BMSCs-CM clearly shows that these cells can produce both immune modulator, neurogenic factors and osteogenic factors as well as differentiating molecules. In fact, using a shot-gun proteomic approach we identified several immune modulators (arginase 1, ST2, galectins) and chemoattractant factors (CLEC11a) known to act towards microglia^62^. The different immune-modulators present in BMSCs-CM *i.e.* arginase, ST2, CCL2 have the ability to shift the polarization of the microglial cells into M2 phenotype which is neuroprotective. Moreover, PDGF, PGF and TGF β are known to increase survival and proliferation of oligodendrocytes^63^. BDNF also increases oligodendrocyte proliferation and BMP 1 signaling mediates astrocyte differentiation of oligodendrocyte progenitor cells^64^. SPARC has been shown to modulate several growth factor signaling cascades (i.e., VEGF (vascular endothelial growth factor), PDGF (platelet-derived growth factor), FGF2 (fibroblast growth factor-2), and TGF (transforming growth factor beta)) and can regulate integrin-mediated adhesion^65^,^66^. The matricellular protein family CCN, which stands for CYR61/CTGF/NOV are suggested to be important players in the modulation of inflammatory cytokines and chemokines production^67^. CCN proteins act alone or in concert with their specific partners in order to regulate the production of cytokines and chemokines. CCN2/CTGF is currently the only CCN family member in which expression has been demonstrated *in vivo* in CNS astrocytes^68^. CCN2 has been demonstrated to bind to TrkA (neurotrophic tyrosine kinase receptor type 1) and p75NTR (p75 neurotrophin receptor), receptors which transduce neurotrophin signals^69^. Similarly, the C1q/TNF-related protein (CTRP) family are also important immune modulators^70^. The presence of ST2, a receptor of IL-33, confirms previous data showing that IL-33 is known to induce proliferation of microglia and enhances the production of pro-inflammatory cytokines, such as IL-1β and TNFα, as well as the anti-inflammatory cytokine IL-10^62^. Galectins were recently shown to be produced by BMSCs with high immunosuppressive activity as well as tissue-inhibitor metalloproteinases^64^,^65^,^66^. Chang et al^70^ have shown that BMSCs release TIMP-1, which would exert an immune modulatory effect on BV2 LPS-activated microglial cells. The authors have shown that in co-culture between BMSCs and BV2 after LPS activation, TIMP-1 secretion downregulates MMP-9 expression in microglial cells. At the same time, BMSCs produce growth and neurotrophic factors^70^. The CLEC11A, also known as stem cell growth factor^64^,^67^, act in conjunction with insulin-like growth-factor binding protein 10^65^,^66^, transforming growth factor beta-1^71^, and SPARC^69^,^70^. Some recent data show moreover that SPARC seems to act as a novel regulator of microglial proliferation, and may play an important role in differently regulating the gray and white matter microglial responses to CNS lesion^45^. Concerning the osteogenic factors produced by BMSCs, these molecules act like osteopontin towards microglial cells in the same way as cytokines, and stimulate their proliferation. Tambuyzer et al^71^ have demonstrated that osteopontin shifts microglia to an alternative functional profile more suited to the immune-balanced microenvironment of the CNS.

Here we confirm that SCI-CM triggers morphological changes in microglia and production of NO inflammatory mediators, while incubation with BMSCs-CM leads to partial attenuation of these processes. The use of morphological change as a readout of microglia activation was supported also by our previous experiment comparing the number of microglia processes to the expression level of P2Y12 (a metabotropic purinergic receptor) under activating conditions^71^. In this study the authors clearly demonstrate a positive correlation between decreased process number (amoeboid morphology) and P2Y12 down-regulation. The present data are consistent with these findings and provide fundamental information on the morphological features of BV2 cells and PM upon SCI-CM activation. Based on Iba1 expression or light microscopy image analysis, we present clear morphological differences between microglia cell lines and primary microglia in terms of soma shape, size and extending processes evaluated following induced activation and BMSCs-CM treatment. Unlike other automated or manual quantification methods of microglia morphology based on various parameters^72^,^73^, we have used simple ImageJ software to quantify the 2D area occupied by each Iba1 positive cell. Thus, hypertrophied microglia and their extended processes that fill larger micro-territory than the resting type proved to be effective enough as a method of capturing microglia in activated state.

Furthermore, we demonstrate that factors released from BMSCs-CM significantly decreased NO levels in SCI-CM treated PM cultures. Although some studies have indicated that MSCs increase NO when exposed to soluble factors from LPS-activated microglia or when co-cultured with stimulated T lymphocytes, we document the opposite effect^10^,^74^. The inhibition of NO levels in activated PM was most likely attributed to the molecule composition of cell-free BMSCs-conditioned medium which unlike mesenchymal cells is unable to produce additional NO. However, the levels of NO were significantly higher in both stimulated microglia cells treated with BMSCs. That may be caused by factors released from SCI-CM, but this needs to be confirmed in further experiments.

Taken together, our preliminary proteomic data obtained with BMSCs-CM confirm the high modulatory potential of these cells on inflammation, microglia polarization and neurite outgrowth activity and could be used as a therapeutic cocktail to prevent the chronic phase of inflammation.

## Supplementary Material

Supplementary Information Supplemntary data

## Figures and Tables

**Table 1:** Expression of surface markers in BMSCs after Passage 1 and 3

Surface antigens			
	CD29	CD90	CD45
**1 passage**	++	++	+
**3 passage**	+++	+++	−

− no expression (<10%); + weak expression (11–40%); ++ moderate expression (41–70%); +++ strong expression (>71%).

**Table 2:** Immune and neurotrophic factors identified by shot-gun analyses from MSCs and SCI. Sequence coverage are established from shot gun analyses using LTQ-Orbitrap XL mass spectrometer and based on protein identification with a protein threshold of 99% (FDR 0.1%) and peptide threshold of 97% (0.1%) and contained at least 2 identified peptides (see Supplementary data 2)

ProteinFamily	Protein Name	Accession Number	SequenceCoverage
**Chemokines**	CCL3	P50229	35%
	CXCL2	P303484	49%
	C1qc	P31722	16%
	cC1qR	O35796	20%
	MIF	P30904	36%
**Immune**	ST2(ILRL1)	Q62611	24%
**Regulators**	CLEC11a	O88201	20%
	Gal 1	P11762	64%
	Gal 3	P08699	9%
	Arginase 1	P07824	9%
**CD**	CD9	P40241	15%
	CD14	Q63691	17%
	CD59	P27274	30%
**GrowthFactors**	CTGF	Q9R1E9	56%
	BDNF	P23363	12%
	TGFb1	P17246	16%
	TGF b2	Q07257-2	18%
	TGF b3	Q07258	12%
	PGF	Q63434	22%
	PDGF	Q5RJP7	19%
	IGFbp7	F1M9B2	54%
	BMP1	F1M798	4%
	Epdr1	Q5XII0	19%
	C1q/TNF5	Q5FVH0	28%
**Neurotrophic Factors**	Neurotrimin	Q62718	16%
	GAP43	P07936	63%
	Calumenin	O35783	36%
**Neural Migration Factor**	Neuroplastin	P97546	10%
	Neuropilin 2	035276	3.7%
	Neuromodulin	P07936	63%
	NOV	Q9QZQ5	30%
**Metalloproteinase**	TIMP-1	P30120	71%
	TIMP-2	P30121	54%
**Osteogenic Factors**	Osteoglycin	D3VZB7	13%
	Osteopontin	PO8721	21%
	Périostin	D3ZAF5	66%
**Differentiating factors**	SPARC	P16975	60%
	FAM3C	Q10F4	9%
	Cornifin	Q63532	11%
