# Supplementary material for: Modulation properties of factors released by bone marrow stromal cells on activated microglia: an in vitro study
Source: Sci Rep. 2014 Dec 19;4:7514. doi: 10.1038/srep07514 (PMC5378994; doi:10.1038/srep07514)
Supplement: Supplementary Information — Supplemntary data [file srep07514-s1.pdf]

Modulation properties of factors released by bone marrow stromal cells on activated microglia: an *in vitro* study

Dasa Cizkova[1,2]*, Stéphanie Devaux[1]*, Françoise Le Marrec-Croq[1], Julien Franck[1], Lucia Slovinska[2], Juraj Blasko[2], Jan Rosocha[3], Timea Spakova[3], Christophe Lefebvre[1], Isabelle Fournier[1], Michel Salzet[1]

Supplementary data 1

sp|P50229|CCL3_RAT (100 %), 10 335,1 Da
C-C motif chemokine 3 OS=Rattus norvegicus GN=Ccl3 PE=1 SV=1
3 exclusive unique peptides, 3 exclusive unique spectra, 3 total spectra, 32/92 amino acids (35 % coverage)

MKVSTAALAV LLCTMALWNE VFS**APYGADT** **PTACCFSYGR** QIPRKFIADY FETSSLCSQP
GVIFLTKRNR QICADPK**ETW** **VQEYITELEL** **NA**

sp|P30348|CXCL2_RAT (100 %), 10 782,5 Da
C-X-C motif chemokine 2 OS=Rattus norvegicus GN=Cxcl2 PE=1 SV=1
2 exclusive unique peptides, 2 exclusive unique spectra, 2 total spectra, 49/100 amino acids (49 % coverage)

MAPPTRQLLN AVLVLLLLLA TNHQGTGVVV ASELR**CQCLT** **TLPR**VDFK**NI** **QSLTVTPPGP**
HCAQTEVIAT **LKDGHEVCLN** **PEAPLVQR**IV QKILNKGKAN

sp|P31722|C1QC_RAT (100 %), 25 685,9 Da
Complement C1q subcomponent subunit C OS=Rattus norvegicus GN=C1qc PE=1 SV=2
3 exclusive unique peptides, 3 exclusive unique spectra, 6 total spectra, 40/245 amino acids (16 % coverage)

MVVGTSCQPQ HGLYLLLLLL ALPLRSQANA GCYGIPGMPG LPGTPGKDGH DGLQGPKGEP
GIPAIPGTQG PKGQKGEPGM PGHRGKNGPM GTSGSPGDPG PRGPPGEPGE EGRYKQK**HQS**
VFTVTRQTAQ **YPAANGLVKF** **NSAITNPQGD** **YNTNTGK**FTC KVPGLYYFVH HTSQTANLCV
QLLLNNAKVT SFCDHMSNSK QVSSGGVLLR LQRGDEVWLA VNDYNGMVGT EGSDSVFSGF
LLFPD

sp|P18418|CALR_RAT (100 %), 47 997,0 Da
Calreticulin OS=Rattus norvegicus GN=Calr PE=1 SV=1
5 exclusive unique peptides, 5 exclusive unique spectra, 6 total spectra, 90/416 amino acids (22 % coverage)

MLLSVPLLLG LLGLAAADPA IYFK**EQFLDG** **DAWTNR**WVES KHKSDFGKFV LSSGK**FYGDQ**
EKDKGLQTSQ **DAR**FYALSAR FEPFSNKGQT LVVQFTVKHE Q**NIDCGGGYV** **KLFPGGLDQK**
DMHGDSEYNI MFGPDICGPG TKKVHVIFNY KGKNVLINKD IRCKDDEFTH LYTLIVRPDN
TYEVKIDNSQ VESGSLEDDW DFLPPKK**IKD** **PDAAKPEDWD** **ERAKIDDPTD** **SKPEDWDKPE**
HIPDPDAKKP EDWDEEMDGE WEPPVIQNPE YKGEWKPRQI DNPDYKGTWI HPEIDNPEYS
PDANIYAYDS FAVLGLDLWQ VKSGTIFDNF LITNDEAYAE EFGNETWGVT KAAEKQMKDK
QDEEQRLKEE EEDKKRKEEE EAEDKEDEDD RDEDEDEEDE KEEDEEDATG QAKDEL

sp|P30904|MIF_RAT (100 %), 12 477,4 Da
Macrophage migration inhibitory factor OS=Rattus norvegicus GN=Mif PE=1 SV=4
3 exclusive unique peptides, 4 exclusive unique spectra, 4 total spectra, 41/115 amino acids (36 % coverage)

M**PMFIVNTNV** **PRASVPEGFL** **SELTQQLAQA** **TGK**PAQYIAV HVVPDQLMTF SGTSDPCALC
SLHSIGKIGG AQNRNYSK**LL** **CGLLSDR**LHI SPDRVYINYY DMNAANVGWN GSTFA

sp|Q62611-2|ILRL1_RAT (100 %), 38 090,4 Da
Isoform B of Interleukin-1 receptor-like 1 OS=Rattus norvegicus GN=Il1rl1
6 exclusive unique peptides, 7 exclusive unique spectra, 8 total spectra, 82/336 amino acids (24 % coverage)

MIGKWRMGLW ALAILTVPMY FIVTEGRK**TS** **WGLENEALIV** **R**CPQR**GGAIN** **PVEWYYSNTN**
ERIPTQKRNR IFVSRDRLKF LPAK**VEDSGI** **YTCVIRSPES** **IK**TGSLNVTI YKRPPNCKIP
DYMMYSTVDG SDKNSKITCP TIALYNWTAP VQWFKNCKAL QGPRFR**AHMS** **YLFIDKVSHV**
DEGDYTCRFT HTENGTNYIV TATRSFTVEE KGFSTFPVIT NPPHNYTVEV EIGKTANIAC
SACFGTASQF VAVLWQINKT RIGSFGKARI QEEKGPNKSS SNGMICLTSL LR**ITGVTDKD**
FSLKYDCVAM NHHGVIRHPV RLRRKQPSKE CLSQIA

sp|O88201|CLC11_RAT (100 %), 36 387,5 Da
C-type lectin domain family 11 member A OS=Rattus norvegicus GN=Clec11a PE=2 SV=1
6 exclusive unique peptides, 6 exclusive unique spectra, 6 total spectra, 67/328 amino acids (20 % coverage)

MQAAWLLGAL LVPHLLSFGH GARGHGKEWE GVWGGALEEE RDRESLMLK**N** **LQEALGLPTG**
VGNKDNLAEN SEGKEVWEAT ETQGEEEEEE TTTTPSSSPT PFPSPSPTSE DTVTYILGR**L**
ASLDAGLHQL **HIR**LHVLDTR **VVELTQGLR**R LRDAASDTRD SVQALKEVQV RSEQEHGRLE
GCLKGLRLGH KCFLLSR**DFE** **TQAAAQAR**CK AR**GGSLAQPA** **DR**QQMDALSR YLRAALAPYN
WPVWLGVHDR RSEGLYLFEN GQR**VSFFAWH** **R**ALSPESGAQ PSAASHPLSP DQPNGGILEN
CVAQASDDGS WWDHDCERRL YFVCEFPF

sp|P11762|LEG1_RAT (100 %), 14 857,2 Da
Galectin-1 OS=Rattus norvegicus GN=Lgals1 PE=1 SV=2
9 exclusive unique peptides, 10 exclusive unique spectra, 18 total spectra, 86/135 amino acids (64 % coverage)

M**ACGLVASNL** **NLKPGECLKV** **RGELAPDAKS** **FVLNLGKDSN** **NLCLHFNPR**F NAHGDANTIV
CNSK**DDGTWG** **TEQR**ETAFPF QPGSITEVCI TFDQADLTIK **LPDGHEFKFP** **NRLNMEAINY**
MAADGDFKIK CVAFE

sp|P08699|LEG3_RAT (100 %), 27 202,1 Da
Galectin-3 OS=Rattus norvegicus GN=Lgals3 PE=1 SV=4
3 exclusive unique peptides, 4 exclusive unique spectra, 5 total spectra, 23/262 amino acids (9 % coverage)

MADGFSLNDA LAGSGNPNPQ GWPGAWGNQP GAGGYPGASY PGAYPGQAPP GGYPGQAPPS
AYPGPTGPSA YPGPTAPGAY PGPTAPGAFP GQPGGPGAYP SAPGAYPSAP GAYPATGPFG
APTGPLTVPY DMPLPGGVMP RMLITIIGTV KPNANSITLN FK**KGNDIAFH** **FNPR**FNENNR
RVIVCNTKQD NNWGREERQS AFPFESGKPF K**IQVLVEADH** **FK**VAVNDVHL LQYNHRMKNL
REISQLGIIG DITLTSASHA MI

---

sp|P07824|ARGI1_RAT (100 %), 34 973,7 Da
Arginase-1 OS=Rattus norvegicus GN=Arg1 PE=1 SV=2
3 exclusive unique peptides, 3 exclusive unique spectra, 3 total spectra, 30/323 amino acids (9 % coverage)

MSSKPKPIEI IGAPFSKGQP RGGVEKGPAA LRKAGLVEKL KETEYNVRDH GDLAFVDVPN
DSPFQIVKNP RSVGKANEQL AAVVAETQKN GTISVVLGGD HSMAIGSISG HARVHPDLCV
IWVDAHTDIN TPLTTSSGNL HGQPVAFLLK ELKGKFPDVP GFSWVTPCIS AK**DIVYIGLR**
DVDPGEHYII **K**TLGIKYFSM TEVDKLGIGK VMEETFSYLL GRKKRPIHLS FDVDGLDPVF
TPATGTPVVG GLSYR**EGLYI** **TEEIYK**TGLL SGLDIMEVNP TLGKTPEEVT RTVNTAVALT
LSCFGTKREG NHKPETDYLK PPK

sp|P40241|CD9_RAT (100 %), 25 215,7 Da
CD9 antigen OS=Rattus norvegicus GN=Cd9 PE=1 SV=2
2 exclusive unique peptides, 3 exclusive unique spectra, 4 total spectra, 35/226 amino acids (15 % coverage)

MPVKGGSKCI KYLLFGFNFI FWLAGIAVLA IGLWLRFDSQ TKSIFEQETN HSSFYTGVYI
LIGAGALMML VGFLGCCGAV QESQCMLGLF FGFLLVIFAI EIAAAVWGYT HKDEVIKELQ
EFYKDTYQKL RNKDEPQRET LK**AIHMALNC** **CGIAGGVEQF** **ISDICPKKQV** **LESFQVK**SCP
DAIDEVFHSK FHIIGAVGIG IAVVMIFGMI FSMILCCAIR RSREMV

sp|Q63691|CD14_RAT (100 %), 40 055,2 Da
Monocyte differentiation antigen CD14 OS=Rattus norvegicus GN=Cd14 PE=2 SV=2
4 exclusive unique peptides, 5 exclusive unique spectra, 7 total spectra, 65/372 amino acids (17 % coverage)

MKLMLGLLLL PLTLVHA**SPA** **TPEPCELDQD** **EESVR**CYCNF SDPQPNWSSA FLCAGAEDVE
FYGGGRSLEY LLKRVDTEAN LGQYTDIIRS LPLKRLTVRS AR**VPTQILFG** **TLR**VLGYSGL
RELTLENLEV TGTALSPLLD ATGPDLNTLS LRNVSWATTD TWLAELQQWL KPGLKVLSIA
QAHSLNFSCK **QVGVFPALAT** **LDLSDNPELG** **EK**GLISALCP HKFPTLQVLA LRNAGMETTS
GVCSALAAAR **VPLQALDLSH** **NSLR**DTAGTP SCDWPSQLNS LNLSFTGLEH VPKGLPAKLS
VLDLSYNRLD RKPRPEELPE VGSLSLTGNP FLHSESQSEA YNSGVVIATA LSPGSAGLSG
TLALLLGHRL FV

sp|P27274|CD59_RAT (100 %), 13 790,5 Da
CD59 glycoprotein OS=Rattus norvegicus GN=Cd59 PE=1 SV=2
4 exclusive unique peptides, 4 exclusive unique spectra, 9 total spectra, 38/126 amino acids (30 % coverage)

MRARRGFILL LLLAVLCSTG VSLR**CYNCLD** **PVSSCK**TNST CSPNLDACLV AVSGK**QVYQQ**
CWRFSDCNAK FILSR**LEIAN** **VQYRCCQADL** **CNK**SFEDKPN NGAISLLGKT ALLVTSVLAA
ILKPCF

sp|Q9R1E9|CTGF_RAT (100 %), 37 755,2 Da
Connective tissue growth factor OS=Rattus norvegicus GN=Ctgf PE=2 SV=1
19 exclusive unique peptides, 20 exclusive unique spectra, 45 total spectra, 194/347 amino acids (56 % coverage)

MLASVAGPVS LALVLLLCTR PATG**QDCSAQ** **CQCAAEAAPR** **CPAGVSLVLD** **GCGCCR**VCAK
QLGELCTERD PCDPHK**GLFC** **DFGSPANRKI** **GVCTAKDGAP** **CVFGGSVYRS** **GESFQSSCK**Y
QCTCLDGAVG CVPLCSMDVR **LPSPDCPFPR** RVKLPGK**CCE** **EWVCDEPKDR** **TVVGPALAAY**
RLEDTFGPDP **TMMR**ANCLVQ TTEWSACSKT CGMGISTR**VT** **NDNTFCR**LEK QSR**LCMVRPC**
EADLEENIKK GKKCIRTPK**I** **AKPVKFELSG** **CTSVK**TYRAK FCGVCTDGRC CTPHRTTTLP
VEFKCPDGEI MKKNMMFIK**T** **CACHYNCPGD** **NDIFESLYYR** KMYGDMA

sp|P17246|TGFB1_RAT (100 %), 44 330,0 Da
Transforming growth factor beta-1 OS=Rattus norvegicus GN=Tgfb1 PE=1 SV=1
4 exclusive unique peptides, 4 exclusive unique spectra, 4 total spectra, 64/390 amino acids (16 % coverage)

MPPSGLRLLP LLLPLPWLLV LTPGRPAAGL STCKTIDMEL VKRKRIEAIR GQILSKLRLA
SPPSQGEVPP GPLPEAVLAL YNSTRDR**VAG** **ESADPEPEPE** **ADYYAK**EVTR VLMVDRNNAI
YDKTKDITHS IYMFFNTSDI REAVPEPPLL SRAELRLQRF K**STVEQHVEL** **YQK**YSNNSWR
YLGNR**LLTPT** **DTPEWLSFDV** **TGVVRQWLNQ** **GDGIQGFR**FS AHCSCDSKDN VLHVEINGIS
PKRRGDLGTI HDMNRPFLLL MATPLERAQH LHSSRHRRAL DTNYCFSSTE KNCCVRQLYI
DFRKDLGWKW IHEPKGYHAN FCLGPCPYIW SLDTQYSKVL ALYNQHNPGA SASPCCVPQA
LEPLPIVYYV GRKPKVEQLS NMIVRSCKCS

sp|Q63434|PLGF_RAT (100 %), 17 681,3 Da
Placenta growth factor OS=Rattus norvegicus GN=Pgf PE=1 SV=1
2 exclusive unique peptides, 2 exclusive unique spectra, 2 total spectra, 34/158 amino acids (22 % coverage)

MLAMKLFTCF LQVLAGLAVH SQGALSAGNN STEMEVVPFN EVWGRSYCRP MEK**LVYIADE**
HPNEVSHIFS **PSCVLLSR**CS GCCGDEGLHC VALKTANITM QILKIPPNRD PHSYVEMTFS
QDVLCECRPI LETTKAERRK TKGKRKQSK**T** **PQTEEPHL**

sp|Q5RJP7|PGFRL_RAT (100 %), 41 932,9 Da
Platelet-derived growth factor receptor-like protein OS=Rattus norvegicus GN=Pdgfrl PE=2 SV=1
4 exclusive unique peptides, 4 exclusive unique spectra, 4 total spectra, 73/375 amino acids (19 % coverage)

MKVWLLLGLL LLHEALGDVA GQHPPKNKRP KEQGENRIKP TNKKAKPKIP KIKDRDTADS
APKSQSIMMQ AMDNGRFQKP AATVSLMAGQ SVELRCKGSK VEWSYPAYLD TFKDSRLTVK
QNERYGQLTL VNSTTADTGE FSCWERLCNG YICRRDEART GSTYIFFTEK GELFVPSPSY
FDVVYLNPDR QAVVPCRVTA PSAKVTLHRE FPAKEIPANG TDIVYDMKRG FVYLQPHSDH
QGVVYCKAEA GGKSQISVKY QLLYVEVPSG PPSTTILASS NKVRGGDDIS VLCTVLGEPD
VEVEFRWIFP GQKDERPVTI QDTWRLIHRG LGHTTRISQS VITVEDFETI DAGYYICTAQ
NLRGQTTVAT TVEFS

tr|F1M9B2|F1M9B2_RAT (100 %), 28 948,7 Da
Insulin-like growth factor binding protein 7, isoform CRA_b OS=Rattus norvegicus GN=Igfbp7 PE=4 SV=2
13 exclusive unique peptides, 17 exclusive unique spectra, 41 total spectra, 152/281 amino acids (54 % coverage)

MERSPRALLL GAAGLLLLLL PLSSSSSSDA CGPCVPASCP ALPPLGCPLG ETRDACGCCP
VCARGEGEPC GGGAAGRGHC APGMECVKSR KRRKGKAGAA AGGPATLAVC VCKSRYPVCG
SDGVTYPSGC QLRAASLRAE SRGEKAITQV SKGTCEQGPS IVTPPKDIWN ITGAKVYLSC
EVIGIPTPVL IWNKVKRDHS GVQRTELLPG DRENLAIQTR GGPEKHEVTG WVLVSPLSKE
DTGEYECHAS NSQGQASASA KITVVDAIHE IPVKKGEGAQ L

tr|F1M798|F1M798_RAT (100 %), 111 649,0 Da
Bone morphogenetic protein 1, isoform CRA_b OS=Rattus norvegicus GN=Bmp1 PE=3 SV=2
2 exclusive unique peptides, 2 exclusive unique spectra, 2 total spectra, 37/990 amino acids (4 % coverage)

MPGVARPPLP LLSLPLLLLL LLPRAGRPLD LADYTYDLGE EDAPELLNYK DPCKAAAFLG
DIALDEEDLR AFRVQQAAVL RQQTAQRSSI KAAGNSSALG RQSTSGQPQR GSRGRWRSRP
RSRRAATSRP ERVWPDGVIP FVIGGNFTGS QRAVFRQAMR HWEKHTCVTF LERTDEDSYI
VFTYRPCGCC SYVGRRGGGP QAISIGKNCD KFGIVVHELG HVIGFWHEHT RPDRDRHVSI
VRENIQPGQE YNFLKMEVQE VESLGETYDF DSIMHYARNT FSRGIFLDTI VPKYEVNGVK
PSIGQRTRLS KGDIAQARKL YKCPACGETL QDSTGNFSSP EYPNGYSAHM HCVWRISVTP
GEKIILNFTS MDLYRSRLCW YDYVEVRDGF WRKAPLRGRF CGGKLPEPIV STDSRLWVEF
RSSSNWVGKG FFAVYEAICG GDVKKDNGHI QSPNYPDDYR PSKVCIWRIQ VSEGFHVGLT
FQSFEIERHD SCAYDYLEVR DGHSESSNLI GRYCGYEKPD DIKSTSSRLW LKFVSDGSIN
KAGFAVNFFK EVDECSRPNR GGCEQRCLNT LGSYKCSCDP GYELAPDKRR CEAACGGFLT
KLNGSITSPG WPKEYPPNKN CIWQLVAPTQ YRISLQFDFF ETEGNDVCKY DFVEVRSGLT
ADSKLHGKFC GSEKPEVITS QYNNMRVEFK SDNTVSKKGF KAHFFSDKDE CSKDNGGCQQ
DCVNTFGSYE CQCRSGFVLH DNKHDCKEAG CEHKVTSTSG TITSPNWPDK YPSKKECTWA
ISSTPGHRVK LTFVEMDIES QPECAYDHLE VFDGRDAKAP VLGRFCGSKK PEPVLATGNR
MFLRFYSDNS VQRKGFQASH STECGGQVRA DVKTKDLYSH AQFGDNNYPG GVDCEWVIVA
EEGYGVELVF QTFEVEEETD CGYDYMELFD GYDSTAPRLG RYCGSGPPEE VYSAGDSVLV
KFHSDDTISK KGFHLRYTST KFQDTLHSRK

---

tr|D3ZVB7|D3ZVB7_RAT (100 %), 34 071,5 Da
Osteoglycin (Predicted) OS=Rattus norvegicus GN=LOC100910855 PE=4 SV=1
3 exclusive unique peptides, 3 exclusive unique spectra, 4 total spectra, 39/298 amino acids (13 % coverage)

MKTVHPTLLL LLFVPLTQQT PQTQLDSHVN SKYGTDNSEE TKFIQDYEDK YLDGKNIKEK
ETMIIPDEKS LQLQKDEVIP SVPTKKENDE MPTCLLCVCL SGSVYCEEVD IDAVPPLPKE
SAYLYARFNK IKKLTAKDFA DMPNLRRLDF TGNLIEDIED GTFSKLSLLE ELTLAENQLL
RLPVLPPKLT LLNAKYNKIK SKGIKANTFK KLNKLSFLYL DHNDLESVPP NLPESLRVIH
LQFNSISSIT DDTFCKANDT RYIRERMEEI RLEGNPIALG KHPNSFICLK RLPTGSYF

sp|Q5XII0|EPDR1_RAT (100 %), 25 639,5 Da
Mammalian ependymin-related protein 1 OS=Rattus norvegicus GN=Epdr1 PE=2 SV=1
4 exclusive unique peptides, 4 exclusive unique spectra, 5 total spectra, 42/224 amino acids (19 % coverage)

MLTRAPRRLV QGPRETWLLG GLWVWILCGL GMAGSPGTPQ PCQAPQQWEG RQVLYQQSSG
HNSRALVSYD GLNQRVRVLD ERKALIPCKR LFEYILLYKD GVMFQIEQAT KLCAKIPLAE
PWDPLDIPQN STFEDQYSIG GPQEQIMVQE WSDRRTARSY ETWIGVYTAK DCYPVQETFI
RNYTVVLSTR FFDVQLGIKD PSVFTPPSTC QTAQPEKMKE NCSL

sp|Q5FVH0|C1QT5_RAT (100 %), 25 334,7 Da
Complement C1q tumor necrosis factor-related protein 5 OS=Rattus norvegicus GN=C1qtnf5 PE=2 SV=1
3 exclusive unique peptides, 3 exclusive unique spectra, 3 total spectra, 48/243 amino acids (20 % coverage)

MRPLLALLLL GLASGSPPLD DNKIPSLCPG QPGLPGTPGH HGSQGLPGRD GRDGRDGAPG
APGEKGEGGR PGLPGP[illegible] (VLLNEQGHYDATTGK (100 %)) [illegible]GPVG AIGPAGECSV PPRSAFSAKR SESRVPPPAD
TPLPFDRVLL NEQGHYDATT GKFTCQVPGV YYFAVHATVY RASLQFDLVK NGQSIASFFQ
FFGGWPKPAS LSGGAMVRLE PEDQVWVQVG VGDYIGIYAS IKTDSTFSGF LVYSDWHSSP
VFA

sp|Q62718|NTRI_RAT (100 %), 37 997,6 Da
Neurotrimin OS=Rattus norvegicus GN=Ntm PE=1 SV=1
3 exclusive unique peptides, 3 exclusive unique spectra, 5 total spectra, 55/344 amino acids (16 % coverage)

MGVCGYLFLP WKCLVVVSLR LLFLVPTGVP VRSGDATFPK AMDNVTVRQG ESATLRCTID
NRVTRVAWLN RSTILYAGND KWCLDPRVVL LSNTQTQYSI EIQNVDVYDE GPYTCSVQTD
NHPKTSRVHL IVQVSPKIVE ISSDISINEG NNISLTCIAT GRPEPTVTWR HISPKAVGFV
SEDEYLEIQG ITREQSGEYE CSASNDVAAP VVRRVNVTVN YPPYISEAKG TGVPVGQKGT
LQCEASAVPS AEFQWFKDDK RLVEGKKGVK VENRPFLSRL TFFNVSEHDY GNYTCVASNK
LGHTNASIML FGPGAVSEVN NGTSRRAGCI WLLPLLVLHL LLKF

sp|P07936|NEUM_RAT (100 %), 23 603,2 Da
Neuromodulin OS=Rattus norvegicus GN=Gap43 PE=1 SV=1
9 exclusive unique peptides, 10 exclusive unique spectra, 17 total spectra, 143/226 amino acids (63 % coverage)

MLCCMRRTKQ VEKNDEDQKI EQDGVKPEDK AHKAATKIQA SFRGHITRKK LKDEKKGDAP
AAEAEAKEKD DAPVADGVEK KEGDGSATTD AAPATSPKAE EPSKAGDAPS EEKKGEGDAA
PSEEKAGSAE TESAAKATTD NSPSSKAEDG PAKEEPKQAD VPAAVTDAAA TTPAAEDAAK
AAQPPTETAE SSQAEEEKEA VDEAKPKESA RQDEGKEDPE ADQEHA

tr|Q3MID6|Q3MID6_RAT (100 %), 37 148,8 Da
Calumenin OS=Rattus norvegicus GN=Calu PE=2 SV=1
8 exclusive unique peptides, 11 exclusive unique spectra, 22 total spectra, 114/315 amino acids (36 % coverage)

MDLRQFLMCL SLCTAFALSK PTEKKDRVHH EPQLSDKVHN DAQNFDYDHD AFLGAEEAKS
FDQLTPEESK ERLGMIVDKI DTDKDGFVTK GELKSWIKHA QKKYIYDNVE NQWQEFDMNQ
DGLISWDEYR NVTYGTYLDD PDPDDGFNYK QMMVRDERRF KMADKDGDLI ATKEEFTAFL
HPEEYDYMKD IVVQETMEDI DKNADGFIDL EEYIGDMYSH DGNADEPEWV KTEREQFVEF
RDKNRDGKMD KEETKDWILP SDYDHAEAEA RHLVYESDQD KDGKLTKEEI VDKYDLFVGS
QATDFGEALV RHDEF

sp|P97546-3|NPTN_RAT (100 %), 30 836,4 Da
Isoform 3 of Neuroplastin OS=Rattus norvegicus GN=Nptn
2 exclusive unique peptides, 2 exclusive unique spectra, 2 total spectra, 28/277 amino acids (10 % coverage)

MSGSSLPGAL ALSLLLVSGS LLPGPGAAQN EPRIVTSEEV IIRDSLLPVT LQCNLTSSSH
TLMYSYWTKN GVELTATRKN ASNMEYRINK PRAEDSGEYH CVYHFVSAPK ANATIEVKAA
PDITGHKRSE NKNEGQDAMM YCKSVGYPHP EWMWRKKENG VFEEISNSSG RFFIINKENY
TELNIVNLQI TEDPGEYECN ATNSIGSASV STVLRVRSHL APLWPFLGIL AEIIILVVII
VVYEKRKRPD EVPDAGPMKT NSTNNHKDKN LRQRNTN

sp|O35276|NRP2_RAT (100 %), 103 899,5 Da
Neuropilin-2 OS=Rattus norvegicus GN=Nrp2 PE=2 SV=1
2 exclusive unique peptides, 2 exclusive unique spectra, 2 total spectra, 34/925 amino acids (4 % coverage)

MDMFPLTWIF LALYFSGHKV RSQQDPPCGG RLNSKDAGYI TSPGYPQDYP SHQNCEWVVY
APEPNQKIVL NFNPHFEIEK HDCKYDFIEI RDGDSESADL LGKHCGNIAP PTIISSGSVL
YIKFTSDYAR QGAGFSLRYE IFKTGSEDCS KNFTSPNGTI ESPGFPEKYP HNLDCTFTIL
AKPRMEIILQ FLTFDLEHDP LQVGEGDCKY DWLDIWDGIP HVGPLIGKYC GTKTPSKLRS
STGILSLTFH TDMAVAKDGF SARYYLVHQE PPENFQCNAP LGMESGRIAN EQISASSTFS
DGRWTPQQSR LHGDDNGWTP NVDSNKEYLQ VDLRFLTMLT AIATQGAISR ETQKGYYVKS
YKLEVSTNGE DWMVYRHGKN HKVFQANNDA TELVLNKLHT PLLTRFIRIR PQTWHLGIAL
RLELFGCRVT DAPCSNMLGM LSGLIADTQI SASSTREYLW SPSAARLVSS RSGWFPRNPQ
AQPGEEWLQV DLGTPKTVKG VIIQGARGGD SITAMEARAF VRKFKVSYSL NGKDWEYIQD
PRTQQPKLFE GNMHYDTPDI RRFEPVPAQY VRVYPERWSP AGIGMRLEVL GCDWTDSKPT
VETLGPTVKS EETTTPYPMD EDATECGENC SFEDDKDLQL PSGFNCNFDF PEETCGWMYD
RAKWLQSTWI SSANPNDRTF PDDKNFLKLQ SDGGREGQFG RLISPPVHLP RSPVCMEFQY
QAMGGHGVAL QVVREARQES KLLWVIREDQ GSEWKHGRII LPSYDMEYQI VFEGVIGKGR
SGEISIDDIR ISTDVPLENC MEPISAFAVD IPEIHGGEGY EDEIDDDYEG DWNNSSSTSG
AGSPSSGKEK SWLYTLDPIL ITIIAMSSLG VLLGATCAGL LLYCTCSYSG LSSRSCTTLE
NYNFELYDGL KHKVKINHQK CCSEA

sp|Q9QZQ5|NOV_RAT (100 %), 38 508,5 Da
Protein NOV homolog OS=Rattus norvegicus GN=Nov PE=1 SV=1
9 exclusive unique peptides, 9 exclusive unique spectra, 20 total spectra, 104/351 amino acids (30 % coverage)

MSVFLRKQCL CLGFLLLHLL NQVSATLRCP SRCPSQCPSI SPTCAPGVRS VLDGCSCCPV
CARQRGESCS EMRPCDQSSG LYCDRSADPN NETGICMVPE GDNCVFDGVI YRNGEKFEPN
CQYHCTCRDG QIGCVPRCQL DVLLPGPDCP APKKVAVPGE CCEKWTCGSE EKGTLGGLAL
PAYRPEATVG VELSDSSINC IEQTTEWSAC SKSCGMGLST RVTNRNLQCE MVKQTRLCMV
RPCEQEPGEA TDMKGKKCLR TKKSLKSIHL QFKNCTSLYT YKPRFCGICS DGRCCTPFNT
KTIQVEFQCL PGQIIKKPVM VIGTCTCHSN CPQNNEAFLQ ELELKTSRGE M

sp|P30120|TIMP1_RAT (100 %), 23 794,2 Da
Metalloproteinase inhibitor 1 OS=Rattus norvegicus GN=Timp1 PE=1 SV=2
11 exclusive unique peptides, 22 exclusive unique spectra, 49 total spectra, 155/217 amino acids (71 % coverage)

MAPFASLASG ILLLLSLIAS SKACSCAPTH PQTAFCNSDL VIRAKFMGSP EIIETTLYQR YEIKMTKMLK GFDAVGNATG
FRFAYTPAME SLCGYVHKSQ NRSEEFLIAG RLRNGNLHIT ACSFLVPWHN LSPAQQKAFV KTYSAGCGVC TVFPCSAIPC
KLESDSHCLW TDQILMGSEK GYQSDHFACL PRNPDLCTWQ YLGVSMTRSL PLAKAEA

sp|P30121|TIMP2_RAT (100 %), 24 357,0 Da
Metalloproteinase inhibitor 2 OS=Rattus norvegicus GN=Timp2 PE=1 SV=3
11 exclusive unique peptides, 15 exclusive unique spectra, 33 total spectra, 119/220 amino acids (54 % coverage)

MGAAARSLRL ALGLLLLATL LRPADACSCS PVHPQQAFCN ADVVIRAKAV SEKEVDSGND IYGNPIKRIQ YEIKQIKMFK
GPDKDIEFIY TAPSSAVCGV SLDVGGKKEY LIAGKAEGDG KMHITLCDFI VPWDTLSITQ KKSLNHRYQM GCECKITRCP
MIPCYISSPD ECLWMDWVTE KSINGHQAKF FACIKRSDGS CAWYRGAAPP KQEFLDIEDP

tr|D3ZVB7|D3ZVB7_RAT (100 %), 34 071,5 Da
Osteoglycin (Predicted) OS=Rattus norvegicus GN=LOC100910855 PE=4 SV=1
3 exclusive unique peptides, 3 exclusive unique spectra, 4 total spectra, 39/298 amino acids (13 % coverage)

MKTVHPTLLL LLFVPLTQQT PQTQLDSHVN SKYGTDNSEE TKFIQDYEDK YLDGKNIKEK
ETMIIPDEKS LQLQKDEVIP SVPTKKENDE MPTCLLCVCL SGSVYCEEVD IDAVPPLPKE
SAYLYARFNK IKKLTAKDFA DMPNLRRLDF TGNLIEDIED GTFSKLSLLE ELTLAENQLL
RLPVLPPKLT LLNAKYNKIK SKGIKANTFK KLNKLSFLYL DHNDLESVPP NLPESLRVIH
LQFNSISSIT DDTFCKANDT RYIRERMEEI RLEGNPIALG KHPNSFICLK RLPTGSYF

tr|D3ZAF5|D3ZAF5_RAT (100 %), 90 059,4 Da
Periostin, osteoblast specific factor (Predicted), isoform CRA_a OS=Rattus norvegicus GN=Postn PE=4 SV=1
39 exclusive unique peptides, 56 exclusive unique spectra, 240 total spectra, 534/810 amino acids (66 % coverage)

```
MVPLLPLSAL  LLLFLCDVDP  ANANSYYDKV  LAHSRIRGRD  QGPNVCALQQ  ILGTKKKYFS
SCKNWYQGAI  CGKKTTVLYE  CCPGYMRMEG  MKGCPAVMPI  DHVYGTLGIV  GATTTQHYSD
VSKLREEIEG  KGSYTYFAPS  NEAWDNLDSD  IRRGLENNVN  VELLNALHSH  MVNKRMLTKD
LKHGMVIPSM  YNNLGLFINH  YPNGVVTVNC  ARVIHGNQIA  TNGVVHVIDR  VLTQIGTSIQ
DFIEAEDELS  SFRAAAITSD  LLESLGRDGH  FTLFAPTNEA  FEKLPRGVLE  RIMGDKVASE
ALMKYHILNT  LQCSEAITGG  AVFETMEGNT  IEIGCEGDSI  SINGIKMVNK  KDIVTKNGVI
HLIDEVLIPD  SAKQVIELAG  KQQTTFTDLV  AQLGLASSLK  PDGEYTLLAP  VNNAFSDDTL
SMDQRLLKLI  LQNHILKVKV  GLSDL[illegible]  [illegible]  [illegible]FVYRTAICI  ENSCMVRGSK
QGRNGAIHIF  REIIQPAEKS  LHEKL[illegible]  [illegible]  [illegible]DLKDLLTQP  GDWTLFAPTN
DAFKGMTNEE  REILIGDKNA  LQNIILYHLT  PGVYIGKGFE  PGVTNILKTT  QGSKIYVKGV
NETLLVNELK  SKESDIMTTN  GVIHVVDKLL  YPADIPVGND  QLLELLNKLI  KYIQIKFVRG
STFKEIPMTV  YTTKIITKLV  EPKIKVIQGS  LQPIIKTEGP  AMTKIHIEGE  PDFRLIKEGE
TVTEVIHGEP  VIKKYTKIID  GVPVEITEKE  TREERIITGP  EIKYTRISTG  GGETEETLQK
FLQKDTPAKK  IQANKRVQGS  RRRSREGRSQ
```

NALQNIILYHLTPGVYIGK (100 %) x18
EILIGDKNALQNIILYHLTPGVYIGK (100 %) x4

---

sp|P08721|OSTP_RAT (100 %), 34 962,7 Da
Osteopontin OS=Rattus norvegicus GN=Spp1 PE=1 SV=2
4 exclusive unique peptides, 4 exclusive unique spectra, 5 total spectra, 58/317 amino acids (18 % coverage)

```
MRLAVVCFCL  FGLASCLPVK  VAEFGSSEEK  AHYSKHSDAV  ATWLKPDPSQ  KQNLLAPQNS
VSSEETDDFK  QETLPSNSNE  SHDHMDDDDD  DDDDGDHAES  EDSVNSDESD  ESHHSDESDE
SFTASTQADV  LTPIAPTVDV  PDGRGDSLAY  GLRSKSRSFP  VSDEQYPDAT  DEDLTSRMKS
QESDEAIKVI  PVAQRLSVPS  DQDSNGKTSH  ESSQLDEPSV  ETHSLEQSKE  YKQRASHEST
EQSDAIDSAE  KPDAIDSAER  SDAIDSQASS  KASLEHQSHE  FHSHEDKLVL  DPKSKEDDRY
LKFRISHELE  SSSSEVN
```

sp|P16975|SPRC_RAT (100 %), 34 295,7 Da
SPARC OS=Rattus norvegicus GN=Sparc PE=1 SV=4
16 exclusive unique peptides, 25 exclusive unique spectra, 155 total spectra, 181/301 amino acids (60 % coverage)

```
MRAWIFFLLC  LAGRALAAPQ  TEAAEEMVAE  ETVVEETGLP  VGANPVQVEM  GEFEEGAEET
VEEVVAENPC  QNHHCKHGKV  CELDESNTPM  CVCQDPTSCP  APIGEFEKVC  SNDNKTFDSS
CHFFATKCTL  EGTKKGHKLH  LDYIGPCKYI  APCLDSELTE  FPLRMRDWLK  NVLVTLYERD
EGNNLLTEKQ  KLRVKKIHEN  EKRLEAGDHP  VELLARDFEK  NYNMYIFPVH  WQFGQLDQHP
IDGYLSHTEL  APLRAPLIPM  EHCTTRFFET  CDLDNDKYIA  LEEWAGCFGI  KEQDINKDLV
I
```

sp|Q810F4|FAM3C_RAT (100 %), 24 713,9 Da
Protein FAM3C OS=Rattus norvegicus GN=Fam3c PE=2 SV=1
2 exclusive unique peptides, 2 exclusive unique spectra, 2 total spectra, 20/227 amino acids (9 % coverage)

```
MRVAGAAKLV  VAVAVFLLTF  YVISQVFEIK  MDASLGSLFA  RSALDSAIRS  TKPPRYKCGI
SKACPEKHFA  FKMASGAANV  VGPKICLEDN  VLMSGVKNNV  GRGINVALVN  GKTGDVIDTK
YFDMWGGDVA  PFIEFLKTIQ  DGTVVLMATY  DDGATKLTEE  ARRLIAELGS  TSITSLGFRD
NWVFCGGKGI  KTKSPFEQHI  KNNKDTNKYE  GWPEVVEMEG  CIPQKQD
```

sp|Q63532|SPR1A_RAT (100 %), 16 731,5 Da
Cornifin-A OS=Rattus norvegicus GN=Sprr1a PE=2 SV=1
2 exclusive unique peptides, 2 exclusive unique spectra, 3 total spectra, 16/152 amino acids (11 % coverage)

```
MSSQQQKQPC  TVPPQLHQHE  VKQPCQPPPQ  EPCAPKTKEP  CHPIPEPCNP  KVPEPCQPKV
PEPCQPKVPE  PCQPKVPEPC  QPKVPEPCQP  KVPEPCQPKV  PEPCHPKAPE  PCHPVVPEPC
QPVAPEPCQP  VVPEPCPPTV  TPSPYQQKTK  QK
```

Modulation properties of bone marrow stromal cells released factors on activated microglia: in vitro study

Dasa Cizkova1,2*, Stéphanie Devaux1*, Françoise Le Marrec-Croq1, Julien Franck1,Lucia Slovinska2,Juraj Blasko2, Jan Rosocha3,Timea Spakova3, Christophe Lefebvre1, Isabelle Fournier1, Michel Salzet1

Supplementary data 3

Table. List of microglia morphological characteristics following CM treatment

The morphological changes of BV2 cells and PM was defined by five following parameters: soma diameter, soma area, process diameter and length, and process length in relation to the soma diameter. Data were collected from measurements of 100 cells per CM treatment.

CM	Microglia type	Soma diameter (µm)	Soma area (µm)	Process Diameter (µm)	Process Length (µm)	Process Length/ Soma Diameter
DMEM	BV2 cells	12.3-15.9	177.18 ± 98	<1-none	< 7	
	PM	4,8-6.18	71.56 ± 15	<1	40.2-50.6	> 10x
SC-CM	BV2Cells	14.7-21.3	408.21 ± 126.3	<1-none	< 5	
	PM	6.9-7.18	78.95 ± 10	<2	35.1-48.7	> 6x
SCI-CM	BV2 cells	18.6-44,5	668.87 ± 200.7	2.1-4.2	40.3-53.8	> 2x
	PM	24.2-28.1	745.84 ± 115.3	1.3-4.9	23.4-35.6	< 1x
SCI-CM-BMSCs	BV2 cells	20.5-25.6	709.21 ± 108	<2	10.3-13.5	< 1x
	PM	14.5-17.8	214.18 ± 104	<3	23.4-30.8	> 1x

marrow stromal cells released factors on activated microglia: in vitro study

Dasa Cizkova1,2*, Stéphanie Devaux1*, Françoise Le Marrec-Croq1, Julien Franck1,Lucia Slovinska2,J

Experiment: msc
Peak List Generator: unknown
Version: unknown
Charge States Calculated: True
Deisotoped: True
Textual Annotation: unknown
Database Set: 2 Databases
Database Name: Rattus_norvegicus_Unipr
Version: unknown
Taxonomy: All Entries
Number of Proteins: 35683
Database Name: a subset of the RAT datab
Version: unknown
Taxonomy: All Entries
Number of Proteins: 28298
Does database contain common contamin
Search Engine Set: 2 Search Engines
Search Engine: Sequest
Version: 1.3.0.339
Samples: All Samples
Fragment Tolerance: 0,50 Da
Parent Tolerance: 10,0 PPM
Fixed Modifications: +57 on
Variable Modifications: +16
Database: Rattus_norvegicu
Digestion Enzyme: Trypsin
Max Missed Cleavages: 3
Probability Model:
20130328-MS
091012_CTL-0
091012_L-02:
Search Engine: X! Tandem
Version: CYCLONE (2010.12.
Samples: All Samples
Fragment Tolerance: 0,50 Da
Parent Tolerance: 10,0 PPM
Fixed Modifications: +57 on
Variable Modifications: -18 c
Database: a subset of the RA
Digestion Enzyme: Trypsin
Max Missed Cleavages: 2
Probability Model:
20130328-MS
091012_CTL-0
091012_L-02:
Scaffold: Version: Scaffold_4.2.1
Modification Metadata Set: 1541 modifica
Source: C:\Program Files\Sca
Comment:
Protein Grouping Strategy: Experiment-wi

Peptide Thresholds: 97,0 % minimum
Protein Thresholds: 99,0 % minimum and 2
Peptide FDR: 0,1 % (Decoy)
Protein FDR: 1,0 % (Decoy)
GO Annotation Source(s):

	Biological sam	Biological sam	MS/MS sampl	Protein name
Experiment name	MSC	MSC	20130328-MS	Isoform 2 of T
msc	MSC	MSC	20130328-MS	Transgelin-2 C
msc	MSC	MSC	20130328-MS	Protein Ube2l
msc	MSC	MSC	20130328-MS	V-type proton
msc	MSC	MSC	20130328-MS	Annexin A2 OS
msc	MSC	MSC	20130328-MS	C-type lectin c
msc	MSC	MSC	20130328-MS	Protein Map2
msc	MSC	MSC	20130328-MS	Fibulin 2, isofo
msc	MSC	MSC	20130328-MS	Keratin, type I
msc	MSC	MSC	20130328-MS	Latent-transfo
msc	MSC	MSC	20130328-MS	NADP-depenc
msc	MSC	MSC	20130328-MS	Glypican 4 OS
msc	MSC	MSC	20130328-MS	Peroxiredoxin
msc	MSC	MSC	20130328-MS	Macrophage-c
msc	MSC	MSC	20130328-MS	Isoform 2 of A
msc	MSC	MSC	20130328-MS	Kallikrein 6, is
msc	MSC	MSC	20130328-MS	L-lactate dehy
msc	MSC	MSC	20130328-MS	Fibulin 1 (Prec
msc	MSC	MSC	20130328-MS	Collagen alpha
msc	MSC	MSC	20130328-MS	Protein Thbs2
msc	MSC	MSC	20130328-MS	Calsyntenin-1
msc	MSC	MSC	20130328-MS	Protein FAM3
msc	MSC	MSC	20130328-MS	Protein Hbb-b
msc	MSC	MSC	20130328-MS	Calumenin OS
msc	MSC	MSC	20130328-MS	Protein Lamc1
msc	MSC	MSC	20130328-MS	Isoform 3 of T
msc	MSC	MSC	20130328-MS	Histone H2A t
msc	MSC	MSC	20130328-MS	Collagen alpha
msc	MSC	MSC	20130328-MS	Kinesin-1 heav
msc	MSC	MSC	20130328-MS	Tubulin alpha-
msc	MSC	MSC	20130328-MS	Spectrin alpha
msc	MSC	MSC	20130328-MS	RCG32401, iso
msc	MSC	MSC	20130328-MS	Protein Hepac
msc	MSC	MSC	20130328-MS	Translationally
msc	MSC	MSC	20130328-MS	Kininogen 1 O
msc	MSC	MSC	20130328-MS	Follistatin-rela
msc	MSC	MSC	20130328-MS	Collagen alpha
msc	MSC	MSC	20130328-MS	Protein IMPA(
msc	MSC	MSC	20130328-MS	Protein Lamb1
msc	MSC	MSC	20130328-MS	Lymphocyte c
msc	MSC	MSC	20130328-MS	Annexin (Frag
msc	MSC	MSC	20130328-MS	Decorin OS=R
msc	MSC	MSC	20130328-MS	Destrin OS=Ra
msc	MSC	MSC	20130328-MS	Alpha 4 type V

msc	MSC	MSC	20130328-MS	Protein Cfh OS
msc	MSC	MSC	20130328-MS	Isoform 3 of D
msc	MSC	MSC	20130328-MS	Procollagen-ly
msc	MSC	MSC	20130328-MS	Protein Serpin
msc	MSC	MSC	20130328-MS	Type II keratin
msc	MSC	MSC	20130328-MS	Apolipoprotei
msc	MSC	MSC	20130328-MS	Isoform V3 of
msc	MSC	MSC	20130328-MS	14-3-3 protein
msc	MSC	MSC	20130328-MS	Beta-2-microg
msc	MSC	MSC	20130328-MS	Legumain OS=
msc	MSC	MSC	20130328-MS	Actin, aortic s
msc	MSC	MSC	20130328-MS	Protein Col8a
msc	MSC	MSC	20130328-MS	Uncharacteriz
msc	MSC	MSC	20130328-MS	Phosphatidyle
msc	MSC	MSC	20130328-MS	Protein Npep
msc	MSC	MSC	20130328-MS	Peripherin OS
msc	MSC	MSC	20130328-MS	Protein disulfi
msc	MSC	MSC	20130328-MS	Fibulin-5 OS=
msc	MSC	MSC	20130328-MS	Mammalian e
msc	MSC	MSC	20130328-MS	Inter-alpha-tr
msc	MSC	MSC	20130328-MS	SPARC OS=Ra
msc	MSC	MSC	20130328-MS	Gremlin-1 OS=
msc	MSC	MSC	20130328-MS	Inositol mono
msc	MSC	MSC	20130328-MS	Heat shock pr
msc	MSC	MSC	20130328-MS	Plasminogen a
msc	MSC	MSC	20130328-MS	Metalloprotei
msc	MSC	MSC	20130328-MS	Protein Krt35
msc	MSC	MSC	20130328-MS	Aggrecan core
msc	MSC	MSC	20130328-MS	Sushi-repeat-
msc	MSC	MSC	20130328-MS	Glycogen pho
msc	MSC	MSC	20130328-MS	Osteopontin (
msc	MSC	MSC	20130328-MS	Uncharacteriz
msc	MSC	MSC	20130328-MS	Plastin 3 (T-iso
msc	MSC	MSC	20130328-MS	Histone H3 OS
msc	MSC	MSC	20130328-MS	Adenylyl cycla
msc	MSC	MSC	20130328-MS	Isoform Tau-B
msc	MSC	MSC	20130328-MS	Afamin OS=Ra
msc	MSC	MSC	20130328-MS	Gamma-synuc
msc	MSC	MSC	20130328-MS	Lysozyme C-1
msc	MSC	MSC	20130328-MS	Hemoglobin s
msc	MSC	MSC	20130328-MS	LIM and SH3 d
msc	MSC	MSC	20130328-MS	Procollagen, t
msc	MSC	MSC	20130328-MS	Glutamate de
msc	MSC	MSC	20130328-MS	Transcobalam
msc	MSC	MSC	20130328-MS	Lactoylglutath
msc	MSC	MSC	20130328-MS	ATP synthase
msc	MSC	MSC	20130328-MS	Alpha-2-macr
msc	MSC	MSC	20130328-MS	Transcriptiona
msc	MSC	MSC	20130328-MS	Adenine phos
msc	MSC	MSC	20130328-MS	Dihydropyrim
msc	MSC	MSC	20130328-MS	Bone morpho

msc	MSC	MSC	20130328-MS	Calmodulin OS
msc	MSC	MSC	20130328-MS	Alpha-1-antip
msc	MSC	MSC	20130328-MS	Protein Col4a1
msc	MSC	MSC	20130328-MS	Actin, cytoplas
msc	MSC	MSC	20130328-MS	Pyruvate kinas
msc	MSC	MSC	20130328-MS	Histone H2B t
msc	MSC	MSC	20130328-MS	Reticulon-4 OS
msc	MSC	MSC	20130328-MS	Brevican core
msc	MSC	MSC	20130328-MS	Insulin-like gro
msc	MSC	MSC	20130328-MS	Rabphilin-3A (
msc	MSC	MSC	20130328-MS	Vimentin OS=
msc	MSC	MSC	20130328-MS	Uncharacteriz
msc	MSC	MSC	20130328-MS	Heat shock 70
msc	MSC	MSC	20130328-MS	Inter-alpha try
msc	MSC	MSC	20130328-MS	Myelin-associa
msc	MSC	MSC	20130328-MS	Protein-lysine
msc	MSC	MSC	20130328-MS	Protein Hspg2
msc	MSC	MSC	20130328-MS	Apolipoprotei
msc	MSC	MSC	20130328-MS	Procollagen, t
msc	MSC	MSC	20130328-MS	Protein Txndc
msc	MSC	MSC	20130328-MS	Collagen alpha
msc	MSC	MSC	20130328-MS	Alpha-enolase
msc	MSC	MSC	20130328-MS	Protein Ncam
msc	MSC	MSC	20130328-MS	Isoform TGF-b
msc	MSC	MSC	20130328-MS	Isoform 2 of G
msc	MSC	MSC	20130328-MS	ATP synthase
msc	MSC	MSC	20130328-MS	Polyubiquitin-
msc	MSC	MSC	20130328-MS	Murinoglobuli
msc	MSC	MSC	20130328-MS	Serine proteas
msc	MSC	MSC	20130328-MS	Keratin, type I
msc	MSC	MSC	20130328-MS	Glypican-1 OS
msc	MSC	MSC	20130328-MS	Serine (Or cys
msc	MSC	MSC	20130328-MS	Isoform 1 of C
msc	MSC	MSC	20130328-MS	Heat shock pr
msc	MSC	MSC	20130328-MS	Hexokinase-1
msc	MSC	MSC	20130328-MS	Junction plakc
msc	MSC	MSC	20130328-MS	Protein Ank2 (
msc	MSC	MSC	20130328-MS	Vinculin OS=R
msc	MSC	MSC	20130328-MS	Clathrin heavy
msc	MSC	MSC	20130328-MS	NAD(P)H dehy
msc	MSC	MSC	20130328-MS	Mast cell carb
msc	MSC	MSC	20130328-MS	Protein FAM1
msc	MSC	MSC	20130328-MS	Lysyl oxidase-
msc	MSC	MSC	20130328-MS	Keratin, type I
msc	MSC	MSC	20130328-MS	Filamin alpha
msc	MSC	MSC	20130328-MS	Isoform 2 of S
msc	MSC	MSC	20130328-MS	Follistatin-rela
msc	MSC	MSC	20130328-MS	Monocyte diff
msc	MSC	MSC	20130328-MS	Glutamine syr
msc	MSC	MSC	20130328-MS	Cathepsin D C
msc	MSC	MSC	20130328-MS	Keratin, type I

msc	MSC	MSC	20130328-MS	Alpha-actinin-
msc	MSC	MSC	20130328-MS	Serine proteas
msc	MSC	MSC	20130328-MS	Nucleoside di
msc	MSC	MSC	20130328-MS	Uncharacteriz
msc	MSC	MSC	20130328-MS	Protein Itih2 (
msc	MSC	MSC	20130328-MS	Uncharacteriz
msc	MSC	MSC	20130328-MS	Complement (
msc	MSC	MSC	20130328-MS	Procollagen C-
msc	MSC	MSC	20130328-MS	Adenylate kin
msc	MSC	MSC	20130328-MS	Protein Psat1
msc	MSC	MSC	20130328-MS	Superoxide di
msc	MSC	MSC	20130328-MS	Collagen alpha
msc	MSC	MSC	20130328-MS	Uncharacteriz
msc	MSC	MSC	20130328-MS	Protein Itih4 (
msc	MSC	MSC	20130328-MS	Protein Zc3h1
msc	MSC	MSC	20130328-MS	Peptidyl-proly
msc	MSC	MSC	20130328-MS	60S acidic ribo
msc	MSC	MSC	20130328-MS	Aspartate ami
msc	MSC	MSC	20130328-MS	Fibronectin OS
msc	MSC	MSC	20130328-MS	Uncharacteriz
msc	MSC	MSC	20130328-MS	6-phosphoglu
msc	MSC	MSC	20130328-MS	Transforming
msc	MSC	MSC	20130328-MS	Apolipoprotei
msc	MSC	MSC	20130328-MS	Protein LOC10
msc	MSC	MSC	20130328-MS	Peroxiredoxin
msc	MSC	MSC	20130328-MS	Tubulin beta-3
msc	MSC	MSC	20130328-MS	Complement (
msc	MSC	MSC	20130328-MS	Growth arrest
msc	MSC	MSC	20130328-MS	Fibrinogen be
msc	MSC	MSC	20130328-MS	Histone H4 OS
msc	MSC	MSC	20130328-MS	Osteoglycin (F
msc	MSC	MSC	20130328-MS	Protein disulfi
msc	MSC	MSC	20130328-MS	Isoform Gamr
msc	MSC	MSC	20130328-MS	Nucleobindin-
msc	MSC	MSC	20130328-MS	Plasminogen (
msc	MSC	MSC	20130328-MS	Sulfated glyco
msc	MSC	MSC	20130328-MS	Peptidyl-proly
msc	MSC	MSC	20130328-MS	Platelet-derive
msc	MSC	MSC	20130328-MS	Keratin, type I
msc	MSC	MSC	20130328-MS	Protein Dkk3 (
msc	MSC	MSC	20130328-MS	Connective tis
msc	MSC	MSC	20130328-MS	Isoform Crk-I (
msc	MSC	MSC	20130328-MS	Phosphoglyce
msc	MSC	MSC	20130328-MS	Microtubule-a
msc	MSC	MSC	20130328-MS	Secernin-1 OS
msc	MSC	MSC	20130328-MS	Complement f
msc	MSC	MSC	20130328-MS	Alcohol dehyd
msc	MSC	MSC	20130328-MS	CArG-binding
msc	MSC	MSC	20130328-MS	Neurofilamen
msc	MSC	MSC	20130328-MS	Keratin, type I
msc	MSC	MSC	20130328-MS	14-3-3 protein

msc	MSC	MSC	20130328-MS	Uncharacteriz
msc	MSC	MSC	20130328-MS	Nidogen-1 OS
msc	MSC	MSC	20130328-MS	72 kDa type I\
msc	MSC	MSC	20130328-MS	Protein Dsp O
msc	MSC	MSC	20130328-MS	Spondin-2 OS=
msc	MSC	MSC	20130328-MS	Neurofilamen
msc	MSC	MSC	20130328-MS	Neural cell ad
msc	MSC	MSC	20130328-MS	Glucose-6-pho
msc	MSC	MSC	20130328-MS	Uncharacteriz
msc	MSC	MSC	20130328-MS	Prelamin-A/C
msc	MSC	MSC	20130328-MS	Isoform 3 of N
msc	MSC	MSC	20130328-MS	Dipeptidyl pe
msc	MSC	MSC	20130328-MS	Uncharacteriz
msc	MSC	MSC	20130328-MS	Procollagen, t
msc	MSC	MSC	20130328-MS	Cofilin-1 OS=R
msc	MSC	MSC	20130328-MS	Isoform IB of S
msc	MSC	MSC	20130328-MS	Tubulin alpha-
msc	MSC	MSC	20130328-MS	Complement c
msc	MSC	MSC	20130328-MS	Isoform 2 of N
msc	MSC	MSC	20130328-MS	Carbonic anhy
msc	MSC	MSC	20130328-MS	Neuropilin-2 O
msc	MSC	MSC	20130328-MS	Keratin, type I
msc	MSC	MSC	20130328-MS	Alpha-1-macro
msc	MSC	MSC	20130328-MS	Ubiquitin carb
msc	MSC	MSC	20130328-MS	Glial fibrillary
msc	MSC	MSC	20130328-MS	Rab GDP disso
msc	MSC	MSC	20130328-MS	Phosphorylase
msc	MSC	MSC	20130328-MS	14-3-3 protein
msc	MSC	MSC	20130328-MS	Transforming
msc	MSC	MSC	20130328-MS	Creatine kinas
msc	MSC	MSC	20130328-MS	Protein Sema7
msc	MSC	MSC	20130328-MS	Adipocyte enh
msc	MSC	MSC	20130328-MS	Superoxide di
msc	MSC	MSC	20130328-MS	Uncharacteriz
msc	MSC	MSC	20130328-MS	Hyaluronan ar
msc	MSC	MSC	20130328-MS	Glutathione S-
msc	MSC	MSC	20130328-MS	Glutamate [NI
msc	MSC	MSC	20130328-MS	Serum albumi
msc	MSC	MSC	20130328-MS	Epididymal se
msc	MSC	MSC	20130328-MS	Rho GDP-disso
msc	MSC	MSC	20130328-MS	Peptidyl-proly
msc	MSC	MSC	20130328-MS	A disintegrin a
msc	MSC	MSC	20130328-MS	Cathepsin B O
msc	MSC	MSC	20130328-MS	Procollagen-ly
msc	MSC	MSC	20130328-MS	Latent transfo
msc	MSC	MSC	20130328-MS	Microtubule-a
msc	MSC	MSC	20130328-MS	Thioredoxin O
msc	MSC	MSC	20130328-MS	WD repeat-co
msc	MSC	MSC	20130328-MS	Myelin protec
msc	MSC	MSC	20130328-MS	Malate dehyd
msc	MSC	MSC	20130328-MS	C1qtnf3 prote

msc	MSC	MSC	20130328-MS	Protein Krt32
msc	MSC	MSC	20130328-MS	Glyceraldehyc
msc	MSC	MSC	20130328-MS	Alpha-centrac
msc	MSC	MSC	20130328-MS	Protein SET O:
msc	MSC	MSC	20130328-MS	Transthyretin
msc	MSC	MSC	20130328-MS	Vitamin D-bin
msc	MSC	MSC	20130328-MS	Uncharacteriz
msc	MSC	MSC	20130328-MS	Synaptosomal
msc	MSC	MSC	20130328-MS	Latexin OS=Ra
msc	MSC	MSC	20130328-MS	Apolipoprotei
msc	MSC	MSC	20130328-MS	Uncharacteriz
msc	MSC	MSC	20130328-MS	Keratin, type I
msc	MSC	MSC	20130328-MS	Sodium/potas
msc	MSC	MSC	20130328-MS	Isoform B of A
msc	MSC	MSC	20130328-MS	Keratin, type I
msc	MSC	MSC	20130328-MS	Neuronal cell
msc	MSC	MSC	20130328-MS	Protein Sh3bg
msc	MSC	MSC	20130328-MS	Peroxiredoxin
msc	MSC	MSC	20130328-MS	Dihydropyrim
msc	MSC	MSC	20130328-MS	Protein AMBP
msc	MSC	MSC	20130328-MS	Chaperonin co
msc	MSC	MSC	20130328-MS	Cysteine and g
msc	MSC	MSC	20130328-MS	Protein S100a
msc	MSC	MSC	20130328-MS	Hemopexin O:
msc	MSC	MSC	20130328-MS	Placenta grow
msc	MSC	MSC	20130328-MS	Hyaluronan ar
msc	MSC	MSC	20130328-MS	Microtubule-a
msc	MSC	MSC	20130328-MS	Lactadherin O
msc	MSC	MSC	20130328-MS	Complement (
msc	MSC	MSC	20130328-MS	Biglycan OS=R
msc	MSC	MSC	20130328-MS	Protein Lama4
msc	MSC	MSC	20130328-MS	Alpha-1-inhibi
msc	MSC	MSC	20130328-MS	Disintegrin an
msc	MSC	MSC	20130328-MS	Profilin-2 OS=
msc	MSC	MSC	20130328-MS	Tubulin beta-2
msc	MSC	MSC	20130328-MS	Vesicle-fusing
msc	MSC	MSC	20130328-MS	Keratin, type I
msc	MSC	MSC	20130328-MS	Heat shock co
msc	MSC	MSC	20130328-MS	Calretinin OS=
msc	MSC	MSC	20130328-MS	Acylphosphata
msc	MSC	MSC	20130328-MS	Cathepsin L1 (
msc	MSC	MSC	20130328-MS	Protein Krt86
msc	MSC	MSC	20130328-MS	L-lactate dehy
msc	MSC	MSC	20130328-MS	Collagen alpha
msc	MSC	MSC	20130328-MS	Fructose-bispl
msc	MSC	MSC	20130328-MS	Lumican OS=R
msc	MSC	MSC	20130328-MS	Moesin (Fragr
msc	MSC	MSC	20130328-MS	Protein Col6a1
msc	MSC	MSC	20130328-MS	Plakophilin 1 (
msc	MSC	MSC	20130328-MS	Hydroxyacyl g
msc	MSC	MSC	20130328-MS	Haptoglobin C

msc	MSC	MSC	20130328-MS	Fibronectin O:
msc	MSC	MSC	20130328-MS	Collagen alpha
msc	MSC	MSC	20130328-MS	Acylphosphata
msc	MSC	MSC	20130328-MS	Fibrinogen alp
msc	MSC	MSC	20130328-MS	Glutathione S-
msc	MSC	MSC	20130328-MS	Uncharacteriz
msc	MSC	MSC	20130328-MS	Nucleobindin-
msc	MSC	MSC	20130328-MS	Matrix Gla pro
msc	MSC	MSC	20130328-MS	Transitional e
msc	MSC	MSC	20130328-MS	14-3-3 protein
msc	MSC	MSC	20130328-MS	Actin-related
msc	MSC	MSC	20130328-MS	Protein LOC10
msc	MSC	MSC	20130328-MS	Protein LOC10
msc	MSC	MSC	20130328-MS	Beta-galactosi
msc	MSC	MSC	20130328-MS	Nucleoside dip
msc	MSC	MSC	20130328-MS	Keratin, type I
msc	MSC	MSC	20130328-MS	Ectonucleotid
msc	MSC	MSC	20130328-MS	Isoform 2 of P
msc	MSC	MSC	20130328-MS	Ubiquitin thio
msc	MSC	MSC	20130328-MS	Alpha-2-HS-gl
msc	MSC	MSC	20130328-MS	Malate dehyd
msc	MSC	MSC	20130328-MS	Cornifin-A OS=
msc	MSC	MSC	20130328-MS	Acyl-CoA-bind
msc	MSC	MSC	20130328-MS	Protein CutA (
msc	MSC	MSC	20130328-MS	Extracellular s
msc	MSC	MSC	20130328-MS	Protein Serpin
msc	MSC	MSC	20130328-MS	Protein Krt31
msc	MSC	MSC	20130328-MS	Uncharacteriz
msc	MSC	MSC	20130328-MS	Uncharacteriz
msc	MSC	MSC	20130328-MS	Thy-1 membra
msc	MSC	MSC	20130328-MS	Olfactomedin-
msc	MSC	MSC	20130328-MS	Inhibin beta A
msc	MSC	MSC	20130328-MS	Thioredoxin-li
msc	MSC	MSC	20130328-MS	Serine proteas
msc	MSC	MSC	20130328-MS	Guanine deam
msc	MSC	MSC	20130328-MS	Isoform 3 of N
msc	MSC	MSC	20130328-MS	Calpain small
msc	MSC	MSC	20130328-MS	Heat shock 70
msc	MSC	MSC	20130328-MS	Annexin A5 O:
msc	MSC	MSC	20130328-MS	Clusterin OS=I
msc	MSC	MSC	20130328-MS	Uncharacteriz
msc	MSC	MSC	20130328-MS	Aldose reduct
msc	MSC	MSC	20130328-MS	Tubulin polym
msc	MSC	MSC	20130328-MS	Annexin A1 O:
msc	MSC	MSC	20130328-MS	Anionic trypsi
msc	MSC	MSC	20130328-MS	2',3'-cyclic-nu
msc	MSC	MSC	20130328-MS	Granulins OS=
msc	MSC	MSC	20130328-MS	Protein Col6a:
msc	MSC	MSC	20130328-MS	Uncharacteriz
msc	MSC	MSC	20130328-MS	Triosephosph
msc	MSC	MSC	20130328-MS	Profilin-1 OS=

msc	MSC	MSC	20130328-MS	Isoform B of In
msc	MSC	MSC	20130328-MS	Neuromodulin
msc	MSC	MSC	20130328-MS	Transketolase
msc	MSC	MSC	20130328-MS	Elongation fac
msc	MSC	MSC	20130328-MS	Serotransferri
msc	MSC	MSC	20130328-MS	Protein Krt76
msc	MSC	MSC	20130328-MS	Acid ceramida
msc	MSC	MSC	20130328-MS	Uncharacteriz
msc	MSC	MSC	20130328-MS	Neurofilamen
msc	MSC	MSC	20130328-MS	Cystatin-C OS=
msc	MSC	MSC	20130328-MS	FK506 binding
msc	MSC	MSC	20130328-MS	Galectin-1 OS=
msc	MSC	MSC	20130328-MS	Protein Flnc O
msc	MSC	MSC	20130328-MS	Matrix-remod
msc	MSC	MSC	20130328-MS	14-3-3 protein
msc	MSC	MSC	20130328-MS	Fibronectin ty
msc	MSC	MSC	20130328-MS	Uncharacteriz
msc	MSC	MSC	20130328-MS	Dynein light c
msc	MSC	MSC	20130328-MS	Aconitate hyd
msc	MSC	MSC	20130328-MS	Calcium/calm
msc	MSC	MSC	20130328-MS	6-phosphoglu
msc	MSC	MSC	20130328-MS	Elongation fac
msc	MSC	MSC	20130328-MS	Uncharacteriz
msc	MSC	MSC	20130328-MS	Uncharacteriz
msc	MSC	MSC	20130328-MS	Protein Col6a
msc	MSC	MSC	20130328-MS	4-trimethylam
msc	MSC	MSC	20130328-MS	Uncharacteriz
msc	MSC	MSC	20130328-MS	Serpin B5 OS=
msc	MSC	MSC	20130328-MS	Uncharacteriz
msc	MSC	MSC	20130328-MS	Ig gamma-2A
msc	MSC	MSC	20130328-MS	HtrA serine pe
msc	MSC	MSC	20130328-MS	Protein NOV h
msc	MSC	MSC	20130328-MS	Protein LOC67
msc	MSC	MSC	20130328-MS	Cytochrome c
msc	MSC	MSC	20130328-MS	Ubiquitin carb
msc	MSC	MSC	20130328-MS	Coiled-coil do
msc	MSC	MSC	20130328-MS	Purine nucleo
msc	MSC	MSC	20130328-MS	Protein Col4a
msc	MSC	MSC	20130328-MS	Peroxiredoxin
msc	MSC	MSC	20130328-MS	Macrophage r
msc	MSC	MSC	20130328-MS	Alpha-actinin-
msc	MSC	MSC	20130328-MS	2,3-bisphosph
msc	MSC	MSC	20130328-MS	F-actin-cappir
msc	MSC	MSC	20130328-MS	Tubulin beta-2
msc	MSC	MSC	20130328-MS	Protein Zfp23
msc	MSC	MSC	20130328-MS	Metalloprotei
msc	MSC	MSC	20130328-MS	Adenosylhom
msc	MSC	MSC	20130328-MS	Isoform 2 of C
msc	MSC	MSC	20130328-MS	Protein Dag1 (
msc	MSC	MSC	20130328-MS	Actin related p
msc	MSC	MSC	20130328-MS	D-dopachrom

msc	MSC	MSC	20130328-MS	Hemoglobin s
msc	MSC	MSC	20130328-MS	Ab2-162 OS=F
msc	MSC	MSC	20130328-MS	Fructose-bispl
msc	MSC	MSC	20130328-MS	78 kDa glucos
msc	MSC	MSC	20130328-MS	Aminoacylase
msc	MSC	MSC	20130328-MS	Protein Col5a
msc	MSC	MSC	20130328-MS	Uncharacteriz
msc	MSC	MSC	20130328-MS	Transgelin OS
msc	MSC	MSC	20130328-MS	Plectin (Fragm
msc	MSC	MSC	20130328-MS	Isoform 2 of A
msc	MSC	MSC	20130328-MS	EGF-containin
msc	MSC	MSC	20130328-MS	Keratin, type I
msc	MSC	MSC	20130328-MS	Fatty acid-bin
msc	MSC	MSC	20130328-MS	Protein Pxdn
msc	MSC	MSC	20130328-MS	Protein Ccbe1
msc	MSC	MSC	20130328-MS	Keratin, type I
msc	MSC	MSC	20130328-MS	Protein Krt16
msc	MSC	MSC	20130328-MS	Periostin, oste
msc	MSC	MSC	20130328-MS	Protein S100-
msc	MSC	MSC	20130328-MS	Ribonuclease
msc	MSC	MSC	20130328-MS	Myosin light p
msc	MSC	MSC	20130328-MS	Glutathione S
msc	MSC	MSC	20130328-MS	Ferritin OS=Ra
msc	MSC	MSC	20130328-MS	Collagen alpha
msc	MSC	MSC	20130328-MS	Glutathione S
msc	MSC	MSC	20130328-MS	Myosin-9 OS=
msc	MSC	MSC	20130328-MS	Gamma-enola
msc	MSC	MSC	20130328-MS	Protein Rrbp1
msc	MSC	MSC	20130328-MS	Fetuin-B OS=F
msc	MSC	MSC	20130328-MS	Rab GDP disso
msc	Control	Control	091012_CTL-(	Protein LOC67
msc	Control	Control	091012_CTL-(	Uncharacteriz
msc	Control	Control	091012_CTL-(	Keratin, type I
msc	Control	Control	091012_CTL-(	Protein Rrbp1
msc	Control	Control	091012_CTL-(	Protein FAM1
msc	Control	Control	091012_CTL-(	Beta-galactosi
msc	Control	Control	091012_CTL-(	Mast cell carb
msc	Control	Control	091012_CTL-(	Cytochrome c
msc	Control	Control	091012_CTL-(	Procollagen-ly
msc	Control	Control	091012_CTL-(	Glyceraldehyc
msc	Control	Control	091012_CTL-(	Uncharacteriz
msc	Control	Control	091012_CTL-(	Neuronal cell
msc	Control	Control	091012_CTL-(	Fibronectin O
msc	Control	Control	091012_CTL-(	Protein Zfp23
msc	Control	Control	091012_CTL-(	Glutathione S
msc	Control	Control	091012_CTL-(	Plasminogen
msc	Control	Control	091012_CTL-(	Glutamate [N
msc	Control	Control	091012_CTL-(	Disintegrin an
msc	Control	Control	091012_CTL-(	Ig gamma-2A
msc	Control	Control	091012_CTL-(	Clathrin heavy
msc	Control	Control	091012_CTL-(	Glucose-6-pho

msc	Control	Control	091012_CTL-(	Guanine dean
msc	Control	Control	091012_CTL-(	Isoform 2 of G
msc	Control	Control	091012_CTL-(	Protein LOC1(
msc	Control	Control	091012_CTL-(	Olfactomedin-
msc	Control	Control	091012_CTL-(	Phosphorylase
msc	Control	Control	091012_CTL-(	Protein Col4a:
msc	Control	Control	091012_CTL-(	CArG-binding
msc	Control	Control	091012_CTL-(	Myosin light p
msc	Control	Control	091012_CTL-(	Alpha-1-inhibi
msc	Control	Control	091012_CTL-(	Fatty acid-bin
msc	Control	Control	091012_CTL-(	Acid ceramida
msc	Control	Control	091012_CTL-(	Latent transfo
msc	Control	Control	091012_CTL-(	60S acidic ribo
msc	Control	Control	091012_CTL-(	Glypican-1 OS
msc	Control	Control	091012_CTL-(	Protein Serpir
msc	Control	Control	091012_CTL-(	Elongation fac
msc	Control	Control	091012_CTL-(	Adipocyte enh
msc	Control	Control	091012_CTL-(	Uncharacteriz
msc	Control	Control	091012_CTL-(	Myosin-9 OS=
msc	Control	Control	091012_CTL-(	SPARC OS=Rat
msc	Control	Control	091012_CTL-(	Glypican 4 OS
msc	Control	Control	091012_CTL-(	Decorin OS=R
msc	Control	Control	091012_CTL-(	Matrix Gla pro
msc	Control	Control	091012_CTL-(	Fatty acid-bin
msc	Control	Control	091012_CTL-(	Profilin-1 OS=
msc	Control	Control	091012_CTL-(	14-3-3 proteir
msc	Control	Control	091012_CTL-(	Collagen alpha
msc	Control	Control	091012_CTL-(	Inter-alpha-try
msc	Control	Control	091012_CTL-(	Hexokinase-1
msc	Control	Control	091012_CTL-(	NADP-depend
msc	Control	Control	091012_CTL-(	Uncharacteriz
msc	Control	Control	091012_CTL-(	Plakophilin 1 (
msc	Control	Control	091012_CTL-(	Nucleoside dip
msc	Control	Control	091012_CTL-(	Lysyl oxidase-
msc	Control	Control	091012_CTL-(	Translationally
msc	Control	Control	091012_CTL-(	Bone morpho
msc	Control	Control	091012_CTL-(	A disintegrin a
msc	Control	Control	091012_CTL-(	Malate dehyd
msc	Control	Control	091012_CTL-(	Alpha-enolase
msc	Control	Control	091012_CTL-(	Protein Map2
msc	Control	Control	091012_CTL-(	Follistatin-rela
msc	Control	Control	091012_CTL-(	Alpha 4 type V
msc	Control	Control	091012_CTL-(	Alpha-interne
msc	Control	Control	091012_CTL-(	Afamin OS=Ra
msc	Control	Control	091012_CTL-(	Metalloprotei
msc	Control	Control	091012_CTL-(	Protein Col6a:
msc	Control	Control	091012_CTL-(	Peroxiredoxin
msc	Control	Control	091012_CTL-(	Oligodendrocy
msc	Control	Control	091012_CTL-(	Uncharacteriz
msc	Control	Control	091012_CTL-(	L-lactate dehy
msc	Control	Control	091012_CTL-(	Connective tis

msc	Control	Control	091012_CTL-(	FK506 binding
msc	Control	Control	091012_CTL-(	Plectin (Fragm
msc	Control	Control	091012_CTL-(	Peptidyl-proly
msc	Control	Control	091012_CTL-(	Nucleobindin-
msc	Control	Control	091012_CTL-(	Galectin-1 OS
msc	Control	Control	091012_CTL-(	Fructose-bispl
msc	Control	Control	091012_CTL-(	Protein Hspg2
msc	Control	Control	091012_CTL-(	Rabphilin-3A (
msc	Control	Control	091012_CTL-(	Keratin, type I
msc	Control	Control	091012_CTL-(	Heat shock pr
msc	Control	Control	091012_CTL-(	Alpha-2-HS-gl
msc	Control	Control	091012_CTL-(	Complement (
msc	Control	Control	091012_CTL-(	Biglycan OS=R
msc	Control	Control	091012_CTL-(	Calmodulin O:
msc	Control	Control	091012_CTL-(	Moesin (Fragr
msc	Control	Control	091012_CTL-(	Beta-2-microg
msc	Control	Control	091012_CTL-(	Extracellular s
msc	Control	Control	091012_CTL-(	Cathepsin D C
msc	Control	Control	091012_CTL-(	Uncharacteriz
msc	Control	Control	091012_CTL-(	Protein Krt16
msc	Control	Control	091012_CTL-(	Protein Krt31
msc	Control	Control	091012_CTL-(	Platelet-derive
msc	Control	Control	091012_CTL-(	Uncharacteriz
msc	Control	Control	091012_CTL-(	Aggrecan core
msc	Control	Control	091012_CTL-(	Phosphoglyce
msc	Control	Control	091012_CTL-(	Histone H2A t
msc	Control	Control	091012_CTL-(	Tubulin alpha-
msc	Control	Control	091012_CTL-(	Protein Lamb1
msc	Control	Control	091012_CTL-(	Keratin, type I
msc	Control	Control	091012_CTL-(	Uncharacteriz
msc	Control	Control	091012_CTL-(	Collagen alpha
msc	Control	Control	091012_CTL-(	Procollagen, t
msc	Control	Control	091012_CTL-(	Isoform B of Ir
msc	Control	Control	091012_CTL-(	Complement (
msc	Control	Control	091012_CTL-(	Cathepsin B O
msc	Control	Control	091012_CTL-(	Fibulin 1 (Prec
msc	Control	Control	091012_CTL-(	Uncharacteriz
msc	Control	Control	091012_CTL-(	Glial fibrillary
msc	Control	Control	091012_CTL-(	Lumican OS=R
msc	Control	Control	091012_CTL-(	Keratin, type I
msc	Control	Control	091012_CTL-(	Triosephospha
msc	Control	Control	091012_CTL-(	Fibronectin ty
msc	Control	Control	091012_CTL-(	Protein DJ-1 C
msc	Control	Control	091012_CTL-(	Alpha-1-macr
msc	Control	Control	091012_CTL-(	Isoform 3 of N
msc	Control	Control	091012_CTL-(	Junction plakc
msc	Control	Control	091012_CTL-(	Polyubiquitin-
msc	Control	Control	091012_CTL-(	Protein Itih2 C
msc	Control	Control	091012_CTL-(	Collagen alpha
msc	Control	Control	091012_CTL-(	Uncharacteriz
msc	Control	Control	091012_CTL-(	Transgelin OS

msc	Control	Control	091012_CTL-(	Lactadherin O
msc	Control	Control	091012_CTL-(	Latent-transfo
msc	Control	Control	091012_CTL-(	Granulins OS=
msc	Control	Control	091012_CTL-(	Protein Flnc O
msc	Control	Control	091012_CTL-(	Growth arrest
msc	Control	Control	091012_CTL-(	Sodium/potas
msc	Control	Control	091012_CTL-(	Neuropilin-2 (
msc	Control	Control	091012_CTL-(	Protein S100-/
msc	Control	Control	091012_CTL-(	Epididymal se
msc	Control	Control	091012_CTL-(	Fibronectin O:
msc	Control	Control	091012_CTL-(	Protein-lysine
msc	Control	Control	091012_CTL-(	Vinculin OS=R
msc	Control	Control	091012_CTL-(	Filamin alpha
msc	Control	Control	091012_CTL-(	Annexin A1 O:
msc	Control	Control	091012_CTL-(	Vimentin OS=
msc	Control	Control	091012_CTL-(	Cystatin-C OS:
msc	Control	Control	091012_CTL-(	Alcohol dehyc
msc	Control	Control	091012_CTL-(	Histone H4 O!
msc	Control	Control	091012_CTL-(	Hemoglobin s
msc	Control	Control	091012_CTL-(	Periostin, oste
msc	Control	Control	091012_CTL-(	Collagen alph:
msc	Control	Control	091012_CTL-(	Anionic trypsi
msc	Control	Control	091012_CTL-(	Fibulin-5 OS=I
msc	Control	Control	091012_CTL-(	Metalloprotei
msc	Control	Control	091012_CTL-(	Protein NOV I
msc	Control	Control	091012_CTL-(	Purine nucleo
msc	Control	Control	091012_CTL-(	Collagen alph:
msc	Control	Control	091012_CTL-(	Macrophage r
msc	Control	Control	091012_CTL-(	Actin, aortic s
msc	Control	Control	091012_CTL-(	Protein Lama4
msc	Control	Control	091012_CTL-(	Uncharacteriz
msc	Control	Control	091012_CTL-(	Rab GDP disso
msc	Control	Control	091012_CTL-(	Superoxide di:
msc	Control	Control	091012_CTL-(	Phosphatidyle
msc	Control	Control	091012_CTL-(	Apolipoprotei
msc	Control	Control	091012_CTL-(	Keratin, type I
msc	Control	Control	091012_CTL-(	Plasminogen a
msc	Control	Control	091012_CTL-(	Protein Col8a1
msc	Control	Control	091012_CTL-(	LIM and SH3 c
msc	Control	Control	091012_CTL-(	Calreticulin O!
msc	Control	Control	091012_CTL-(	Cathepsin L1 (
msc	Control	Control	091012_CTL-(	WD repeat-co
msc	Control	Control	091012_CTL-(	Keratin, type I
msc	Control	Control	091012_CTL-(	Protein Dkk3 (
msc	Control	Control	091012_CTL-(	Uncharacteriz
msc	Control	Control	091012_CTL-(	Keratin, type I
msc	Control	Control	091012_CTL-(	14-3-3 proteir
msc	Control	Control	091012_CTL-(	Keratin, type I
msc	Control	Control	091012_CTL-(	EGF-containin
msc	Control	Control	091012_CTL-(	Transcobalam
msc	Control	Control	091012_CTL-(	Procollagen, t

msc	Control	Control	091012_CTL-(	Ribonuclease
msc	Control	Control	091012_CTL-(	Protein Krt76
msc	Control	Control	091012_CTL-(	Uncharacteriz
msc	Control	Control	091012_CTL-(	Procollagen-ly
msc	Control	Control	091012_CTL-(	Coiled-coil do
msc	Control	Control	091012_CTL-(	Microtubule-a
msc	Control	Control	091012_CTL-(	Ubiquitin-like
msc	Control	Control	091012_CTL-(	Tropomyosin
msc	Control	Control	091012_CTL-(	Alpha-2-macr
msc	Control	Control	091012_CTL-(	Protein Col6a
msc	Control	Control	091012_CTL-(	Neurofilamen
msc	Control	Control	091012_CTL-(	Uncharacteriz
msc	Control	Control	091012_CTL-(	Serotransferri
msc	Control	Control	091012_CTL-(	Nucleoside di
msc	Control	Control	091012_CTL-(	Procollagen C
msc	Control	Control	091012_CTL-(	Nidogen-1 OS
msc	Control	Control	091012_CTL-(	Thy-1 membra
msc	Control	Control	091012_CTL-(	Heat shock co
msc	Control	Control	091012_CTL-(	Peptidyl-proly
msc	Control	Control	091012_CTL-(	Uncharacteriz
msc	Control	Control	091012_CTL-(	Gremlin-1 OS=
msc	Control	Control	091012_CTL-(	Destrin OS=Ra
msc	Control	Control	091012_CTL-(	Protein Thbs2
msc	Control	Control	091012_CTL-(	Thymosin beta
msc	Control	Control	091012_CTL-(	Ab2-162 OS=F
msc	Control	Control	091012_CTL-(	Histone H2B t
msc	Control	Control	091012_CTL-(	Serine proteas
msc	Control	Control	091012_CTL-(	Elongation fac
msc	Control	Control	091012_CTL-(	Nucleobindin-
msc	Control	Control	091012_CTL-(	Uncharacteriz
msc	Control	Control	091012_CTL-(	Adenylate kin
msc	Control	Control	091012_CTL-(	Ester hydrolas
msc	Control	Control	091012_CTL-(	Uncharacteriz
msc	Control	Control	091012_CTL-(	Placenta grow
msc	Control	Control	091012_CTL-(	RCG32401, isc
msc	Control	Control	091012_CTL-(	Hemoglobin s
msc	Control	Control	091012_CTL-(	Transforming
msc	Control	Control	091012_CTL-(	Keratin, type I
msc	Control	Control	091012_CTL-(	Uncharacteriz
msc	Control	Control	091012_CTL-(	Protein Ccbe1
msc	Control	Control	091012_CTL-(	C-type lectin c
msc	Control	Control	091012_CTL-(	Calsyntenin-1
msc	Control	Control	091012_CTL-(	Procollagen, t
msc	Control	Control	091012_CTL-(	Type II keratir
msc	Control	Control	091012_CTL-(	Dihydropyrim
msc	Control	Control	091012_CTL-(	Collagen alpha
msc	Control	Control	091012_CTL-(	Sushi-repeat-c
msc	Control	Control	091012_CTL-(	Keratin, type I
msc	Control	Control	091012_CTL-(	Peptidyl-proly
msc	Control	Control	091012_CTL-(	Follistatin-rela
msc	Control	Control	091012_CTL-(	Collagen alpha

msc	Control	Control	091012_CTL-(	Collagen alpha
msc	Control	Control	091012_CTL-(	14-3-3 protein
msc	Control	Control	091012_CTL-(	Myelin-associa
msc	Control	Control	091012_CTL-(	Ubiquitin carb
msc	Control	Control	091012_CTL-(	NAD(P)H dehy
msc	Control	Control	091012_CTL-(	Protein Dag1 (
msc	Control	Control	091012_CTL-(	Heat shock pr
msc	Control	Control	091012_CTL-(	14-3-3 protein
msc	Control	Control	091012_CTL-(	72 kDa type IV
msc	Control	Control	091012_CTL-(	Tubulin alpha-
msc	Control	Control	091012_CTL-(	Keratin, type I
msc	Control	Control	091012_CTL-(	Complement (
msc	Control	Control	091012_CTL-(	Rho GDP-disso
msc	Control	Control	091012_CTL-(	Vitamin D-bin
msc	Control	Control	091012_CTL-(	Protein Ncam
msc	Control	Control	091012_CTL-(	Protein LOC1(
msc	Control	Control	091012_CTL-(	Inhibin beta A
msc	Control	Control	091012_CTL-(	Serine (Or cys
msc	Control	Control	091012_CTL-(	Cysteine and g
msc	Control	Control	091012_CTL-(	Keratin, type I
msc	Control	Control	091012_CTL-(	78 kDa glucos
msc	Control	Control	091012_CTL-(	Peroxiredoxin
msc	Control	Control	091012_CTL-(	HtrA serine pe
msc	Control	Control	091012_CTL-(	Alpha-actinin-
msc	Control	Control	091012_CTL-(	Protein Pxdn (
msc	Control	Control	091012_CTL-(	Uncharacteriz
msc	Control	Control	091012_CTL-(	Isoform TGF-b
msc	Control	Control	091012_CTL-(	Clusterin OS=I
msc	Control	Control	091012_CTL-(	Protein Dsp O
msc	Control	Control	091012_CTL-(	Isoform 2 of T
msc	Control	Control	091012_CTL-(	Tubulin beta-2
msc	Control	Control	091012_CTL-(	Peripherin OS
msc	Control	Control	091012_CTL-(	Calumenin OS
msc	Control	Control	091012_CTL-(	Sulfated glyco
msc	Control	Control	091012_CTL-(	C1qtnf3 prote
msc	Control	Control	091012_CTL-(	Fibulin 2, isofo
msc	Control	Control	091012_CTL-(	Dipeptidyl pep
msc	Control	Control	091012_CTL-(	Uncharacteriz
msc	Control	Control	091012_CTL-(	Alpha-actinin-
msc	Control	Control	091012_CTL-(	Neurofilamen
msc	Control	Control	091012_CTL-(	L-lactate dehy
msc	Control	Control	091012_CTL-(	Secernin-1 OS
msc	Control	Control	091012_CTL-(	Insulin-like gro
msc	Control	Control	091012_CTL-(	Annexin A2 OS
msc	Control	Control	091012_CTL-(	Serum albumi
msc	Control	Control	091012_CTL-(	Phosphoglyce
msc	Control	Control	091012_CTL-(	Tropomyosin
msc	Control	Control	091012_CTL-(	Cofilin-1 OS=R
msc	Control	Control	091012_CTL-(	Osteopontin O
msc	Control	Control	091012_CTL-(	Uncharacteriz
msc	Control	Control	091012_CTL-(	Peptidyl-proly

msc	Control	Control	091012_CTL-(	Protein Col5a:
msc	Control	Control	091012_CTL-(	Actin, cytopla:
msc	Control	Control	091012_CTL-(	Legumain OS=
msc	Control	Control	091012_CTL-(	Protein Col4a:
msc	Control	Control	091012_CTL-(	Protein Lamc1
msc	Control	Control	091012_CTL-(	Keratin, type I
msc	Control	Control	091012_CTL-(	Gamma-enola
msc	Control	Control	091012_CTL-(	Plastin 3 (T-isc
msc	Control	Control	091012_CTL-(	Isoform 3 of T
msc	Control	Control	091012_CTL-(	Isoform V3 of
msc	SCI	SCI	091012_L-02	Inter-alpha try
msc	SCI	SCI	091012_L-02	Uncharacteriz
msc	SCI	SCI	091012_L-02	Alpha-2-HS-gly
msc	SCI	SCI	091012_L-02	Microtubule-a
msc	SCI	SCI	091012_L-02	Adenosylhom
msc	SCI	SCI	091012_L-02	Ester hydrolas
msc	SCI	SCI	091012_L-02	Myelin-associ
msc	SCI	SCI	091012_L-02	Protein Sh3bg
msc	SCI	SCI	091012_L-02	Protein Ank2 (
msc	SCI	SCI	091012_L-02	Aspartate ami
msc	SCI	SCI	091012_L-02	Isoform 2 of P
msc	SCI	SCI	091012_L-02	Peroxiredoxin
msc	SCI	SCI	091012_L-02	L-lactate dehy
msc	SCI	SCI	091012_L-02	Uncharacteriz
msc	SCI	SCI	091012_L-02	Calreticulin OS
msc	SCI	SCI	091012_L-02	Protein Col6a:
msc	SCI	SCI	091012_L-02	4-trimethylam
msc	SCI	SCI	091012_L-02	Protein LOC1(
msc	SCI	SCI	091012_L-02	Thioredoxin-li
msc	SCI	SCI	091012_L-02	WD repeat-co
msc	SCI	SCI	091012_L-02	Calretinin OS=
msc	SCI	SCI	091012_L-02	Protein Sema:
msc	SCI	SCI	091012_L-02	Phosphoglyce
msc	SCI	SCI	091012_L-02	Glyceraldehyc
msc	SCI	SCI	091012_L-02	Ig gamma-2A
msc	SCI	SCI	091012_L-02	Keratin, type I
msc	SCI	SCI	091012_L-02	Hydroxyacyl g
msc	SCI	SCI	091012_L-02	Protein Serpir
msc	SCI	SCI	091012_L-02	Aminoacylase
msc	SCI	SCI	091012_L-02	Glial fibrillary
msc	SCI	SCI	091012_L-02	Protein AMBP
msc	SCI	SCI	091012_L-02	Keratin, type I
msc	SCI	SCI	091012_L-02	Neuronal cell
msc	SCI	SCI	091012_L-02	Peripherin OS
msc	SCI	SCI	091012_L-02	Alpha-1-macr(
msc	SCI	SCI	091012_L-02	Latexin OS=Ra
msc	SCI	SCI	091012_L-02	Ectonucleotid
msc	SCI	SCI	091012_L-02	Glutathione S-
msc	SCI	SCI	091012_L-02	Protein SET O:
msc	SCI	SCI	091012_L-02	Neurofilamen
msc	SCI	SCI	091012_L-02	Plectin (Fragm

msc	SCI	SCI	091012_L-02	Myosin-9 OS=
msc	SCI	SCI	091012_L-02	78 kDa glucos
msc	SCI	SCI	091012_L-02	Glucose-6-pho
msc	SCI	SCI	091012_L-02	Guanine dean
msc	SCI	SCI	091012_L-02	Protein Ncam
msc	SCI	SCI	091012_L-02	Isoform 2 of G
msc	SCI	SCI	091012_L-02	F-actin-cappir
msc	SCI	SCI	091012_L-02	Uncharacteriz
msc	SCI	SCI	091012_L-02	Neurofilamen
msc	SCI	SCI	091012_L-02	Isoform Gamr
msc	SCI	SCI	091012_L-02	14-3-3 proteir
msc	SCI	SCI	091012_L-02	Glutathione S-
msc	SCI	SCI	091012_L-02	Complement (
msc	SCI	SCI	091012_L-02	Creatine kinas
msc	SCI	SCI	091012_L-02	Tubulin alpha-
msc	SCI	SCI	091012_L-02	Gamma-enola
msc	SCI	SCI	091012_L-02	Isoform Crk-I (
msc	SCI	SCI	091012_L-02	Hexokinase-1
msc	SCI	SCI	091012_L-02	Protein Txndc
msc	SCI	SCI	091012_L-02	Neuromodulir
msc	SCI	SCI	091012_L-02	Myosin light p
msc	SCI	SCI	091012_L-02	Vitamin D-bin
msc	SCI	SCI	091012_L-02	Hemoglobin s
msc	SCI	SCI	091012_L-02	Adenylate kin
msc	SCI	SCI	091012_L-02	Protein Itih4 (
msc	SCI	SCI	091012_L-02	Apolipoprotei
msc	SCI	SCI	091012_L-02	Calpain small
msc	SCI	SCI	091012_L-02	Alpha-1-inhibi
msc	SCI	SCI	091012_L-02	Annexin A5 O:
msc	SCI	SCI	091012_L-02	Fibrinogen be
msc	SCI	SCI	091012_L-02	Protein DJ-1 C
msc	SCI	SCI	091012_L-02	Fructose-bispl
msc	SCI	SCI	091012_L-02	Serotransferri
msc	SCI	SCI	091012_L-02	Complement f
msc	SCI	SCI	091012_L-02	Rab GDP disso
msc	SCI	SCI	091012_L-02	Synaptosomal
msc	SCI	SCI	091012_L-02	Latent transfo
msc	SCI	SCI	091012_L-02	Isoform 3 of N
msc	SCI	SCI	091012_L-02	Procollagen, t
msc	SCI	SCI	091012_L-02	Glutathione S-
msc	SCI	SCI	091012_L-02	Acyl-CoA-bind
msc	SCI	SCI	091012_L-02	Isoform 2 of S
msc	SCI	SCI	091012_L-02	Fibrinogen alp
msc	SCI	SCI	091012_L-02	Acylphosphat:
msc	SCI	SCI	091012_L-02	6-phosphoglu
msc	SCI	SCI	091012_L-02	Granulins OS=
msc	SCI	SCI	091012_L-02	Cysteine and g
msc	SCI	SCI	091012_L-02	Transcriptiona
msc	SCI	SCI	091012_L-02	Dihydropyrim
msc	SCI	SCI	091012_L-02	Rab GDP disso
msc	SCI	SCI	091012_L-02	Keratin, type I

msc	SCI	SCI	091012_L-02	Hyaluronan ar
msc	SCI	SCI	091012_L-02	2',3'-cyclic-nu
msc	SCI	SCI	091012_L-02	Transgelin OS
msc	SCI	SCI	091012_L-02	Triosephosph
msc	SCI	SCI	091012_L-02	Uncharacteriz
msc	SCI	SCI	091012_L-02	Protein Itih2 (
msc	SCI	SCI	091012_L-02	Uncharacteriz
msc	SCI	SCI	091012_L-02	Tubulin beta-!
msc	SCI	SCI	091012_L-02	Uncharacteriz
msc	SCI	SCI	091012_L-02	Oligodendroc
msc	SCI	SCI	091012_L-02	Heat shock pr
msc	SCI	SCI	091012_L-02	Ig gamma-1 cl
msc	SCI	SCI	091012_L-02	Uncharacteriz
msc	SCI	SCI	091012_L-02	Transaldolase
msc	SCI	SCI	091012_L-02	Glutamate de
msc	SCI	SCI	091012_L-02	Fascin OS=Rat
msc	SCI	SCI	091012_L-02	Elongation fac
msc	SCI	SCI	091012_L-02	Elongation fac
msc	SCI	SCI	091012_L-02	Isoform B of A
msc	SCI	SCI	091012_L-02	Collagen alpha
msc	SCI	SCI	091012_L-02	Myelin protec
msc	SCI	SCI	091012_L-02	Tenascin-R OS
msc	SCI	SCI	091012_L-02	Rabphilin-3A (
msc	SCI	SCI	091012_L-02	Osteoglycin (F
msc	SCI	SCI	091012_L-02	Glyoxalase do
msc	SCI	SCI	091012_L-02	Vesicle-fusing
msc	SCI	SCI	091012_L-02	Fatty acid-bin
msc	SCI	SCI	091012_L-02	Kininogen 1 O
msc	SCI	SCI	091012_L-02	Keratin, type I
msc	SCI	SCI	091012_L-02	Serine protea
msc	SCI	SCI	091012_L-02	Isoform Glt-1A
msc	SCI	SCI	091012_L-02	Cathepsin D C
msc	SCI	SCI	091012_L-02	Glutathione S
msc	SCI	SCI	091012_L-02	Aggrecan core
msc	SCI	SCI	091012_L-02	Spectrin alpha
msc	SCI	SCI	091012_L-02	Ubiquitin carb
msc	SCI	SCI	091012_L-02	Vimentin OS=
msc	SCI	SCI	091012_L-02	Protein Col4a:
msc	SCI	SCI	091012_L-02	Complement (
msc	SCI	SCI	091012_L-02	Lysozyme C-1
msc	SCI	SCI	091012_L-02	Heat shock co
msc	SCI	SCI	091012_L-02	Isoform 2 of N
msc	SCI	SCI	091012_L-02	Histone H3 OS
msc	SCI	SCI	091012_L-02	Complement (
msc	SCI	SCI	091012_L-02	Alpha-centrac
msc	SCI	SCI	091012_L-02	Actin, cytopla:
msc	SCI	SCI	091012_L-02	Histone H4 OS
msc	SCI	SCI	091012_L-02	Galectin-1 OS
msc	SCI	SCI	091012_L-02	Syntaxin-1B O
msc	SCI	SCI	091012_L-02	Protein LOC1(
msc	SCI	SCI	091012_L-02	Purine nucleo

msc	SCI	SCI	091012_L-02	Histone H2B t
msc	SCI	SCI	091012_L-02	Filamin alpha
msc	SCI	SCI	091012_L-02	Keratin, type I
msc	SCI	SCI	091012_L-02	Uncharacteriz
msc	SCI	SCI	091012_L-02	CD9 antigen C
msc	SCI	SCI	091012_L-02	Annexin A2 OS
msc	SCI	SCI	091012_L-02	Protein Cfh OS
msc	SCI	SCI	091012_L-02	Annexin A1 OS
msc	SCI	SCI	091012_L-02	Protein Lamc1
msc	SCI	SCI	091012_L-02	Actin, aortic s
msc	SCI	SCI	091012_L-02	Uncharacteriz
msc	SCI	SCI	091012_L-02	Transgelin-3 C
msc	SCI	SCI	091012_L-02	Rho GDP-disso
msc	SCI	SCI	091012_L-02	Cofilin-1 OS=R
msc	SCI	SCI	091012_L-02	Neurotrimin C
msc	SCI	SCI	091012_L-02	Alpha 4 type V
msc	SCI	SCI	091012_L-02	Afamin OS=Ra
msc	SCI	SCI	091012_L-02	Pyruvate kinas
msc	SCI	SCI	091012_L-02	Apolipoprotei
msc	SCI	SCI	091012_L-02	14-3-3 protein
msc	SCI	SCI	091012_L-02	Dihydropterid
msc	SCI	SCI	091012_L-02	Moesin (Fragn
msc	SCI	SCI	091012_L-02	Procollagen, t
msc	SCI	SCI	091012_L-02	Isoform Tau-B
msc	SCI	SCI	091012_L-02	Protein Psat1
msc	SCI	SCI	091012_L-02	Coiled-coil do
msc	SCI	SCI	091012_L-02	Serum albumi
msc	SCI	SCI	091012_L-02	Cell cycle exit
msc	SCI	SCI	091012_L-02	Carbonic anhy
msc	SCI	SCI	091012_L-02	Protein Ube2l
msc	SCI	SCI	091012_L-02	Protein Col6a3
msc	SCI	SCI	091012_L-02	Isoform 2 of C
msc	SCI	SCI	091012_L-02	Annexin (Frag
msc	SCI	SCI	091012_L-02	Myoglobin OS
msc	SCI	SCI	091012_L-02	NADP-depend
msc	SCI	SCI	091012_L-02	Peroxiredoxin
msc	SCI	SCI	091012_L-02	Lactoylglutath
msc	SCI	SCI	091012_L-02	Brevican core
msc	SCI	SCI	091012_L-02	Protein disulfi
msc	SCI	SCI	091012_L-02	Isoform M2 of
msc	SCI	SCI	091012_L-02	Serine proteas
msc	SCI	SCI	091012_L-02	Dihydropyrim
msc	SCI	SCI	091012_L-02	Polyubiquitin-
msc	SCI	SCI	091012_L-02	ATP synthase
msc	SCI	SCI	091012_L-02	Uncharacteriz
msc	SCI	SCI	091012_L-02	Carboxylester
msc	SCI	SCI	091012_L-02	Ferritin OS=Ra
msc	SCI	SCI	091012_L-02	Transketolase
msc	SCI	SCI	091012_L-02	Transitional er
msc	SCI	SCI	091012_L-02	Uncharacteriz
msc	SCI	SCI	091012_L-02	Dihydropyrim

msc	SCI	SCI	091012_L-02	Cathepsin B O
msc	SCI	SCI	091012_L-02	Peptidyl-proly
msc	SCI	SCI	091012_L-02	Ribonuclease
msc	SCI	SCI	091012_L-02	Thioredoxin O
msc	SCI	SCI	091012_L-02	Amphiphysin
msc	SCI	SCI	091012_L-02	Protein Hepac
msc	SCI	SCI	091012_L-02	Keratin, type I
msc	SCI	SCI	091012_L-02	Fibulin 1 (Prec
msc	SCI	SCI	091012_L-02	Uncharacteriz
msc	SCI	SCI	091012_L-02	Sodium/potas
msc	SCI	SCI	091012_L-02	Hyaluronan ar
msc	SCI	SCI	091012_L-02	Hemoglobin s
msc	SCI	SCI	091012_L-02	Creatine kinas
msc	SCI	SCI	091012_L-02	Phosphoglyce
msc	SCI	SCI	091012_L-02	L-lactate dehy
msc	SCI	SCI	091012_L-02	Protein Zc3h1
msc	SCI	SCI	091012_L-02	Fetuin-B OS=F
msc	SCI	SCI	091012_L-02	Malate dehyd
msc	SCI	SCI	091012_L-02	Protein Serpir
msc	SCI	SCI	091012_L-02	Phosphoglyce
msc	SCI	SCI	091012_L-02	Legumain OS=
msc	SCI	SCI	091012_L-02	Protein Col8a
msc	SCI	SCI	091012_L-02	Serine proteas
msc	SCI	SCI	091012_L-02	Keratin, type I
msc	SCI	SCI	091012_L-02	Keratin, type I
msc	SCI	SCI	091012_L-02	Protein Npepp
msc	SCI	SCI	091012_L-02	Protein IMPA(
msc	SCI	SCI	091012_L-02	Tropomyosin
msc	SCI	SCI	091012_L-02	Neural cell ad
msc	SCI	SCI	091012_L-02	Xaa-Pro dipep
msc	SCI	SCI	091012_L-02	Tubulin beta-
msc	SCI	SCI	091012_L-02	Lumican OS=F
msc	SCI	SCI	091012_L-02	ATP synthase
msc	SCI	SCI	091012_L-02	Myristoylated
msc	SCI	SCI	091012_L-02	Uncharacteriz
msc	SCI	SCI	091012_L-02	Cathepsin L1 (
msc	SCI	SCI	091012_L-02	Glycogen pho
msc	SCI	SCI	091012_L-02	Vinculin OS=R
msc	SCI	SCI	091012_L-02	Lymphocyte c
msc	SCI	SCI	091012_L-02	Copper transp
msc	SCI	SCI	091012_L-02	Isoform 2 of T
msc	SCI	SCI	091012_L-02	Phosphogluco
msc	SCI	SCI	091012_L-02	Sodium/potas
msc	SCI	SCI	091012_L-02	Malate dehyd
msc	SCI	SCI	091012_L-02	Keratin, type I
msc	SCI	SCI	091012_L-02	Protein S100-
msc	SCI	SCI	091012_L-02	Cytosolic non-
msc	SCI	SCI	091012_L-02	Tropomyosin
msc	SCI	SCI	091012_L-02	Microtubule-a
msc	SCI	SCI	091012_L-02	Profilin-2 OS=
msc	SCI	SCI	091012_L-02	Murinoglobuli

msc	SCI	SCI	091012_L-02	Peroxiredoxin
msc	SCI	SCI	091012_L-02	Protein Krt86
msc	SCI	SCI	091012_L-02	Glutamine syr
msc	SCI	SCI	091012_L-02	14-3-3 proteir
msc	SCI	SCI	091012_L-02	Nidogen-1 OS
msc	SCI	SCI	091012_L-02	Alpha-actinin-
msc	SCI	SCI	091012_L-02	Alpha-2-macr
msc	SCI	SCI	091012_L-02	Dihydrolipoyl
msc	SCI	SCI	091012_L-02	Apolipoprotei
msc	SCI	SCI	091012_L-02	Complement (
msc	SCI	SCI	091012_L-02	Protein Atp6v
msc	SCI	SCI	091012_L-02	Ig gamma-2B
msc	SCI	SCI	091012_L-02	Plasminogen (
msc	SCI	SCI	091012_L-02	Alpha-actinin-
msc	SCI	SCI	091012_L-02	Fibronectin O!
msc	SCI	SCI	091012_L-02	Protein Col5a:
msc	SCI	SCI	091012_L-02	Heme binding
msc	SCI	SCI	091012_L-02	Uncharacteriz
msc	SCI	SCI	091012_L-02	Ab2-162 OS=F
msc	SCI	SCI	091012_L-02	Phosphorylase
msc	SCI	SCI	091012_L-02	Protein disulfi
msc	SCI	SCI	091012_L-02	Carbonic anhy
msc	SCI	SCI	091012_L-02	Uncharacteriz
msc	SCI	SCI	091012_L-02	Microtubule-a
msc	SCI	SCI	091012_L-02	Transgelin-2 (
msc	SCI	SCI	091012_L-02	Contactin-1 O
msc	SCI	SCI	091012_L-02	Transthyretin
msc	SCI	SCI	091012_L-02	Protein Dsp O
msc	SCI	SCI	091012_L-02	Aconitate hyd
msc	SCI	SCI	091012_L-02	Sodium/potas
msc	SCI	SCI	091012_L-02	Inter-alpha-try
msc	SCI	SCI	091012_L-02	Complexin-1 (
msc	SCI	SCI	091012_L-02	Acylphosphata
msc	SCI	SCI	091012_L-02	CD59 glycopro
msc	SCI	SCI	091012_L-02	Chaperonin co
msc	SCI	SCI	091012_L-02	Epididymal se
msc	SCI	SCI	091012_L-02	Protein RGD1!
msc	SCI	SCI	091012_L-02	Plasminogen a
msc	SCI	SCI	091012_L-02	Isoform 3 of T
msc	SCI	SCI	091012_L-02	Hemoglobin s
msc	SCI	SCI	091012_L-02	Protein S100-/
msc	SCI	SCI	091012_L-02	Neurocan core
msc	SCI	SCI	091012_L-02	Plastin 3 (T-isc
msc	SCI	SCI	091012_L-02	Fibulin 2, isofc
msc	SCI	SCI	091012_L-02	Calcium/calmc
msc	SCI	SCI	091012_L-02	Galectin-3 OS=
msc	SCI	SCI	091012_L-02	Cell adhesion
msc	SCI	SCI	091012_L-02	Isoform V3 of
msc	SCI	SCI	091012_L-02	60S acidic ribc
msc	SCI	SCI	091012_L-02	C-reactive prc
msc	SCI	SCI	091012_L-02	14-3-3 proteir

msc	SCI	SCI	091012_L-02	Protein S100a
msc	SCI	SCI	091012_L-02	Destrin OS=Ra
msc	SCI	SCI	091012_L-02	Hemopexin O
msc	SCI	SCI	091012_L-02	Proteasome s
msc	SCI	SCI	091012_L-02	Tubulin polym
msc	SCI	SCI	091012_L-02	Aldose reduct
msc	SCI	SCI	091012_L-02	14-3-3 protein
msc	SCI	SCI	091012_L-02	Microtubule-a
msc	SCI	SCI	091012_L-02	Fatty acid-bin
msc	SCI	SCI	091012_L-02	Ig lambda-2 cl
msc	SCI	SCI	091012_L-02	Macrophage-c
msc	SCI	SCI	091012_L-02	Macrophage r
msc	SCI	SCI	091012_L-02	Isoform IB of S
msc	SCI	SCI	091012_L-02	Alpha-interne
msc	SCI	SCI	091012_L-02	Protein S100-A
msc	SCI	SCI	091012_L-02	Glycerol-3-pho
msc	SCI	SCI	091012_L-02	Beta-enolase
msc	SCI	SCI	091012_L-02	Translationally
msc	SCI	SCI	091012_L-02	Gamma-synuc
msc	SCI	SCI	091012_L-02	Reticulon-4 O
msc	SCI	SCI	091012_L-02	Protein Lamb1
msc	SCI	SCI	091012_L-02	6-phosphoglu
msc	SCI	SCI	091012_L-02	Profilin-1 OS=
msc	SCI	SCI	091012_L-02	Alpha globin C
msc	SCI	SCI	091012_L-02	Superoxide di
msc	SCI	SCI	091012_L-02	Prelamin-A/C
msc	SCI	SCI	091012_L-02	Inositol mono
msc	SCI	SCI	091012_L-02	Adenylyl cycla
msc	SCI	SCI	091012_L-02	14-3-3 protein
msc	SCI	SCI	091012_L-02	Adenine phos
msc	SCI	SCI	091012_L-02	Biliverdin redu
msc	SCI	SCI	091012_L-02	Kallikrein 6, is
msc	SCI	SCI	091012_L-02	Alcohol dehyd
msc	SCI	SCI	091012_L-02	Chloride intra
msc	SCI	SCI	091012_L-02	Ubiquitin-like
msc	SCI	SCI	091012_L-02	Kinesin-1 heav
msc	SCI	SCI	091012_L-02	Alpha-1-antip
msc	SCI	SCI	091012_L-02	Metalloprotei
msc	SCI	SCI	091012_L-02	Protein Rrbp1
msc	SCI	SCI	091012_L-02	CArG-binding
msc	SCI	SCI	091012_L-02	Fructose-bispl
msc	SCI	SCI	091012_L-02	Isoform 1 of C
msc	SCI	SCI	091012_L-02	Nucleoside dip
msc	SCI	SCI	091012_L-02	Secernin-1 OS
msc	SCI	SCI	091012_L-02	Tubulin beta-2
msc	SCI	SCI	091012_L-02	Ribonuclease
msc	SCI	SCI	091012_L-02	Phospholysine
msc	SCI	SCI	091012_L-02	Protein LOC67
msc	SCI	SCI	091012_L-02	Prostaglandin
msc	SCI	SCI	091012_L-02	Cystatin-C OS=
msc	SCI	SCI	091012_L-02	Clathrin heavy

msc	SCI	SCI	091012_L-02	T-kininogen 2
msc	SCI	SCI	091012_L-02	Protein CutA (
msc	SCI	SCI	091012_L-02	Protein Hbb-b
msc	SCI	SCI	091012_L-02	Protein Hspg2
msc	SCI	SCI	091012_L-02	Proteasome s
msc	SCI	SCI	091012_L-02	Serine/threon
msc	SCI	SCI	091012_L-02	C-C motif cher
msc	SCI	SCI	091012_L-02	Ubiquitin carb
msc	SCI	SCI	091012_L-02	Anionic trypsi
msc	SCI	SCI	091012_L-02	Alpha-1B-glyc
msc	SCI	SCI	091012_L-02	Apolipoprotei
msc	SCI	SCI	091012_L-02	Haptoglobin C
msc	SCI	SCI	091012_L-02	Dynein light cl
msc	SCI	SCI	091012_L-02	Tubulin beta-3
msc	SCI	SCI	091012_L-02	Neurofilamen
msc	SCI	SCI	091012_L-02	60S acidic ribo
msc	SCI	SCI	091012_L-02	Isoform 2 of A
msc	SCI	SCI	091012_L-02	Histone H2A t
msc	SCI	SCI	091012_L-02	Tubulin alpha-
msc	SCI	SCI	091012_L-02	Uncharacteriz
msc	SCI	SCI	091012_L-02	NSFL1 cofacto
msc	SCI	SCI	091012_L-02	Histidine-rich
msc	SCI	SCI	091012_L-02	Monocyte diff
msc	SCI	SCI	091012_L-02	Protein Flnc O
msc	SCI	SCI	091012_L-02	Protein LOC10
msc	SCI	SCI	091012_L-02	Cytochrome c
msc	SCI	SCI	091012_L-02	D-dopachrom
msc	SCI	SCI	091012_L-02	Nucleoside di
msc	SCI	SCI	091012_L-02	Ubiquitin thio
msc	SCI	SCI	091012_L-02	Carbonic anhy
msc	SCI	SCI	091012_L-02	Ig kappa chair
msc	SCI	SCI	091012_L-02	Phosphatidyle
msc	SCI	SCI	091012_L-02	Acidic leucine
msc	SCI	SCI	091012_L-02	Keratin, type I
msc	SCI	SCI	091012_L-02	V-type proton
msc	SCI	SCI	091012_L-02	Alpha-enolase
msc	SCI	SCI	091012_L-02	Isoform 2 of L
msc	SCI	SCI	091012_L-02	2,3-bisphosph
msc	SCI	SCI	091012_L-02	Calmodulin OS
msc	SCI	SCI	091012_L-02	Histidine triad
msc	SCI	SCI	091012_L-02	Fatty acid-bin
msc	SCI	SCI	091012_L-02	Isoform 3 of D
msc	SCI	SCI	091012_L-02	Isoaspartyl pe
msc	SCI	SCI	091012_L-02	Uncharacteriz
msc	SCI	SCI	091012_L-02	Superoxide di
msc	SCI	SCI	091012_L-02	Isoform 2 of L
msc	SCI	SCI	091012_L-02	Heat shock 70
msc	SCI	SCI	091012_L-02	Tubulin alpha-
msc	SCI	SCI	091012_L-02	Uncharacteriz
msc	SCI	SCI	091012_L-02	Peptidyl-proly
msc	SCI	SCI	091012_L-02	Junctional adh

msc	SCI	SCI	091012_L-02	Thy-1 membra
msc	SCI	SCI	091012_L-02	Heat shock pr
msc	SCI	SCI	091012_L-02	Peroxiredoxin
msc	SCI	SCI	091012_L-02	Sulfated glyco
msc	SCI	SCI	091012_L-02	Isoform Kidne
msc	SCI	SCI	091012_L-02	Branched-cha
msc	SCI	SCI	091012_L-02	Beta-2-microg
msc	SCI	SCI	091012_L-02	Thymosin beta
msc	SCI	SCI	091012_L-02	Isoform 2 of A
msc	SCI	SCI	091012_L-02	Ubiquitin-con
msc				
END OF FILE				

uraj Blasko2, Jan Rosocha3,Timea Spakova3, Christophe Lefebvre1, Isabelle Fournier1, Michel Salz

ot_ref_proteome_112011.fasta

ıase

ants?: Yes

a (Monoisotopic)
(Monoisotopic)
C (Carbamidomethyl)
on M (Oxidation), +42 on n (Acetyl), +80 on Y (Phospho)
s_Uniprot_ref_proteome_112011.fasta (unknown version, 35683 entries)

Cs: Peptide Prophet with Delta Mass Correction [+2 and below,+3,+4,+5,+6,+7 and above]
)2: Peptide Prophet with Delta Mass Correction [+2 and below,+3,+4,+5,+6,+7,+8 and above]
Peptide Prophet with Delta Mass Correction [+2 and below,+3,+4,+5,+6,+7,+8 and above]

01.1)

a (Monoisotopic)
(Monoisotopic)
C (Carbamidomethyl)
ɔn n (Glu->pyro-Glu), -17 on n (Ammonia-loss), -17 on n (Gln->pyro-Glu), -1 on c (Amidated), +16 on I
ʌT database

Cs: Peptide Prophet with Delta Mass Correction [+2 and below,+3,+4 and above]
)2: Peptide Prophet with Delta Mass Correction [+2 and below,+3,+4 and above]
Peptide Prophet with Delta Mass Correction [+2 and below,+3,+4 and above]

tions
affold 4\parameters\unimod.xml

de grouping with binary peptide-protein weights

2 peptides minimum

Protein access	Database sou	Protein molec	Protein identi	Exclusive uniq	Exclusive uniq	Total spectrur
sp|P58775-2|	RAT.fasta	32 958,6	100,0 %	5	5	5
sp|Q5XFX0|T.	RAT.fasta	22 393,8	100,0 %	2	2	3
tr|B2RZA9|B2	RAT.fasta	17 862,3	100,0 %	2	2	2
sp|P62815|V.	RAT.fasta	56 552,3	99,9 %	0	0	0
sp|Q07936|A	RAT.fasta	38 680,2	100,0 %	3	3	10
sp|O88201|C	RAT.fasta	36 387,5	100,0 %	6	6	6
tr|F1LP57|F1I	RAT.fasta	41 102,6	92,2 %	0	0	0
tr|F1LS57|F1I	RAT.fasta	125 961,6	100,0 %	4	4	7
sp|Q6IG05|K.	RAT.fasta	59 027,2	100,0 %	2	2	10
sp|Q00918|L	RAT.fasta	186 590,5	100,0 %	0	0	0
sp|P13697|M	RAT.fasta	64 004,3	36,9 %	0	0	0
tr|Q642B0|Q	RAT.fasta	62 563,0	97,6 %	0	0	0
sp|O35244|P	RAT.fasta	24 819,9	100,0 %	2	2	2
sp|Q6AYC4|C	RAT.fasta	38 799,7	100,0 %	2	2	2
sp|Q5QD51-2	RAT.fasta	181 102,3	99,8 %	0	0	0
tr|D3ZWH5|D	RAT.fasta	28 014,0	21,1 %	0	0	0
sp|P04642|LI	RAT.fasta	36 450,8	100,0 %	4	4	5
tr|D3ZQ25|D.	RAT.fasta	78 070,0	100,0 %	4	4	7
sp|P05539|C(	RAT.fasta	134 572,0	100,0 %	3	3	17
tr|D4A2G6|D	RAT.fasta	129 725,6	100,0 %	22	23	78
sp|Q6Q0N0|(	RAT.fasta	106 259,2	100,0 %	4	4	6
sp|Q810F4|F.	RAT.fasta	24 713,9	100,0 %	2	2	2
tr|Q62669|Q	RAT.fasta	16 022,6	8,5 %	0	0	0
tr|Q3MID6|Q	RAT.fasta	37 148,8	100,0 %	6	9	16
tr|F1MAA7|F	RAT.fasta	177 385,1	100,0 %	1	1	1
sp|P04692-3|	RAT.fasta	32 682,0	98,4 %	0	0	0
sp|P02262|H.	RAT.fasta	14 078,0	100,0 %	4	5	12
sp|Q9JI03|CC	RAT.fasta	183 990,1	100,0 %	28	40	102
sp|Q2PQA9|K	RAT.fasta	109 531,6	99,7 %	0	0	0
sp|Q6P9V9|T	RAT.fasta	50 151,7	100,0 %	2	2	2
sp|P16086|SPTA2_RAT		0,0	100,0 %	0	0	0
tr|D3ZPA9|D3ZPA9_RAT		0,0	100,0 %	4	4	8
tr|D3ZEI4|D3	RAT.fasta	46 530,2	100,0 %	0	0	0
sp|P63029|T(	RAT.fasta	19 462,9	9,9 %	0	0	0
tr|Q5PQU1|Q5PQU1_RAT		0,0	9,9 %	0	0	0
sp|Q99PW7|I	RAT.fasta	27 107,4	100,0 %	4	4	4
tr|F1LS40|F1I	RAT.fasta	129 838,5	100,0 %	100	136	1002
sp|Q5GFD9|II	RAT.fasta	35 995,6	94,6 %	0	0	0
tr|D3ZQN7|D	RAT.fasta	202 784,2	100,0 %	2	2	2
tr|Q5XI38|Q5	RAT.fasta	70 124,7	99,7 %	0	0	0
tr|D4ABR6|D.	RAT.fasta	0,0	99,8 %	0	0	0
sp|Q01129|P	RAT.fasta	39 806,8	100,0 %	1	1	1
sp|Q7M0E3|I	RAT.fasta	18 534,1	100,0 %	4	4	5
tr|Q9JI04|Q9.	RAT.fasta	171 575,7	100,0 %	3	3	3

tr|F1M983|F:	RAT.fasta	140 007,0	7,9 %	0	0	0
sp|P21575-3|	RAT.fasta	92 378,1	99,0 %	0	0	0
tr|D3ZQR7|D.	RAT.fasta	87 014,0	100,0 %	14	14	18
tr|F8WGA3|F	RAT.fasta	42 336,0	96,1 %	0	0	0
tr|A7M777|A7M777_RAT		0,0	100,0 %	5	5	5
sp|P04639|Al	RAT.fasta	30 062,4	8,9 %	0	0	0
sp|Q9ERB4-2	RAT.fasta	300 004,9	24,9 %	0	0	0
sp|P68255|14	RAT.fasta	27 779,4	100,0 %	2	2	3
sp|P07151|B2	RAT.fasta	13 720,0	100,0 %	4	6	8
sp|Q9R0J8|L(	RAT.fasta	49 465,8	99,9 %	0	0	0
sp|P62738|A(	RAT.fasta	42 010,1	100,0 %	6	7	10
tr|D4ADG9|D	RAT.fasta	66 931,9	100,0 %	4	4	4
tr|F1LUQ1|F1LUQ1_RAT		0,0	100,0 %	5	5	5
sp|P31044|PI	RAT.fasta	20 801,4	95,0 %	1	1	1
tr|F1M9V7|F:	RAT.fasta	103 347,0	100,0 %	0	0	0
tr|F1M7P4|F:	RAT.fasta	53 983,3	24,9 %	0	0	0
sp|P11598|PI	RAT.fasta	56 625,5	92,0 %	0	0	0
sp|Q9WVH8|	RAT.fasta	50 159,8	100,0 %	1	1	2
sp|Q5XII0|EP	RAT.fasta	25 639,5	100,0 %	4	4	5
sp|Q63416|IT	RAT.fasta	99 099,5	100,0 %	1	1	2
sp|P16975|SI	RAT.fasta	34 295,7	100,0 %	16	25	155
sp|O35793|G	RAT.fasta	20 680,0	100,0 %	1	1	2
sp|P97697|IN	RAT.fasta	30 511,6	95,8 %	0	0	0
sp|P82995|H:	RAT.fasta	84 818,3	100,0 %	2	2	2
sp|P20961|P/	RAT.fasta	45 010,6	100,0 %	20	29	68
sp|P30121|TI	RAT.fasta	24 357,0	100,0 %	11	14	29
tr|Q6IFV6|Q6	RAT.fasta	50 675,4	100,0 %	5	5	5
tr|D4A7Y1|D4	RAT.fasta	224 612,0	100,0 %	8	9	15
tr|B5DF94|B5DF94_RAT,tr|		0,0	100,0 %	2	2	7
sp|P09812|P\	RAT.fasta	97 276,7	93,3 %	0	0	0
sp|P08721|O.	RAT.fasta	34 962,7	100,0 %	4	4	5
tr|D3ZUK7|D3ZUK7_RAT		0,0	9,4 %	0	0	0
tr|D3ZRK9|D3	RAT.fasta	0,0	99,9 %	0	0	0
tr|D3ZJ08|D3	RAT.fasta	15 388,7	100,0 %	2	2	2
sp|Q08163|C	RAT.fasta	51 588,9	15,6 %	0	0	0
sp|P19332-2|	RAT.fasta	71 773,6	79,1 %	0	0	0
sp|P36953|Al	RAT.fasta	69 336,5	99,5 %	0	0	0
sp|Q63544|S'	RAT.fasta	12 975,3	31,7 %	0	0	0
sp|P00697|LY	RAT.fasta	16 729,2	100,0 %	3	3	6
sp|P02091|H	RAT.fasta	15 979,4	100,0 %	2	2	10
sp|Q99MZ8|l	RAT.fasta	29 969,9	100,0 %	2	2	2
tr|D4AC70|D4	RAT.fasta	73 452,3	100,0 %	2	2	2
sp|P10860|DI	RAT.fasta	61 417,4	98,8 %	0	0	0
sp|Q9R0D6|T	RAT.fasta	47 421,5	100,0 %	4	4	4
sp|Q6P7Q4|L	RAT.fasta	20 820,7	99,2 %	0	0	0
sp|P15999|AT	RAT.fasta	59 755,2	100,0 %	1	1	1
sp|P06238|A:	RAT.fasta	163 785,7	100,0 %	2	3	6
tr|F1LPS8|F1I	RAT.fasta	33 737,1	43,9 %	0	0	0
sp|P36972|Al	RAT.fasta	19 546,6	100,0 %	2	2	2
sp|Q9JHU0|D	RAT.fasta	61 539,8	98,2 %	0	0	0
tr|F1M798|F:	RAT.fasta	111 649,0	100,0 %	2	2	2

sp|P62161|C/	RAT.fasta	16 838,0	100,0 %	3	3	4
sp|P17475|A:	RAT.fasta	46 137,6	99,7 %	0	0	0
tr|F1MA59|F:	RAT.fasta	160 614,4	100,0 %	4	4	4
sp|P60711|A(	RAT.fasta	41 737,8	100,0 %	18	33	105
sp|P11980|KI	RAT.fasta	57 818,6	100,0 %	3	3	6
sp|Q00715|H	RAT.fasta	13 990,6	100,0 %	3	4	13
sp|Q9JK11|R'	RAT.fasta	126 391,0	100,0 %	0	0	0
sp|P55068|P(	RAT.fasta	96 057,1	97,9 %	0	0	0
tr|F1M9B2|F:	RAT.fasta	28 948,7	100,0 %	12	14	34
sp|P47709|RI	RAT.fasta	75 833,3	100,0 %	0	0	0
sp|P31000|VI	RAT.fasta	53 733,9	100,0 %	23	26	52
tr|F1LM30|F1	RAT.fasta	51 745,4	89,6 %	0	0	0
sp|Q07439|H	RAT.fasta	70 186,8	100,0 %	2	2	2
tr|B2RYM3|B	RAT.fasta	100 589,7	15,0 %	0	0	0
sp|P07722|M	RAT.fasta	69 352,9	48,1 %	0	0	0
sp|P16636|LY	RAT.fasta	46 558,9	100,0 %	2	2	2
tr|F1M566|F:	RAT.fasta	230 834,3	100,0 %	5	5	5
sp|P02650|AI	RAT.fasta	35 753,4	100,0 %	5	5	6
tr|F1LR02|F1	RAT.fasta	134 646,3	99,4 %	0	0	0
tr|B0K010|BC	RAT.fasta	14 091,9	98,6 %	0	0	0
sp|P02454|C(	RAT.fasta	137 953,8	100,0 %	98	147	996
sp|P04764|EI	RAT.fasta	47 129,0	100,0 %	8	9	14
tr|F1M8G9|F	RAT.fasta	91 166,5	97,5 %	0	0	0
sp|Q07257-2|	RAT.fasta	50 535,3	100,0 %	4	4	5
sp|Q68FP1-2|	RAT.fasta	86 067,9	99,4 %	1	1	1
sp|P10719|A'	RAT.fasta	56 354,3	100,0 %	3	3	3
sp|P0CG51|U	RAT.fasta	14 728,9	100,0 %	2	2	8
sp|Q03626|M	RAT.fasta	165 327,3	93,5 %	0	0	0
sp|P05544|SI	RAT.fasta	46 279,0	30,1 %	0	0	0
sp|Q6IG01|K:	RAT.fasta	57 256,1	100,0 %	2	2	2
sp|P35053|G	RAT.fasta	61 735,5	100,0 %	2	2	3
tr|Q5RJR9|Q!	RAT.fasta	46 563,0	100,0 %	3	4	4
sp|Q64559-1|	RAT.fasta	37 560,3	17,5 %	0	0	0
sp|P34058|H!	RAT.fasta	83 284,3	100,0 %	5	5	5
sp|P05708|H:	RAT.fasta	102 411,0	99,8 %	0	0	0
sp|Q6P0K8|P	RAT.fasta	81 801,9	100,0 %	8	11	15
tr|F1LM42|F1	RAT.fasta	434 346,5	100,0 %	0	0	0
sp|P85972|VI	RAT.fasta	116 617,3	100,0 %	5	6	7
sp|P11442|CI	RAT.fasta	0,0	100,0 %	1	1	1
sp|P05982|N(	RAT.fasta	30 947,5	100,0 %	5	6	6
sp|P21961|CBPA3_RAT-DE(		0,0	99,8 %	0	0	0
sp|Q6P7B4|F	RAT.fasta	58 207,2	100,0 %	0	0	0
tr|D3ZP82|D:	RAT.fasta	83 627,0	100,0 %	6	7	9
sp|Q6IFU7|K:	RAT.fasta	50 214,0	100,0 %	3	3	6
tr|C0JPT7|C0.	RAT.fasta	280 485,3	100,0 %	4	4	4
sp|P63329-2|	RAT.fasta	58 645,7	99,4 %	0	0	0
tr|F8WG88|F	RAT.fasta	60 600,9	100,0 %	16	22	54
sp|Q63691|C	RAT.fasta	40 055,2	15,7 %	0	0	0
sp|P09606|G	RAT.fasta	42 268,3	34,9 %	0	0	0
sp|P24268|C/	RAT.fasta	44 682,0	100,0 %	6	9	11
sp|Q4FZU2|K	RAT.fasta	59 250,6	100,0 %	3	3	27

sp|Q9Z1P2|A	RAT.fasta	102 963,9	100,0 %	7	7	8
sp|P09006|SI	RAT.fasta	46 654,0	27,2 %	0	0	0
sp|Q05982|N	RAT.fasta	17 193,0	100,0 %	6	7	10
tr|D3ZK12|D3ZK12_RAT		0,0	100,0 %	8	8	12
tr|D3ZFH5|D:	RAT.fasta	106 528,4	100,0 %	3	3	7
tr|F1LRX5|F1LRX5_RAT		0,0	100,0 %	0	0	0
sp|P31722|C:	RAT.fasta	25 685,9	27,9 %	0	0	0
sp|O08628|P	RAT.fasta	50 185,1	100,0 %	16	18	44
sp|P39069|K/	RAT.fasta	21 584,4	99,6 %	0	0	0
tr|E9PSV5|E9	RAT.fasta	40 490,9	100,0 %	0	0	0
sp|P07895|S(	RAT.fasta	24 674,3	99,5 %	0	0	0
sp|P13941|C(	RAT.fasta	138 936,9	100,0 %	54	76	337
tr|F1LYE8|F1LYE8_RAT		0,0	100,0 %	0	0	0
tr|D3ZFC6|D3	RAT.fasta	103 757,3	92,4 %	0	0	0
tr|E9PSN4|E9	RAT.fasta	203 528,4	100,0 %	2	2	2
sp|P10111|PI	RAT.fasta	17 874,8	100,0 %	5	6	16
sp|P19944|RI	RAT.fasta	11 498,1	95,0 %	1	1	1
sp|P13221|A/	RAT.fasta	46 429,5	13,8 %	0	0	0
sp|P04937|FI	RAT.fasta	272 503,7	91,8 %	1	1	2
tr|D3ZQM5|D3ZQM5_RAT		0,0	100,0 %	5	5	7
sp|P85971|6I	RAT.fasta	27 235,0	100,0 %	2	2	2
sp|P17246|T(	RAT.fasta	44 330,0	100,0 %	3	3	3
tr|Q5I0M1|Q	RAT.fasta	38 457,3	28,6 %	0	0	0
tr|D3Z8P5|D3	RAT.fasta	35 269,8	99,7 %	0	0	0
sp|P35704|PI	RAT.fasta	21 784,1	99,8 %	1	1	1
sp|Q4QRB4|T	RAT.fasta	50 418,7	95,0 %	1	1	1
sp|P01026|C(	RAT.fasta	186 462,2	100,0 %	5	5	9
sp|Q63772|G	RAT.fasta	74 637,2	99,6 %	1	1	1
sp|P14480|FI	RAT.fasta	54 235,8	97,7 %	0	0	0
sp|P62804|H	RAT.fasta	11 367,7	100,0 %	5	6	12
tr|D3ZVB7|D3	RAT.fasta	34 071,5	100,0 %	2	2	2
sp|P04785|PI	RAT.fasta	56 953,4	100,0 %	1	1	1
sp|P02680-2|	RAT.fasta	50 634,3	63,6 %	0	0	0
sp|Q9JI85|NU	RAT.fasta	50 091,2	100,0 %	5	8	14
sp|Q01177|P	RAT.fasta	90 535,1	100,0 %	1	1	1
sp|P10960|S/	RAT.fasta	61 122,8	100,0 %	6	6	6
tr|Q6AYQ9|Q	RAT.fasta	23 009,6	100,0 %	5	8	9
sp|Q5RJP7|P(	RAT.fasta	41 932,9	100,0 %	4	4	4
sp|Q6IG00|K:	RAT.fasta	57 668,2	5,1 %	0	0	0
tr|Q9ES33|Q!	RAT.fasta	30 762,0	100,0 %	7	11	26
sp|Q9R1E9|C	RAT.fasta	37 755,2	100,0 %	11	11	29
sp|Q63768-2|	RAT.fasta	33 844,8	9,8 %	0	0	0
sp|P25113|P(	RAT.fasta	28 832,8	100,0 %	4	4	4
sp|Q63560|M	RAT.fasta	100 484,2	100,0 %	0	0	0
sp|Q6AY84|S	RAT.fasta	46 396,0	21,3 %	0	0	0
sp|Q9WUW3	RAT.fasta	67 296,6	8,1 %	0	0	0
sp|P51635|AI	RAT.fasta	36 506,2	100,0 %	4	4	9
tr|Q9QX80|Q	RAT.fasta	30 853,6	16,9 %	0	0	0
tr|F1LRZ7|F1I	RAT.fasta	114 411,8	100,0 %	2	2	17
sp|Q6P6Q2|K	RAT.fasta	61 827,3	100,0 %	19	23	110
sp|P35213|1	RAT.fasta	28 055,2	100,0 %	1	1	2

tr|D3ZDF6|D3ZDF6_RAT		0,0	100,0 %	2	2	2
tr|F1LM84|F1	RAT.fasta	137 037,9	100,0 %	17	17	27
sp|P33436|M	RAT.fasta	74 151,7	100,0 %	24	28	59
tr|F1LMV6|F1	RAT.fasta	332 400,6	100,0 %	24	27	43
sp|Q9WV75|S	RAT.fasta	36 014,6	100,0 %	2	2	3
sp|P12839|N	RAT.fasta	95 790,7	100,0 %	0	0	0
tr|F1LUV9|F1	RAT.fasta	92 311,9	99,3 %	0	0	0
sp|Q6P6V0|G	RAT.fasta	62 829,3	100,0 %	0	0	0
tr|F1LUH9|F1LUH9_RAT		0,0	100,0 %	3	3	3
sp|P48679|LM	RAT.fasta	74 325,0	100,0 %	5	5	6
sp|P97685-3|	RAT.fasta	132 181,4	99,8 %	0	0	0
sp|Q9EPB1|D	RAT.fasta	55 115,3	100,0 %	2	2	5
tr|D3ZM43|D3ZM43_RAT		0,0	99,9 %	0	0	0
tr|F1LNH3|F1	RAT.fasta	109 660,6	100,0 %	4	4	4
sp|P45592|C(	RAT.fasta	18 533,2	100,0 %	6	6	6
sp|P09951-2|	RAT.fasta	73 988,3	7,6 %	0	0	0
sp|Q5XIF6|TE	RAT.fasta	49 924,6	95,0 %	1	1	1
sp|Q62930|C	RAT.fasta	62 282,5	99,5 %	0	0	0
sp|P02688-2|	RAT.fasta	18 488,3	100,0 %	2	2	4
sp|P14141|CA	RAT.fasta	29 431,8	100,0 %	0	0	0
sp|O35276|N	RAT.fasta	103 899,5	100,0 %	2	2	2
sp|Q6IFU8|K1	RAT.fasta	48 123,9	100,0 %	6	7	24
sp|Q63041|A	RAT.fasta	167 126,0	100,0 %	1	1	1
tr|D3ZVQ0|D	RAT.fasta	95 780,8	99,3 %	0	0	0
sp|P47819|G	RAT.fasta	49 957,9	100,0 %	1	1	23
sp|P50399|G	RAT.fasta	50 539,1	100,0 %	5	5	5
tr|B2GV03|B2GV03_RAT		0,0	100,0 %	0	0	0
sp|P61983|14	RAT.fasta	28 303,1	100,0 %	2	2	4
sp|Q07258|T	RAT.fasta	47 116,4	100,0 %	2	2	2
sp|P07335|K(	RAT.fasta	42 726,1	25,9 %	0	0	0
tr|D3ZQP6|D3	RAT.fasta	75 143,5	100,0 %	1	1	1
sp|A2RUV9|A	RAT.fasta	128 063,7	100,0 %	6	6	12
sp|P07632|S(	RAT.fasta	15 910,7	100,0 %	4	4	7
tr|F8WFW0|F8WFW0_RAT		0,0	99,8 %	0	0	0
sp|P03994|H	RAT.fasta	40 262,4	99,7 %	0	0	0
sp|P04905|G	RAT.fasta	25 915,9	96,2 %	0	0	0
sp|Q00959|NMDE1_RAT-DE		0,0	99,8 %	0	0	0
sp|P02770|AL	RAT.fasta	68 731,2	100,0 %	3	5	32
tr|Q8CHN5|C	RAT.fasta	16 363,9	100,0 %	5	6	9
sp|Q5XI73|GI	RAT.fasta	23 408,0	100,0 %	5	6	7
sp|P24368|PI	RAT.fasta	23 802,9	100,0 %	8	9	9
sp|Q9WUQ1|	RAT.fasta	105 705,8	100,0 %	3	3	3
tr|Q6IN22|Q6	RAT.fasta	37 544,1	100,0 %	15	19	49
tr|D3ZQ74|D3	RAT.fasta	83 615,3	100,0 %	1	1	1
tr|E9PSN2|E9	RAT.fasta	0,0	100,0 %	1	1	1
tr|F1LRL9|F1L	RAT.fasta	269 643,4	100,0 %	0	0	0
sp|P11232|TH	RAT.fasta	11 673,3	100,0 %	2	2	4
sp|Q5RKI0|W	RAT.fasta	66 181,1	81,8 %	0	0	0
sp|P60203|M	RAT.fasta	30 077,7	100,0 %	3	3	4
sp|O88989|M	RAT.fasta	36 483,9	100,0 %	4	4	4
tr|B1WC91|B	RAT.fasta	35 043,1	100,0 %	6	8	21

tr|D3ZZ86|D3	RAT.fasta	0,0	100,0 %	2	2	2
sp|P04797|G	RAT.fasta	35 828,1	100,0 %	0	0	0
sp|P85515|A	RAT.fasta	42 615,2	97,5 %	0	0	0
sp|Q63945|S	RAT.fasta	33 406,4	99,1 %	0	0	0
sp|P02767|T	RAT.fasta	15 719,9	94,3 %	0	0	0
sp|P04276|V	RAT.fasta	53 545,6	99,9 %	0	0	0
tr|D3ZTX4|D3ZTX4_RAT-DE		0,0	99,8 %	0	0	0
sp|P60881|S	RAT.fasta	23 315,4	13,0 %	0	0	0
sp|Q64361|L	RAT.fasta	25 579,1	95,0 %	1	1	1
sp|P23593|A	RAT.fasta	21 635,8	19,7 %	0	0	0
tr|Q3MIE4|Q3MIE4_RAT		0,0	100,0 %	4	4	4
sp|Q6IMF3|K	RAT.fasta	64 831,7	100,0 %	6	9	69
sp|P06686|A	RAT.fasta	112 220,7	91,8 %	0	0	0
sp|P52303-2|	RAT.fasta	104 591,4	5,1 %	0	0	0
sp|Q6IFV1|K	RAT.fasta	52 684,9	100,0 %	10	12	48
tr|D3ZXM9|D	RAT.fasta	131 885,3	99,1 %	0	0	0
tr|B2RZ27|B2	RAT.fasta	10 476,8	95,0 %	1	1	1
sp|Q63716|P	RAT.fasta	22 110,2	100,0 %	4	4	5
sp|P47942|D	RAT.fasta	62 277,9	98,9 %	0	0	0
sp|Q64240|A	RAT.fasta	38 851,4	99,7 %	0	0	0
tr|Q3MHS9|C	RAT.fasta	58 018,4	27,2 %	0	0	0
sp|P47875|C	RAT.fasta	20 613,2	100,0 %	5	6	6
tr|D3ZTB5|D3	RAT.fasta	11 198,3	18,0 %	0	0	0
sp|P20059|H	RAT.fasta	51 351,5	98,3 %	0	0	0
sp|Q63434|P	RAT.fasta	17 681,3	100,0 %	2	2	2
sp|Q9ESM2|H	RAT.fasta	38 047,2	32,2 %	0	0	0
sp|P34926|M	RAT.fasta	299 530,8	96,3 %	0	0	0
sp|P70490|M	RAT.fasta	47 413,4	100,0 %	15	17	33
sp|Q5FVH0|C	RAT.fasta	25 334,7	100,0 %	3	3	3
sp|P47853|P	RAT.fasta	41 708,6	100,0 %	15	22	89
tr|F1LTF8|F1	RAT.fasta	195 626,6	100,0 %	2	2	2
sp|P14046|A	RAT.fasta	163 774,4	99,9 %	1	1	5
sp|Q63202|A	RAT.fasta	82 310,9	98,4 %	0	0	0
sp|Q9EPC6|P	RAT.fasta	15 002,1	94,3 %	0	0	0
sp|Q6P9T8|TBB2C_RAT		0,0	99,6 %	1	1	1
sp|Q9QUL6|N	RAT.fasta	82 655,5	97,3 %	0	0	0
sp|Q6IG02|K	RAT.fasta	69 127,7	100,0 %	8	10	50
sp|P63018|H	RAT.fasta	70 872,8	100,0 %	2	2	2
sp|P47728|C	RAT.fasta	31 406,7	16,0 %	0	0	0
sp|P35745|A	RAT.fasta	10 863,2	19,3 %	0	0	0
sp|P07154|C	RAT.fasta	37 660,6	100,0 %	7	8	12
tr|A7M778|A	RAT.fasta	53 484,3	100,0 %	12	13	20
sp|P42123|L	RAT.fasta	36 612,5	100,0 %	1	1	2
sp|P20909|C	RAT.fasta	181 029,5	100,0 %	6	6	6
sp|P05065|A	RAT.fasta	39 352,6	100,0 %	2	2	2
sp|P51886|L	RAT.fasta	38 281,3	100,0 %	11	14	46
tr|F1LP60|F1	RAT.fasta	67 653,6	100,0 %	7	8	8
tr|D3ZUL3|D3	RAT.fasta	108 807,3	100,0 %	9	9	13
tr|D3ZY51|D3	RAT.fasta	67 218,5	100,0 %	3	3	5
tr|F1LQI1|F1	RAT.fasta	34 157,9	7,7 %	0	0	0
sp|P06866|H	RAT.fasta	38 563,0	13,0 %	0	0	0

tr|F1LST1|F1I	RAT.fasta	202 546,0	100,0 %	96	137	454
tr|D3Z9F8|D3	RAT.fasta	212 445,0	100,0 %	69	81	123
tr|D4A6X4|D4	RAT.fasta	11 311,9	85,5 %	0	0	0
sp|P06399|FI	RAT.fasta	86 687,8	96,6 %	0	0	0
sp|P04904|G:	RAT.fasta	25 320,4	43,9 %	0	0	0
tr|F1LPM2|F1LPM2_RAT		0,0	100,0 %	2	2	2
sp|Q63083|N	RAT.fasta	53 506,9	100,0 %	10	10	15
sp|P08494|M	RAT.fasta	12 037,1	100,0 %	1	1	3
sp|P46462|TE	RAT.fasta	89 351,8	100,0 %	2	2	2
sp|P62260|14	RAT.fasta	29 175,0	100,0 %	3	3	4
sp|P85970|AI	RAT.fasta	34 391,8	100,0 %	2	2	2
tr|D3ZFY8|D3	RAT.fasta	16 354,8	97,7 %	0	0	0
tr|F1LPD0|F1	RAT.fasta	0,0	88,7 %	0	0	0
tr|D3ZUM4|D	RAT.fasta	73 231,2	100,0 %	1	1	1
sp|P19804|N	RAT.fasta	17 283,3	100,0 %	3	3	3
sp|Q6IFW6|K	RAT.fasta	56 506,5	100,0 %	20	27	183
sp|B0BND0|E	RAT.fasta	50 702,6	6,2 %	0	0	0
sp|Q5FVJ0-2|	RAT.fasta	52 909,0	100,0 %	0	0	0
sp|B2RYG6|O	RAT.fasta	31 270,5	62,2 %	1	1	1
sp|P24090|FE	RAT.fasta	37 981,1	99,4 %	0	0	0
sp|P04636|M	RAT.fasta	35 684,2	100,0 %	3	3	4
sp|Q63532|SI	RAT.fasta	16 731,5	100,0 %	2	2	3
sp|P11030|A(	RAT.fasta	10 027,9	95,8 %	0	0	0
sp|Q6MGD0|	RAT.fasta	18 658,7	90,7 %	0	0	0
sp|Q08420|S(	RAT.fasta	26 619,3	100,0 %	2	3	5
tr|Q5M7T5|C	RAT.fasta	52 235,3	100,0 %	1	1	2
tr|F1MAF7|F1	RAT.fasta	46 837,1	100,0 %	10	11	12
tr|D3ZWH0|D3ZWH0_RAT-I		0,0	100,0 %	2	2	2
tr|F1LSW0|F1LSW0_RAT		0,0	100,0 %	15	16	58
sp|P01830|TI	RAT.fasta	18 172,8	100,0 %	1	1	1
sp|B0BNI5|OI	RAT.fasta	45 962,3	99,9 %	1	1	1
sp|P18331|IN	RAT.fasta	47 406,9	100,0 %	12	12	13
sp|Q920J4|T)	RAT.fasta	32 249,5	31,3 %	0	0	0
sp|Q6AY61|P	RAT.fasta	43 161,1	100,0 %	3	3	3
tr|Q9JKB7|Q9	RAT.fasta	50 901,6	100,0 %	1	1	1
sp|P97546-3|	RAT.fasta	43 932,2	100,0 %	2	2	2
sp|Q64537|C	RAT.fasta	28 571,1	99,2 %	0	0	0
sp|O88600|H	RAT.fasta	94 057,4	100,0 %	1	1	1
sp|P14668|AI	RAT.fasta	35 746,5	99,9 %	0	0	0
sp|P05371|CI	RAT.fasta	51 375,6	100,0 %	2	2	2
tr|F1MAG6|F1MAG6_RAT		0,0	100,0 %	0	0	0
sp|P07943|AI	RAT.fasta	35 797,7	100,0 %	4	4	4
sp|Q5PPN5|T	RAT.fasta	18 980,2	27,7 %	0	0	0
sp|P07150|AI	RAT.fasta	38 831,0	100,0 %	2	2	3
sp|P00762|TF	RAT.fasta	25 959,1	100,0 %	5	6	122
sp|P13233|CI	RAT.fasta	47 270,0	99,6 %	0	0	0
sp|P23785|G	RAT.fasta	63 366,6	100,0 %	3	3	5
tr|D4A111|D4	RAT.fasta	306 150,3	96,0 %	0	0	0
tr|D3ZF59|D3ZF59_RAT		0,0	100,0 %	2	2	2
sp|P48500|TF	RAT.fasta	26 848,7	100,0 %	6	6	9
sp|P62963|PI	RAT.fasta	14 957,3	100,0 %	8	11	18

sp|Q62611-2|	RAT.fasta	64 406,5	100,0 %	5	5	5
sp|P07936|N	RAT.fasta	23 603,2	99,1 %	0	0	0
sp|P50137|TI	RAT.fasta	67 644,7	100,0 %	0	0	0
sp|P62630|EI	RAT.fasta	50 114,2	100,0 %	6	7	15
sp|P12346|TI	RAT.fasta	76 395,6	100,0 %	4	5	6
tr|Q6IFZ5|Q6	RAT.fasta	61 759,6	100,0 %	2	2	3
sp|Q6P7S1|A	RAT.fasta	44 444,6	75,8 %	0	0	0
tr|F1LNN9|F1LNN9_RAT		0,0	100,0 %	2	2	3
sp|P19527|N	RAT.fasta	61 336,5	43,9 %	0	0	0
sp|P14841|C'	RAT.fasta	15 436,8	100,0 %	5	7	25
tr|Q5U2V1|Q	RAT.fasta	64 788,7	100,0 %	8	9	9
sp|P11762|LE	RAT.fasta	14 857,2	100,0 %	6	7	15
tr|D3ZHA0|D:	RAT.fasta	290 978,7	99,9 %	0	0	0
sp|Q5XI43|M	RAT.fasta	42 734,8	100,0 %	4	4	4
sp|P63102|14	RAT.fasta	27 771,9	100,0 %	12	12	23
sp|Q2Q0I9|FI	RAT.fasta	194 048,8	100,0 %	9	10	11
tr|D4A0Y1|D4A0Y1_RAT		0,0	100,0 %	6	6	12
sp|Q78P75|D	RAT.fasta	10 350,1	27,0 %	0	0	0
sp|Q9ER34|A	RAT.fasta	85 436,1	99,8 %	0	0	0
sp|P08413|K(	RAT.fasta	60 402,3	99,3 %	0	0	0
tr|F1M9K9|F:	RAT.fasta	120 417,4	55,2 %	0	0	0
sp|P05197|EI	RAT.fasta	95 286,0	100,0 %	2	2	3
tr|F1LRR0|F1LRR0_RAT,tr|F		0,0	100,0 %	25	33	93
tr|D4A856|D4A856_RAT		0,0	15,6 %	0	0	0
tr|D4A115|D4	RAT.fasta	240 199,7	100,0 %	7	7	9
sp|Q9JLJ3|AL	RAT.fasta	53 653,2	94,3 %	0	0	0
tr|E9PSV0|E9PSV0_RAT		0,0	100,0 %	2	2	2
sp|P70564|SI	RAT.fasta	42 065,2	100,0 %	2	2	2
tr|D3ZZX3|D3ZZX3_RAT		0,0	96,7 %	0	0	0
sp|P20760|IG	RAT.fasta	35 184,7	12,6 %	0	0	0
tr|Q9QZK5|Q9QZK5_RAT		0,0	100,0 %	1	1	1
sp|Q9QZQ5|I	RAT.fasta	38 508,5	100,0 %	7	7	15
tr|D4A5L9|D4	RAT.fasta	11 636,0	100,0 %	0	0	0
tr|D3ZD09|D:	RAT.fasta	10 071,5	84,9 %	0	0	0
sp|Q00981|U	RAT.fasta	24 838,2	98,6 %	0	0	0
sp|Q6QD51|(	RAT.fasta	107 693,2	100,0 %	4	4	6
sp|P85973|PI	RAT.fasta	32 301,7	100,0 %	6	6	6
tr|F1M6Q3|F	RAT.fasta	166 252,0	100,0 %	1	1	1
sp|Q9R063|P	RAT.fasta	22 178,5	100,0 %	1	1	1
sp|P30904|M	RAT.fasta	12 477,4	100,0 %	3	4	4
sp|Q9QXQ0|/	RAT.fasta	104 918,2	100,0 %	11	11	20
tr|Q6P6G4|Q	RAT.fasta	30 077,3	40,0 %	0	0	0
sp|Q5XI32|C/	RAT.fasta	30 629,7	100,0 %	1	1	1
sp|P85108|TE	RAT.fasta	49 907,1	100,0 %	3	3	3
tr|F1LXC0|F1	RAT.fasta	198 340,6	8,2 %	0	0	0
sp|P30120|TI	RAT.fasta	23 794,2	100,0 %	10	16	41
sp|P10760|S/	RAT.fasta	47 538,9	99,7 %	0	0	0
sp|Q68FS4-2|	RAT.fasta	56 151,2	100,0 %	1	1	1
tr|F1M8K0|F:	RAT.fasta	96 707,0	100,0 %	2	2	3
tr|B2RZ72|B2	RAT.fasta	19 667,4	100,0 %	2	2	2
sp|P80254|D(	RAT.fasta	13 133,9	100,0 %	3	3	3

sp|P01946|H	RAT.fasta	15 328,7	100,0 %	2	3	3
tr|Q7TP54|Q	RAT.fasta	144 712,1	100,0 %	5	9	22
sp|P09117|Al	RAT.fasta	39 284,3	96,8 %	0	0	0
sp|P06761|G	RAT.fasta	72 348,5	100,0 %	1	1	1
sp|Q6PTT0|A	RAT.fasta	45 822,2	9,2 %	0	0	0
tr|F1LQ00|F1	RAT.fasta	142 475,8	100,0 %	46	63	198
tr|E9PSI7|E9I	RAT.fasta	57 166,5	100,0 %	7	10	12
sp|P31232|T/	RAT.fasta	22 602,7	100,0 %	16	22	51
tr|F1MAL6|F:	RAT.fasta	518 261,9	100,0 %	2	2	2
sp|P25304-2|	RAT.fasta	208 638,9	21,9 %	0	0	0
tr|Q5XI84|Q5	RAT.fasta	44 849,2	95,0 %	1	1	1
sp|Q6IG03|K:	RAT.fasta	60 388,4	100,0 %	3	3	37
sp|P55053|F/	RAT.fasta	15 059,3	57,5 %	0	0	0
tr|F1M335|F:	RAT.fasta	164 854,5	100,0 %	2	2	2
tr|F1LV50|F1	RAT.fasta	34 405,7	100,0 %	2	2	2
sp|Q6IFV4|K1	RAT.fasta	47 730,8	100,0 %	2	3	3
tr|Q6IFU9|Q6	RAT.fasta	50 777,8	100,0 %	4	6	7
tr|D3ZAF5|D3	RAT.fasta	90 059,4	100,0 %	39	56	240
sp|Q6B345|S	RAT.fasta	11 065,2	100,0 %	4	6	9
sp|O55004|R	RAT.fasta	16 903,2	100,0 %	4	4	5
sp|Q64119|M	RAT.fasta	16 974,9	100,0 %	1	1	2
sp|P08009|G	RAT.fasta	25 682,7	100,0 %	1	1	1
tr|Q66HI5|Q6	RAT.fasta	21 100,1	5,8 %	0	0	0
sp|P02466|C(	RAT.fasta	129 564,9	100,0 %	3	3	4
sp|P04906|G	RAT.fasta	23 439,8	100,0 %	4	4	4
sp|Q62812|M	RAT.fasta	226 343,8	100,0 %	1	1	1
sp|P07323|El	RAT.fasta	47 141,5	87,3 %	0	0	0
tr|F1M853|F:	RAT.fasta	170 987,4	100,0 %	0	0	0
sp|Q9QX79|F	RAT.fasta	41 532,1	14,2 %	0	0	0
sp|P50398|G	RAT.fasta	50 538,2	48,1 %	0	0	0
tr|D4A5L9|D4	RAT.fasta	11 636,0	87,3 %	0	0	0
tr|F1LRX5|F1LRX5_RAT		0,0	97,2 %	0	0	0
sp|Q6IG00|K:	RAT.fasta	57 668,2	100,0 %	2	2	2
tr|F1M853|F:	RAT.fasta	170 987,4	6,6 %	0	0	0
sp|Q6P7B4|F	RAT.fasta	58 207,2	100,0 %	3	3	3
tr|D3ZUM4|D	RAT.fasta	73 231,2	100,0 %	2	2	2
sp|P21961|CBPA3_RAT-DE(		0,0	100,0 %	2	2	2
tr|D3ZD09|D:	RAT.fasta	10 071,5	8,8 %	0	0	0
tr|D3ZQ74|D:	RAT.fasta	83 615,3	100,0 %	2	2	2
sp|P04797|G	RAT.fasta	35 828,1	100,0 %	1	1	1
tr|D3ZTX4|D3ZTX4_RAT-DE		0,0	100,0 %	2	2	2
tr|D3ZXM9|D	RAT.fasta	131 885,3	97,5 %	1	1	1
sp|P04937|FI	RAT.fasta	272 503,7	100,0 %	2	2	4
tr|F1LXC0|F1	RAT.fasta	198 340,6	100,0 %	2	2	2
sp|P04904|G	RAT.fasta	25 320,4	99,5 %	1	1	1
sp|Q01177|P	RAT.fasta	90 535,1	99,5 %	1	1	1
sp|Q00959|NMDE1_RAT-DI		0,0	100,0 %	2	2	2
sp|Q63202|A	RAT.fasta	82 310,9	100,0 %	2	2	2
sp|P20760|IG	RAT.fasta	35 184,7	85,5 %	0	0	0
sp|P11442|Cl	RAT.fasta	0,0	40,9 %	0	0	0
sp|Q6P6V0|G	RAT.fasta	62 829,3	100,0 %	2	2	3

tr|Q9JKB7|Q9	RAT.fasta	50 901,6	97,5 %	1	1	1
sp|Q68FP1-2|	RAT.fasta	86 067,9	99,5 %	1	1	1
tr|D3Z8P5|D3	RAT.fasta	35 269,8	73,2 %	0	0	0
sp|B0BNI5|O	RAT.fasta	45 962,3	100,0 %	2	2	3
tr|B2GV03|B2GV03_RAT		0,0	22,3 %	0	0	0
tr|F1M6Q3|F	RAT.fasta	166 252,0	100,0 %	2	2	3
tr|Q9QX80|Q	RAT.fasta	30 853,6	7,3 %	0	0	0
sp|Q64119|M	RAT.fasta	16 974,9	100,0 %	3	3	3
sp|P14046|A	RAT.fasta	163 774,4	100,0 %	1	2	2
sp|P55053|F	RAT.fasta	15 059,3	99,5 %	1	1	2
sp|Q6P7S1|A	RAT.fasta	44 444,6	100,0 %	2	2	2
tr|E9PSN2|E9	RAT.fasta	0,0	100,0 %	2	2	2
sp|P19944|R	RAT.fasta	11 498,1	99,5 %	1	1	2
sp|P35053|G	RAT.fasta	61 735,5	100,0 %	8	8	9
tr|Q5M7T5|C	RAT.fasta	52 235,3	100,0 %	2	2	2
sp|P62630|E	RAT.fasta	50 114,2	100,0 %	4	4	5
sp|A2RUV9|A	RAT.fasta	128 063,7	100,0 %	14	15	26
tr|D4A856|D4A856_RAT		0,0	62,0 %	0	0	0
sp|Q62812|M	RAT.fasta	226 343,8	100,0 %	2	2	3
sp|P16975|S	RAT.fasta	34 295,7	100,0 %	15	24	58
tr|Q642B0|Q	RAT.fasta	62 563,0	100,0 %	3	3	3
sp|Q01129|P	RAT.fasta	39 806,8	100,0 %	3	3	4
sp|P08494|M	RAT.fasta	12 037,1	100,0 %	2	2	4
sp|P55051|F	RAT.fasta	14 863,7	99,5 %	1	1	1
sp|P62963|P	RAT.fasta	14 957,3	100,0 %	6	7	8
sp|P63102|1	RAT.fasta	27 771,9	100,0 %	2	2	2
tr|D3Z9F8|D3	RAT.fasta	212 445,0	100,0 %	67	83	134
sp|Q63416|I	RAT.fasta	99 099,5	95,6 %	1	1	1
sp|P05708|H	RAT.fasta	102 411,0	9,7 %	0	0	0
sp|P13697|M	RAT.fasta	64 004,3	9,1 %	0	0	0
tr|F1LUQ1|F1LUQ1_RAT		0,0	52,0 %	0	0	0
tr|D3ZY51|D3	RAT.fasta	67 218,5	99,5 %	1	1	1
sp|Q05982|N	RAT.fasta	17 193,0	100,0 %	1	1	1
tr|D3ZP82|D3	RAT.fasta	83 627,0	100,0 %	4	5	5
sp|P63029|T	RAT.fasta	19 462,9	15,8 %	0	0	0
tr|F1M798|F	RAT.fasta	111 649,0	100,0 %	3	3	3
sp|Q9WUQ1|	RAT.fasta	105 705,8	99,5 %	1	1	1
sp|O88989|M	RAT.fasta	36 483,9	100,0 %	2	2	3
sp|P04764|E	RAT.fasta	47 129,0	100,0 %	8	10	10
tr|F1LP57|F1	RAT.fasta	41 102,6	100,0 %	2	2	2
sp|Q99PW7|I	RAT.fasta	27 107,4	99,9 %	1	1	1
tr|Q9JI04|Q9	RAT.fasta	171 575,7	100,0 %	6	7	9
sp|P23565|A	RAT.fasta	56 116,7	11,7 %	0	0	0
sp|P36953|A	RAT.fasta	69 336,5	59,9 %	0	0	0
sp|P30121|T	RAT.fasta	24 357,0	100,0 %	9	12	22
tr|D4A115|D	RAT.fasta	240 199,7	100,0 %	13	13	14
sp|Q63716|P	RAT.fasta	22 110,2	97,3 %	1	1	1
tr|Q7TQ25|Q7TQ25_RAT		0,0	39,9 %	0	0	0
tr|Q3MIE4|Q3MIE4_RAT		0,0	97,5 %	1	1	2
sp|P04642|L	RAT.fasta	36 450,8	100,0 %	3	3	3
sp|Q9R1E9|C	RAT.fasta	37 755,2	100,0 %	10	11	24

tr|Q5U2V1|Q	RAT.fasta	64 788,7	100,0 %	7	8	11
tr|F1MAL6|F1	RAT.fasta	518 261,9	96,1 %	0	0	0
sp|P10111|PI	RAT.fasta	17 874,8	100,0 %	3	4	4
sp|Q9JI85|NU	RAT.fasta	50 091,2	100,0 %	3	4	5
sp|P11762|LE	RAT.fasta	14 857,2	100,0 %	4	5	6
sp|P05065|AI	RAT.fasta	39 352,6	100,0 %	6	6	9
tr|F1M566|F1	RAT.fasta	230 834,3	100,0 %	12	12	17
sp|P47709|RI	RAT.fasta	75 833,3	71,5 %	0	0	0
sp|Q4FZU2|K	RAT.fasta	59 250,6	100,0 %	4	4	12
sp|P34058|H:	RAT.fasta	83 284,3	99,5 %	1	1	1
sp|P24090|FE	RAT.fasta	37 981,1	90,8 %	0	0	0
sp|P01026|C(	RAT.fasta	186 462,2	100,0 %	3	3	3
sp|P47853|P(	RAT.fasta	41 708,6	100,0 %	16	24	34
sp|P62161|C/	RAT.fasta	16 838,0	100,0 %	2	3	3
tr|F1LP60|F1I	RAT.fasta	67 653,6	100,0 %	11	11	13
sp|P07151|B2	RAT.fasta	13 720,0	100,0 %	2	3	4
sp|Q08420|S(	RAT.fasta	26 619,3	100,0 %	3	5	6
sp|P24268|C/	RAT.fasta	44 682,0	100,0 %	2	2	2
tr|F1LUH9|F1LUH9_RAT		0,0	100,0 %	2	2	3
tr|Q6IFU9|Q6	RAT.fasta	50 777,8	100,0 %	2	3	5
tr|F1MAF7|F1	RAT.fasta	46 837,1	18,8 %	0	0	0
sp|Q5RJP7|P(	RAT.fasta	41 932,9	100,0 %	5	5	5
tr|D3ZQM5|D3ZQM5_RAT		0,0	100,0 %	23	24	31
tr|D4A7Y1|D4	RAT.fasta	224 612,0	100,0 %	21	23	37
sp|P25113|P(	RAT.fasta	28 832,8	99,5 %	1	1	1
sp|P02262|H:	RAT.fasta	14 078,0	100,0 %	2	2	3
sp|P68370|TE	RAT.fasta	50 135,7	99,5 %	1	1	1
tr|D3ZQN7|D	RAT.fasta	202 784,2	93,4 %	1	1	1
sp|Q6IG05|K2	RAT.fasta	59 027,2	7,0 %	0	0	0
tr|D3ZK12|D3ZK12_RAT		0,0	100,0 %	3	3	6
sp|P20909|C(	RAT.fasta	181 029,5	99,5 %	1	1	1
tr|F1LR02|F1	RAT.fasta	134 646,3	6,8 %	0	0	0
sp|Q62611-2|	RAT.fasta	64 406,5	100,0 %	4	4	4
sp|Q5FVH0|C	RAT.fasta	25 334,7	100,0 %	2	2	2
tr|Q6IN22|Q6	RAT.fasta	37 544,1	100,0 %	7	8	12
tr|D3ZQ25|D3	RAT.fasta	78 070,0	100,0 %	3	3	4
tr|D3ZDF6|D3ZDF6_RAT		0,0	99,4 %	1	1	2
sp|P47819|G	RAT.fasta	49 957,9	100,0 %	2	3	5
sp|P51886|LU	RAT.fasta	38 281,3	100,0 %	10	12	21
sp|Q6IFU8|K1	RAT.fasta	48 123,9	100,0 %	5	5	14
sp|P48500|TI	RAT.fasta	26 848,7	100,0 %	4	4	4
sp|Q2Q0I9|FI	RAT.fasta	194 048,8	100,0 %	12	13	15
sp|O88767|P.	RAT.fasta	19 974,3	99,5 %	1	2	3
sp|Q63041|A	RAT.fasta	167 126,0	99,5 %	1	1	1
sp|P97546-3|	RAT.fasta	43 932,2	8,8 %	0	0	0
sp|Q6P0K8|P	RAT.fasta	81 801,9	100,0 %	6	6	6
sp|P0CG51|U	RAT.fasta	14 728,9	99,0 %	1	1	1
tr|D3ZFH5|D3	RAT.fasta	106 528,4	100,0 %	2	2	4
sp|P02454|C(	RAT.fasta	137 953,8	100,0 %	87	133	456
tr|F1LNN9|F1LNN9_RAT		0,0	100,0 %	3	3	5
sp|P31232|T/	RAT.fasta	22 602,7	100,0 %	8	9	12

sp|P70490|M	RAT.fasta	47 413,4	100,0 %	6	6	6
sp|Q00918|L	RAT.fasta	186 590,5	100,0 %	4	4	6
sp|P23785|G	RAT.fasta	63 366,6	100,0 %	7	7	12
tr|D3ZHA0|D	RAT.fasta	290 978,7	95,1 %	0	0	0
sp|Q63772|G	RAT.fasta	74 637,2	100,0 %	3	3	3
sp|P06687|A	RAT.fasta	111 694,4	9,4 %	0	0	0
sp|O35276|N	RAT.fasta	103 899,5	99,5 %	1	1	1
sp|Q6B345|S	RAT.fasta	11 065,2	100,0 %	2	2	2
tr|Q8CHN5|C	RAT.fasta	16 363,9	99,9 %	1	1	4
tr|F1LST1|F1	RAT.fasta	202 546,0	100,0 %	91	132	265
sp|P16636|L	RAT.fasta	46 558,9	100,0 %	2	2	4
sp|P85972|V	RAT.fasta	116 617,3	100,0 %	7	7	11
tr|C0JPT7|C0	RAT.fasta	280 485,3	100,0 %	18	19	26
sp|P07150|A	RAT.fasta	38 831,0	100,0 %	2	2	2
sp|P31000|V	RAT.fasta	53 733,9	100,0 %	17	17	29
sp|P14841|C	RAT.fasta	15 436,8	100,0 %	4	5	10
sp|P51635|A	RAT.fasta	36 506,2	98,7 %	1	1	1
sp|P62804|H	RAT.fasta	11 367,7	100,0 %	2	2	2
sp|P01946|H	RAT.fasta	15 328,7	11,6 %	0	0	0
tr|D3ZAF5|D	RAT.fasta	90 059,4	100,0 %	41	67	107
sp|P02466|C	RAT.fasta	129 564,9	99,6 %	1	1	1
sp|P00762|T	RAT.fasta	25 959,1	100,0 %	3	5	7
sp|Q9WVH8|	RAT.fasta	50 159,8	100,0 %	4	4	6
sp|P30120|T	RAT.fasta	23 794,2	100,0 %	8	9	13
sp|Q9QZQ5|	RAT.fasta	38 508,5	100,0 %	3	3	4
sp|P85973|P	RAT.fasta	32 301,7	47,7 %	0	0	0
sp|P05539|C	RAT.fasta	134 572,0	100,0 %	4	4	10
sp|P30904|M	RAT.fasta	12 477,4	99,5 %	1	1	1
sp|P62738|A	RAT.fasta	42 010,1	100,0 %	8	11	16
tr|F1LTF8|F1	RAT.fasta	195 626,6	93,7 %	0	0	0
tr|D4A645|D4A645_RAT-DE		0,0	100,0 %	2	2	2
sp|P50399|G	RAT.fasta	50 539,1	100,0 %	4	4	5
sp|P07632|S	RAT.fasta	15 910,7	100,0 %	2	2	5
sp|P31044|P	RAT.fasta	20 801,4	99,9 %	1	1	1
sp|P02650|A	RAT.fasta	35 753,4	99,5 %	1	1	1
sp|Q6IFU7|K	RAT.fasta	50 214,0	99,5 %	1	1	1
sp|P20961|P	RAT.fasta	45 010,6	100,0 %	17	23	42
tr|D4ADG9|D	RAT.fasta	66 931,9	100,0 %	3	3	3
sp|Q99MZ8|L	RAT.fasta	29 969,9	89,0 %	0	0	0
sp|P18418|C	RAT.fasta	47 997,0	72,2 %	0	0	0
sp|P07154|C	RAT.fasta	37 660,6	100,0 %	3	4	9
sp|Q5RKI0|W	RAT.fasta	66 181,1	44,6 %	0	0	0
sp|Q6IG01|K	RAT.fasta	57 256,1	6,9 %	0	0	0
tr|Q9ES33|Q	RAT.fasta	30 762,0	100,0 %	7	8	12
tr|D3ZF59|D3ZF59_RAT		0,0	100,0 %	6	6	8
sp|Q6IG03|K	RAT.fasta	60 388,4	100,0 %	2	2	8
sp|P62260|1	RAT.fasta	29 175,0	100,0 %	2	2	2
sp|Q6P6Q2|K	RAT.fasta	61 827,3	100,0 %	13	14	27
tr|Q5XI84|Q5	RAT.fasta	44 849,2	100,0 %	2	2	3
sp|Q9R0D6|T	RAT.fasta	47 421,5	100,0 %	1	1	1
tr|F1LNH3|F1	RAT.fasta	109 660,6	100,0 %	5	6	6

sp|O55004|R	RAT.fasta	16 903,2	100,0 %	2	2	3
tr|Q6IFZ5|Q6	RAT.fasta	61 759,6	99,6 %	1	1	1
tr|D4A185|D4A185_RAT		0,0	100,0 %	2	2	2
tr|D3ZQR7|D	RAT.fasta	87 014,0	100,0 %	13	15	19
sp|Q6QD51|(	RAT.fasta	107 693,2	100,0 %	9	9	10
tr|F1LRL9|F1I	RAT.fasta	269 643,4	97,5 %	1	1	1
sp|Q5U300|U	RAT.fasta	117 788,8	52,6 %	0	0	0
sp|P09495|TI	RAT.fasta	28 510,4	100,0 %	4	4	8
sp|P06238|A:	RAT.fasta	163 785,7	99,5 %	1	1	3
tr|D3ZUL3|D:	RAT.fasta	108 807,3	100,0 %	7	7	10
tr|F1LRZ7|F1I	RAT.fasta	114 411,8	12,7 %	0	0	0
tr|F1LRR0|F1LRR0_RAT,tr|F		0,0	100,0 %	13	16	24
sp|P12346|TI	RAT.fasta	76 395,6	54,9 %	0	0	0
sp|P19804|N	RAT.fasta	17 283,3	100,0 %	1	2	2
sp|O08628|P	RAT.fasta	50 185,1	100,0 %	16	21	35
tr|F1LM84|F1	RAT.fasta	137 037,9	100,0 %	14	16	30
sp|P01830|TI	RAT.fasta	18 172,8	100,0 %	1	1	1
sp|P63018|H:	RAT.fasta	70 872,8	100,0 %	3	3	3
sp|P24368|PI	RAT.fasta	23 802,9	100,0 %	1	1	1
tr|D3ZM43|D3ZM43_RAT		0,0	100,0 %	2	2	2
sp|O35793|G	RAT.fasta	20 680,0	100,0 %	2	2	3
sp|Q7M0E3|I	RAT.fasta	18 534,1	100,0 %	2	2	2
tr|D4A2G6|D	RAT.fasta	129 725,6	100,0 %	30	38	66
sp|P62329|T\	RAT.fasta	5 052,9	100,0 %	2	3	5
tr|Q7TP54|Q	RAT.fasta	144 712,1	100,0 %	2	3	3
sp|Q00715|H	RAT.fasta	13 990,6	99,9 %	1	1	2
sp|Q6AY61|P	RAT.fasta	43 161,1	100,0 %	3	3	3
sp|P05197|EI	RAT.fasta	95 286,0	100,0 %	3	3	3
sp|Q63083|N	RAT.fasta	53 506,9	100,0 %	10	10	11
tr|F1LM30|F1	RAT.fasta	51 745,4	78,9 %	0	0	0
sp|P39069|K/	RAT.fasta	21 584,4	48,9 %	0	0	0
sp|Q5U2Q3|(	RAT.fasta	34 992,9	36,3 %	0	0	0
tr|F1LSW0|F1LSW0_RAT		0,0	100,0 %	15	17	26
sp|Q63434|P	RAT.fasta	17 681,3	100,0 %	3	3	3
tr|D3ZPA9|D3ZPA9_RAT		0,0	100,0 %	8	10	12
sp|P02091|H	RAT.fasta	15 979,4	99,5 %	1	1	1
sp|Q07258|T	RAT.fasta	47 116,4	99,5 %	1	1	2
sp|Q6IFW6|K	RAT.fasta	56 506,5	100,0 %	13	20	49
tr|D3ZWH0|D3ZWH0_RAT-I		0,0	97,8 %	0	0	0
tr|F1LV50|F1	RAT.fasta	34 405,7	99,5 %	1	1	4
sp|O88201|C	RAT.fasta	36 387,5	100,0 %	2	2	2
sp|Q6Q0N0|(	RAT.fasta	106 259,2	100,0 %	2	2	2
tr|D4AC70|D	RAT.fasta	73 452,3	99,9 %	1	1	1
tr|A7M777|A7M777_RAT		0,0	97,5 %	1	1	1
sp|P47942|DI	RAT.fasta	62 277,9	10,4 %	0	0	0
sp|P13941|C(	RAT.fasta	138 936,9	100,0 %	48	63	162
tr|B5DF94|B5DF94_RAT,tr|		0,0	100,0 %	2	2	2
sp|Q6IFV1|K1	RAT.fasta	52 684,9	100,0 %	6	9	16
sp|Q62658|FI	RAT.fasta	11 922,7	99,5 %	1	1	1
tr|F8WG88|F	RAT.fasta	60 600,9	100,0 %	12	17	34
sp|Q9JI03|CC	RAT.fasta	183 990,1	100,0 %	19	29	47

tr|F1LS40|F1I	RAT.fasta	129 838,5	100,0 %	81	120	347
sp|P61983|14	RAT.fasta	28 303,1	99,5 %	1	1	1
sp|P07722|M	RAT.fasta	69 352,9	97,9 %	0	0	0
sp|Q00981|U	RAT.fasta	24 838,2	100,0 %	2	2	2
sp|P05982|N	RAT.fasta	30 947,5	99,9 %	1	1	2
tr|F1M8K0|F:	RAT.fasta	96 707,0	94,4 %	1	1	1
sp|P82995|H:	RAT.fasta	84 818,3	97,5 %	1	1	1
sp|P68255|14	RAT.fasta	27 779,4	99,5 %	1	1	1
sp|P33436|M	RAT.fasta	74 151,7	100,0 %	20	26	35
sp|Q6P9V9|T	RAT.fasta	50 151,7	100,0 %	1	1	1
sp|Q6IFV4|K1	RAT.fasta	47 730,8	100,0 %	2	2	3
sp|P08649|C(	RAT.fasta	192 164,5	99,5 %	1	1	1
sp|Q5XI73|GI	RAT.fasta	23 408,0	100,0 %	3	3	4
sp|P04276|V	RAT.fasta	53 545,6	65,1 %	0	0	0
tr|F1M8G9|F	RAT.fasta	91 166,5	5,0 %	0	0	0
tr|F1LPD0|F1	RAT.fasta	0,0	100,0 %	2	2	3
sp|P18331|IN	RAT.fasta	47 406,9	100,0 %	3	3	5
tr|Q5RJR9|Q!	RAT.fasta	46 563,0	100,0 %	1	1	1
sp|P47875|C:	RAT.fasta	20 613,2	100,0 %	3	3	6
sp|Q6IMF3|K	RAT.fasta	64 831,7	100,0 %	6	10	32
sp|P06761|G	RAT.fasta	72 348,5	100,0 %	4	4	6
sp|Q9R063|P	RAT.fasta	22 178,5	96,3 %	1	1	1
tr|Q9QZK5|Q9QZK5_RAT		0,0	100,0 %	2	2	3
sp|Q9QXQ0|/	RAT.fasta	104 918,2	100,0 %	14	15	26
tr|F1M335|F:	RAT.fasta	164 854,5	100,0 %	2	2	2
tr|E9PSV0|E9PSV0_RAT		0,0	100,0 %	1	1	2
sp|Q07257-2|	RAT.fasta	50 535,3	100,0 %	3	3	7
sp|P05371|CI	RAT.fasta	51 375,6	97,1 %	0	0	0
tr|F1LMV6|F:	RAT.fasta	332 400,6	100,0 %	7	9	10
sp|P58775-2|	RAT.fasta	32 958,6	100,0 %	7	7	7
sp|P85108|TI	RAT.fasta	49 907,1	99,5 %	1	1	1
tr|F1M7P4|F:	RAT.fasta	53 983,3	22,0 %	0	0	0
tr|Q3MID6|Q	RAT.fasta	37 148,8	100,0 %	5	7	16
sp|P10960|S/	RAT.fasta	61 122,8	100,0 %	6	7	9
tr|B1WC91|B	RAT.fasta	35 043,1	100,0 %	1	1	1
tr|F1LS57|F1I	RAT.fasta	125 961,6	100,0 %	7	7	11
sp|Q9EPB1|D	RAT.fasta	55 115,3	100,0 %	4	4	4
tr|F1LPM2|F1LPM2_RAT		0,0	100,0 %	2	2	3
sp|Q9Z1P2|A	RAT.fasta	102 963,9	100,0 %	6	6	9
sp|P12839|N	RAT.fasta	95 790,7	96,9 %	1	1	1
sp|P42123|LI	RAT.fasta	36 612,5	99,5 %	1	1	1
sp|Q6AY84|S	RAT.fasta	46 396,0	11,0 %	0	0	0
tr|F1M9B2|F:	RAT.fasta	28 948,7	100,0 %	10	14	25
sp|Q07936|A	RAT.fasta	38 680,2	100,0 %	3	3	3
sp|P02770|AI	RAT.fasta	68 731,2	100,0 %	2	2	5
sp|P16617|P(	RAT.fasta	44 539,1	99,0 %	1	1	1
sp|Q63610|T	RAT.fasta	29 007,2	100,0 %	2	2	2
sp|P45592|C(	RAT.fasta	18 533,2	100,0 %	3	3	3
sp|P08721|O.	RAT.fasta	34 962,7	100,0 %	7	8	14
tr|D4A0Y1|D4A0Y1_RAT		0,0	100,0 %	4	4	4
tr|Q6AYQ9|Q	RAT.fasta	23 009,6	100,0 %	5	6	7

tr|F1LQ00|F1	RAT.fasta	142 475,8	100,0 %	39	49	121
sp|P60711|A(	RAT.fasta	41 737,8	100,0 %	13	22	38
sp|Q9R0J8|L(	RAT.fasta	49 465,8	100,0 %	2	2	2
tr|F1MA59|F1	RAT.fasta	160 614,4	100,0 %	5	7	8
tr|F1MAA7|F	RAT.fasta	177 385,1	100,0 %	2	2	2
sp|Q6IG02|K2	RAT.fasta	69 127,7	100,0 %	4	7	16
sp|P07323|EI	RAT.fasta	47 141,5	100,0 %	2	2	2
tr|D3ZRK9|D3	RAT.fasta	0,0	100,0 %	2	2	4
sp|P04692-3|	RAT.fasta	32 682,0	100,0 %	2	2	2
sp|Q9ERB4-2	RAT.fasta	300 004,9	96,3 %	1	1	1
tr|B2RYM3|B	RAT.fasta	100 589,7	100,0 %	3	3	3
tr|F1LYE8|F1LYE8_RAT		0,0	100,0 %	2	2	2
sp|P24090|FE	RAT.fasta	37 981,1	100,0 %	15	23	35
sp|Q63560|M	RAT.fasta	100 484,2	100,0 %	5	5	12
sp|P10760|SA	RAT.fasta	47 538,9	100,0 %	4	4	4
sp|Q5U2Q3|(	RAT.fasta	34 992,9	100,0 %	3	3	3
sp|P07722|M	RAT.fasta	69 352,9	100,0 %	7	10	13
tr|B2RZ27|B2	RAT.fasta	10 476,8	100,0 %	2	3	3
tr|F1LM42|F1	RAT.fasta	434 346,5	100,0 %	2	2	2
sp|P13221|AA	RAT.fasta	46 429,5	100,0 %	15	20	30
sp|Q5FVJ0-2|	RAT.fasta	52 909,0	100,0 %	2	2	2
sp|P35704|PI	RAT.fasta	21 784,1	100,0 %	10	11	20
sp|P42123|LD	RAT.fasta	36 612,5	100,0 %	20	27	43
tr|D4A856|D4A856_RAT		0,0	100,0 %	2	2	2
sp|P18418|CA	RAT.fasta	47 997,0	100,0 %	5	5	6
tr|D4A111|D4	RAT.fasta	306 150,3	100,0 %	3	3	4
sp|Q9JLJ3|AL	RAT.fasta	53 653,2	100,0 %	2	2	3
tr|F1LPD0|F1	RAT.fasta	0,0	98,7 %	1	1	1
sp|Q920J4|TX	RAT.fasta	32 249,5	100,0 %	5	5	6
sp|Q5RKI0|W	RAT.fasta	66 181,1	100,0 %	5	5	8
sp|P47728|CA	RAT.fasta	31 406,7	100,0 %	3	3	3
tr|D3ZQP6|D3	RAT.fasta	75 143,5	100,0 %	2	3	7
sp|P25113|P(	RAT.fasta	28 832,8	100,0 %	12	16	25
sp|P04797|G3	RAT.fasta	35 828,1	100,0 %	11	14	25
sp|P20760|IG	RAT.fasta	35 184,7	100,0 %	17	25	73
sp|Q6IG05|K2	RAT.fasta	59 027,2	28,2 %	0	0	0
tr|F1LQI1|F1I	RAT.fasta	34 157,9	100,0 %	2	2	3
tr|Q5M7T5|C	RAT.fasta	52 235,3	100,0 %	3	3	5
sp|Q6PTT0|A	RAT.fasta	45 822,2	100,0 %	2	2	2
sp|P47819|G	RAT.fasta	49 957,9	100,0 %	26	32	64
sp|Q64240|A	RAT.fasta	38 851,4	100,0 %	3	3	6
sp|Q6IG01|K2	RAT.fasta	57 256,1	5,4 %	0	0	0
tr|D3ZXM9|D	RAT.fasta	131 885,3	100,0 %	3	3	3
tr|F1M7P4|F1	RAT.fasta	53 983,3	100,0 %	2	3	4
sp|Q63041|A	RAT.fasta	167 126,0	100,0 %	32	39	71
sp|Q64361|L1	RAT.fasta	25 579,1	100,0 %	5	6	11
sp|B0BND0|E	RAT.fasta	50 702,6	100,0 %	9	10	10
sp|P04905|G3	RAT.fasta	25 915,9	100,0 %	3	3	3
sp|Q63945|SI	RAT.fasta	33 406,4	100,0 %	2	2	2
sp|P12839|N	RAT.fasta	95 790,7	100,0 %	42	54	86
tr|F1MAL6|F1	RAT.fasta	518 261,9	100,0 %	21	22	31

sp|Q62812|M	RAT.fasta	226 343,8	100,0 %	2	2	3
sp|P06761|G	RAT.fasta	72 348,5	100,0 %	6	7	13
sp|Q6P6V0|G	RAT.fasta	62 829,3	100,0 %	14	19	27
tr|Q9JKB7|Q9	RAT.fasta	50 901,6	100,0 %	6	7	9
tr|F1M8G9|F	RAT.fasta	91 166,5	100,0 %	2	2	2
sp|Q68FP1-2|	RAT.fasta	86 067,9	100,0 %	6	6	6
sp|Q5XI32|CA	RAT.fasta	30 629,7	100,0 %	2	2	3
tr|F1MAG6|F1MAG6_RAT		0,0	100,0 %	6	6	9
sp|P19527|N	RAT.fasta	61 336,5	100,0 %	36	43	86
sp|P02680-2|	RAT.fasta	50 634,3	100,0 %	10	14	24
sp|P35213|14	RAT.fasta	28 055,2	100,0 %	5	6	7
sp|P08009|G	RAT.fasta	25 682,7	100,0 %	7	7	9
sp|Q62930|C	RAT.fasta	63 776,5	100,0 %	5	6	7
sp|P07335|K	RAT.fasta	42 726,1	100,0 %	18	24	39
sp|Q6P9V9|T	RAT.fasta	50 151,7	100,0 %	24	29	39
sp|P07323|EI	RAT.fasta	47 141,5	100,0 %	23	32	47
sp|Q63768-2|	RAT.fasta	33 844,8	100,0 %	2	2	2
sp|P05708|H	RAT.fasta	102 411,0	100,0 %	5	5	6
tr|B0K010|B0	RAT.fasta	14 091,9	100,0 %	2	2	2
sp|P07936|N	RAT.fasta	23 603,2	100,0 %	8	9	15
sp|Q64119|M	RAT.fasta	16 974,9	100,0 %	1	1	1
sp|P04276|V	RAT.fasta	53 545,6	100,0 %	13	16	30
sp|P01946|H	RAT.fasta	15 328,7	100,0 %	24	34	95
sp|P39069|KA	RAT.fasta	21 584,4	100,0 %	4	4	5
tr|D3ZFC6|D3	RAT.fasta	103 757,3	100,0 %	28	33	50
sp|P23593|AI	RAT.fasta	21 635,8	100,0 %	4	5	7
sp|Q64537|C	RAT.fasta	28 571,1	100,0 %	2	2	3
sp|P14046|A1	RAT.fasta	163 774,4	100,0 %	25	35	49
sp|P14668|AI	RAT.fasta	35 746,5	100,0 %	3	3	4
sp|P14480|FI	RAT.fasta	54 235,8	100,0 %	22	25	36
sp|O88767|P	RAT.fasta	19 974,3	100,0 %	9	10	15
sp|P09117|AI	RAT.fasta	39 284,3	100,0 %	15	19	25
sp|P12346|TI	RAT.fasta	76 395,6	100,0 %	62	92	168
sp|Q9WUW3	RAT.fasta	67 296,6	100,0 %	4	5	7
sp|P50398|G	RAT.fasta	50 538,2	100,0 %	7	8	13
sp|P60881|SI	RAT.fasta	23 315,4	100,0 %	2	2	2
tr|E9PSN2|E9	RAT.fasta	0,0	35,7 %	0	0	0
sp|P97685-3|	RAT.fasta	132 181,4	100,0 %	6	6	7
tr|F1LR02|F1	RAT.fasta	134 646,3	100,0 %	2	2	3
sp|P04904|G	RAT.fasta	25 320,4	100,0 %	3	4	6
sp|P11030|A	RAT.fasta	10 027,9	100,0 %	7	12	14
sp|P63329-2|	RAT.fasta	58 645,7	100,0 %	2	2	2
sp|P06399|FI	RAT.fasta	86 687,8	100,0 %	13	16	23
sp|P35745|A	RAT.fasta	10 863,2	100,0 %	4	4	4
tr|F1M9K9|F1	RAT.fasta	120 417,4	100,0 %	3	3	5
sp|P23785|G	RAT.fasta	63 366,6	100,0 %	4	4	7
sp|P47875|CS	RAT.fasta	20 613,2	100,0 %	3	3	4
tr|F1LPS8|F1I	RAT.fasta	33 737,1	100,0 %	2	2	3
sp|Q9JHU0|D	RAT.fasta	61 539,8	100,0 %	3	3	3
sp|P50399|G	RAT.fasta	50 539,1	100,0 %	8	10	12
sp|Q4FZU2|K	RAT.fasta	59 250,6	98,7 %	1	1	2

sp|P03994|H	RAT.fasta	40 262,4	100,0 %	4	5	6
sp|P13233|CI	RAT.fasta	47 270,0	100,0 %	12	13	15
sp|P31232|T/	RAT.fasta	22 602,7	98,7 %	1	1	2
sp|P48500|TI	RAT.fasta	26 848,7	100,0 %	19	29	50
tr|F1LM30|F1	RAT.fasta	51 745,4	100,0 %	5	7	15
tr|D3ZFH5|D:	RAT.fasta	106 528,4	94,0 %	0	0	0
tr|D3ZUK7|D3ZUK7_RAT		0,0	100,0 %	2	2	2
sp|P69897|TI	RAT.fasta	49 670,6	100,0 %	5	5	6
tr|F1LRX5|F1LRX5_RAT		0,0	100,0 %	15	17	21
tr|Q7TQ25|Q7TQ25_RAT		0,0	100,0 %	5	6	9
sp|P34058|H:	RAT.fasta	83 284,3	100,0 %	21	23	40
sp|P20759|IG	RAT.fasta	35 944,3	100,0 %	4	7	11
tr|F8WFW0|F8WFW0_RAT		0,0	100,0 %	2	2	2
sp|Q9EQS0|T	RAT.fasta	37 461,8	100,0 %	6	6	7
sp|P10860|DI	RAT.fasta	61 417,4	100,0 %	2	2	2
sp|P85845|FS	RAT.fasta	54 491,2	100,0 %	3	3	3
sp|P05197|EI	RAT.fasta	95 286,0	100,0 %	16	17	21
sp|P62630|EI	RAT.fasta	50 114,2	100,0 %	5	5	6
sp|P52303-2|	RAT.fasta	104 591,4	100,0 %	3	3	4
sp|P02454|C(	RAT.fasta	137 953,8	76,4 %	0	0	0
sp|P60203|M	RAT.fasta	30 077,7	100,0 %	6	6	10
tr|F1LQ63|F1	RAT.fasta	139 429,9	100,0 %	9	9	10
sp|P47709|RI	RAT.fasta	75 833,3	100,0 %	2	2	4
tr|D3ZVB7|D:	RAT.fasta	34 071,5	100,0 %	1	2	2
sp|Q5I0D1|G	RAT.fasta	33 268,2	100,0 %	2	2	2
sp|Q9QUL6|N	RAT.fasta	82 655,5	100,0 %	3	3	4
sp|P55051|F/	RAT.fasta	14 863,7	100,0 %	11	15	20
tr|Q5PQU1|Q5PQU1_RAT		0,0	100,0 %	9	11	24
sp|Q6IFW6|K	RAT.fasta	56 506,5	100,0 %	7	7	10
sp|P05544|SI	RAT.fasta	46 279,0	100,0 %	4	4	5
sp|P31596-2|	RAT.fasta	62 108,3	100,0 %	3	4	7
sp|P24268|C/	RAT.fasta	44 682,0	99,8 %	1	1	1
sp|P04906|G:	RAT.fasta	23 439,8	100,0 %	6	6	7
tr|D4A7Y1|D4	RAT.fasta	224 612,0	100,0 %	5	6	6
sp|P16086|SPTA2_RAT		0,0	100,0 %	40	50	61
sp|Q00981|U	RAT.fasta	24 838,2	100,0 %	13	19	28
sp|P31000|VI	RAT.fasta	53 733,9	100,0 %	21	23	37
tr|F1M6Q3|F	RAT.fasta	166 252,0	61,9 %	0	0	0
sp|P31722|C:	RAT.fasta	25 685,9	100,0 %	3	3	6
sp|P00697|LY	RAT.fasta	16 729,2	100,0 %	4	6	9
sp|P63018|H:	RAT.fasta	70 872,8	100,0 %	20	27	43
sp|P02688-2|	RAT.fasta	18 488,3	100,0 %	14	18	77
tr|D3ZJ08|D3	RAT.fasta	15 388,7	100,0 %	1	1	1
sp|P08649|C(	RAT.fasta	192 164,5	100,0 %	2	2	2
sp|P85515|A(	RAT.fasta	42 615,2	100,0 %	2	2	2
sp|P60711|A(	RAT.fasta	41 737,8	100,0 %	24	29	46
sp|P62804|H4	RAT.fasta	11 367,7	100,0 %	3	4	6
sp|P11762|LE	RAT.fasta	14 857,2	100,0 %	3	3	6
sp|P61265|ST	RAT.fasta	33 245,1	100,0 %	2	2	3
tr|D3Z8P5|D:	RAT.fasta	35 269,8	100,0 %	3	3	5
sp|P85973|PI	RAT.fasta	32 301,7	100,0 %	3	3	5

sp|Q00715|H	RAT.fasta	13 990,6	100,0 %	4	4	5
tr|C0JPT7|C0	RAT.fasta	280 485,3	100,0 %	6	6	7
sp|Q6IFV1|K1	RAT.fasta	52 684,9	76,6 %	0	0	0
tr|E9PSV0|E9PSV0_RAT		0,0	100,0 %	5	5	9
sp|P40241|CI	RAT.fasta	25 215,7	100,0 %	2	3	4
sp|Q07936|A	RAT.fasta	38 680,2	100,0 %	3	3	4
tr|F1M983|F1	RAT.fasta	140 007,0	100,0 %	11	16	22
sp|P07150|AI	RAT.fasta	38 831,0	99,8 %	1	1	1
tr|F1MAA7|F	RAT.fasta	177 385,1	98,7 %	1	1	2
sp|P62738|A(	RAT.fasta	42 010,1	100,0 %	2	2	3
tr|D3ZS68|D3ZS68_RAT		0,0	100,0 %	4	4	5
sp|P37805|T/	RAT.fasta	22 500,9	100,0 %	3	3	7
sp|Q5XI73|GI	RAT.fasta	23 408,0	100,0 %	4	4	5
sp|P45592|C(	RAT.fasta	18 533,2	100,0 %	12	14	19
sp|Q62718|N	RAT.fasta	37 997,6	100,0 %	3	3	5
tr|Q9JI04|Q9	RAT.fasta	171 575,7	19,4 %	0	0	0
sp|P36953|AI	RAT.fasta	69 336,5	100,0 %	8	8	9
sp|P11980|KI	RAT.fasta	57 818,6	100,0 %	35	45	62
sp|P04639|AI	RAT.fasta	30 062,4	100,0 %	3	3	3
sp|P62260|14	RAT.fasta	29 175,0	100,0 %	8	11	13
sp|P11348|DI	RAT.fasta	25 552,3	100,0 %	4	6	9
tr|F1LP60|F1I	RAT.fasta	67 653,6	100,0 %	4	4	4
tr|F1LNH3|F1	RAT.fasta	109 660,6	13,2 %	0	0	0
sp|P19332-2|	RAT.fasta	71 773,6	100,0 %	2	2	3
tr|E9PSV5|E9	RAT.fasta	40 490,9	100,0 %	2	2	2
sp|Q6QD51|(	RAT.fasta	107 693,2	75,3 %	0	0	0
sp|P02770|AI	RAT.fasta	68 731,2	100,0 %	89	137	385
sp|Q5FVI4|CE	RAT.fasta	15 043,2	100,0 %	5	6	9
sp|P14141|C/	RAT.fasta	29 431,8	100,0 %	9	12	26
tr|B2RZA9|B2	RAT.fasta	17 862,3	100,0 %	3	3	3
tr|D4A115|D4	RAT.fasta	240 199,7	100,0 %	23	28	30
sp|Q68FS4-2|	RAT.fasta	56 151,2	100,0 %	6	6	7
tr|D4ABR6|D4	RAT.fasta	0,0	100,0 %	3	3	3
sp|Q9QZ76|M	RAT.fasta	17 157,5	100,0 %	5	7	7
sp|P13697|M	RAT.fasta	64 004,3	100,0 %	2	2	2
sp|Q63716|P	RAT.fasta	22 110,2	100,0 %	13	16	26
sp|Q6P7Q4|L	RAT.fasta	20 820,7	100,0 %	3	3	4
sp|P55068|P(	RAT.fasta	96 057,1	100,0 %	8	8	8
sp|P11598|PI	RAT.fasta	56 625,5	100,0 %	7	7	10
sp|P11980-2|	RAT.fasta	57 781,6	100,0 %	2	2	2
sp|P09006|SI	RAT.fasta	46 654,0	100,0 %	2	2	2
sp|P47942|DI	RAT.fasta	62 277,9	100,0 %	27	32	53
sp|P0CG51|U	RAT.fasta	14 728,9	100,0 %	3	3	7
sp|P15999|A1	RAT.fasta	59 755,2	100,0 %	4	4	5
tr|D3ZF59|D3ZF59_RAT		0,0	39,7 %	0	0	0
tr|D3ZGK7|D3	RAT.fasta	60 310,5	100,0 %	7	9	10
tr|Q66HI5|Q6	RAT.fasta	21 100,1	100,0 %	6	7	10
sp|P50137|TI	RAT.fasta	67 644,7	100,0 %	12	12	13
sp|P46462|TE	RAT.fasta	89 351,8	100,0 %	14	16	20
tr|D4A0Y1|D4A0Y1_RAT		0,0	100,0 %	3	4	4
sp|Q62952|D	RAT.fasta	61 967,8	100,0 %	3	3	5

tr|Q6IN22|Q6	RAT.fasta	37 544,1	100,0 %	11	13	18
sp|P10111|PI	RAT.fasta	17 874,8	100,0 %	16	18	30
sp|P29315|RI	RAT.fasta	49 975,6	100,0 %	6	6	6
sp|P11232|TH	RAT.fasta	11 673,3	100,0 %	2	2	4
sp|O08838|A	RAT.fasta	74 878,1	100,0 %	2	2	3
tr|D3ZEI4|D3	RAT.fasta	46 530,2	100,0 %	2	2	2
sp|Q6P6Q2|K	RAT.fasta	61 827,3	98,7 %	1	1	1
tr|D3ZQ25|D3	RAT.fasta	78 070,0	89,2 %	1	1	1
tr|F1LNN9|F1LNN9_RAT		0,0	100,0 %	7	10	15
sp|P06686|AT	RAT.fasta	112 220,7	100,0 %	4	4	6
sp|Q9ESM2|H	RAT.fasta	38 047,2	100,0 %	7	8	19
sp|P11517|H	RAT.fasta	15 982,3	100,0 %	9	12	28
sp|P00564|KC	RAT.fasta	43 045,6	100,0 %	3	3	3
sp|P16290|PC	RAT.fasta	28 755,7	100,0 %	4	5	10
sp|P04642|LD	RAT.fasta	36 450,8	100,0 %	10	11	14
tr|E9PSN4|E9	RAT.fasta	203 528,4	11,5 %	0	0	0
sp|Q9QX79|F	RAT.fasta	41 532,1	100,0 %	7	9	11
sp|O88989|M	RAT.fasta	36 483,9	100,0 %	13	13	21
tr|F8WGA3|F	RAT.fasta	42 336,0	100,0 %	2	2	2
sp|P16617|PC	RAT.fasta	44 539,1	100,0 %	19	23	26
sp|Q9R0J8|LC	RAT.fasta	49 465,8	75,3 %	0	0	0
tr|D4ADG9|D	RAT.fasta	66 931,9	34,1 %	0	0	0
sp|Q6AY61|P	RAT.fasta	43 161,1	6,5 %	0	0	0
sp|Q6IFU8|K1	RAT.fasta	48 123,9	98,7 %	1	1	2
sp|Q6IMF3|K	RAT.fasta	64 831,7	100,0 %	2	3	4
tr|F1M9V7|F1	RAT.fasta	103 347,0	100,0 %	6	6	8
sp|Q5GFD9|II	RAT.fasta	35 995,6	100,0 %	2	2	2
sp|Q63610|T	RAT.fasta	29 007,2	100,0 %	5	6	8
tr|F1LUV9|F1	RAT.fasta	92 311,9	100,0 %	19	23	37
sp|Q5I0D7|PI	RAT.fasta	54 750,3	100,0 %	4	4	4
sp|Q6P9T8|TBB2C_RAT		0,0	100,0 %	3	4	7
sp|P51886|LU	RAT.fasta	38 281,3	100,0 %	8	11	15
sp|P10719|AT	RAT.fasta	56 354,3	100,0 %	3	3	4
sp|P30009|M	RAT.fasta	29 794,2	100,0 %	9	9	14
tr|D3ZWH0|D3ZWH0_RAT-I		0,0	95,2 %	0	0	0
sp|P07154|CA	RAT.fasta	37 660,6	100,0 %	2	2	3
sp|P09812|PY	RAT.fasta	97 276,7	100,0 %	3	3	3
sp|P85972|VI	RAT.fasta	116 617,3	100,0 %	1	1	1
tr|Q5XI38|Q5	RAT.fasta	70 124,7	100,0 %	7	9	12
sp|Q9WUC4|	RAT.fasta	7 292,5	100,0 %	2	2	2
sp|P58775-2|	RAT.fasta	32 958,6	100,0 %	3	4	6
sp|P38652|PC	RAT.fasta	61 405,2	100,0 %	6	7	10
sp|P07340|AT	RAT.fasta	35 203,6	100,0 %	4	4	5
sp|P04636|M	RAT.fasta	35 684,2	100,0 %	11	11	14
sp|Q6IG02|K2	RAT.fasta	69 127,7	100,0 %	3	3	3
sp|P04631|S1	RAT.fasta	10 743,9	100,0 %	3	7	10
sp|Q6Q0N1|C	RAT.fasta	52 694,2	100,0 %	2	2	3
sp|P09495|TP	RAT.fasta	28 510,4	99,7 %	1	1	1
sp|Q3B8Q0|N	RAT.fasta	36 988,4	100,0 %	2	2	3
sp|Q9EPC6|P	RAT.fasta	15 002,1	100,0 %	5	5	8
sp|Q03626|M	RAT.fasta	165 327,3	100,0 %	9	13	22

sp|O35244|P	RAT.fasta	24 819,9	100,0 %	5	7	8
tr|A7M778|A	RAT.fasta	53 484,3	100,0 %	1	1	1
sp|P09606|G	RAT.fasta	42 268,3	100,0 %	4	4	5
sp|P63102|1	RAT.fasta	27 771,9	100,0 %	15	20	28
tr|F1LM84|F1	RAT.fasta	137 037,9	100,0 %	1	1	2
sp|Q9QXQ0|	RAT.fasta	104 918,2	100,0 %	8	8	9
sp|P06238|A	RAT.fasta	163 785,7	100,0 %	2	2	3
sp|Q6P6R2|D	RAT.fasta	54 038,2	100,0 %	3	3	3
tr|Q5I0M1|Q	RAT.fasta	38 457,3	100,0 %	9	12	16
sp|P01026|C	RAT.fasta	186 462,2	100,0 %	20	23	33
tr|D4A133|D	RAT.fasta	68 266,4	100,0 %	2	2	2
sp|P20761|IG	RAT.fasta	36 495,8	100,0 %	5	7	10
sp|Q01177|P	RAT.fasta	90 535,1	100,0 %	12	13	23
sp|Q9Z1P2|A	RAT.fasta	102 963,9	98,7 %	1	1	1
tr|F1LST1|F1	RAT.fasta	202 546,0	100,0 %	27	32	53
tr|F1LQ00|F1	RAT.fasta	142 475,8	14,4 %	0	0	0
tr|D3ZHC4|D	RAT.fasta	22 950,8	100,0 %	3	3	3
tr|D3ZQM5|D3ZQM5_RAT		0,0	76,2 %	0	0	0
tr|Q7TP54|Q	RAT.fasta	144 712,1	100,0 %	9	13	16
tr|B2GV03|B2GV03_RAT		0,0	100,0 %	8	9	9
sp|P04785|P	RAT.fasta	56 953,4	100,0 %	3	3	5
sp|B0BNN3|C	RAT.fasta	28 299,9	100,0 %	12	15	30
tr|D3ZZX3|D3ZZX3_RAT		0,0	100,0 %	28	34	43
sp|P34926|M	RAT.fasta	299 530,8	100,0 %	7	7	10
sp|Q5XFX0|T	RAT.fasta	22 393,8	100,0 %	4	4	6
sp|Q63198|C	RAT.fasta	113 497,1	100,0 %	7	7	13
sp|P02767|T	RAT.fasta	15 719,9	100,0 %	3	3	4
tr|F1LMV6|F	RAT.fasta	332 400,6	99,3 %	0	0	0
sp|Q9ER34|A	RAT.fasta	85 436,1	100,0 %	5	5	7
sp|P06687|A	RAT.fasta	111 694,4	100,0 %	18	18	27
sp|Q63416|IT	RAT.fasta	99 099,5	100,0 %	9	10	13
sp|P63041|C	RAT.fasta	15 122,7	100,0 %	2	2	2
tr|D4A6X4|D	RAT.fasta	11 311,9	100,0 %	2	2	2
sp|P27274|C	RAT.fasta	13 790,5	100,0 %	4	4	9
tr|Q3MHS9|C	RAT.fasta	58 018,4	100,0 %	2	2	2
tr|Q8CHN5|C	RAT.fasta	16 363,9	100,0 %	1	1	2
tr|F1M7I8|F1	RAT.fasta	13 577,6	100,0 %	2	2	2
sp|P20961|P	RAT.fasta	45 010,6	100,0 %	4	5	6
sp|P04692-3|	RAT.fasta	32 682,0	98,7 %	1	1	1
sp|P02091|H	RAT.fasta	15 979,4	100,0 %	39	61	146
sp|Q6B345|S	RAT.fasta	11 065,2	100,0 %	2	2	2
sp|P55067|N	RAT.fasta	0,0	100,0 %	4	4	4
tr|D3ZRK9|D	RAT.fasta	0,0	100,0 %	4	4	4
tr|F1LS57|F1	RAT.fasta	125 961,6	34,5 %	0	0	0
sp|P08413|K	RAT.fasta	60 402,3	100,0 %	2	2	2
sp|P08699|LE	RAT.fasta	27 202,1	100,0 %	3	4	5
sp|Q1WIM1|	RAT.fasta	42 780,2	100,0 %	6	7	15
sp|Q9ERB4-2	RAT.fasta	300 004,9	100,0 %	14	18	33
sp|P02401|R	RAT.fasta	11 692,3	100,0 %	5	5	7
sp|P48199|C	RAT.fasta	25 468,3	100,0 %	3	3	4
sp|P61983|1	RAT.fasta	28 303,1	100,0 %	11	13	18

tr|D3ZTB5|D3	RAT.fasta	11 198,3	100,0 %	2	2	3
sp|Q7M0E3|I	RAT.fasta	18 534,1	100,0 %	2	2	3
sp|P20059|H	RAT.fasta	51 351,5	100,0 %	19	25	39
sp|P28073|PS	RAT.fasta	25 289,6	100,0 %	2	2	2
sp|Q5PPN5|T	RAT.fasta	18 980,2	100,0 %	4	4	5
sp|P07943|AI	RAT.fasta	35 797,7	100,0 %	5	5	7
sp|P68255|14	RAT.fasta	27 779,4	100,0 %	6	6	8
tr|F1LRL9|F1I	RAT.fasta	269 643,4	100,0 %	5	5	6
sp|P07483|FA	RAT.fasta	14 774,8	100,0 %	2	2	3
sp|P20767|LA	RAT.fasta	11 317,4	100,0 %	5	6	12
sp|Q6AYC4|C	RAT.fasta	38 799,7	99,9 %	1	1	4
sp|P30904|M	RAT.fasta	12 477,4	100,0 %	2	2	6
sp|P09951-2|	RAT.fasta	69 909,4	100,0 %	3	3	4
sp|P23565|AI	RAT.fasta	56 116,7	100,0 %	12	12	20
sp|P50115|S1	RAT.fasta	10 238,4	100,0 %	3	3	4
sp|O35077|G	RAT.fasta	37 452,4	100,0 %	4	4	5
sp|P15429|EI	RAT.fasta	47 015,0	100,0 %	6	6	7
sp|P63029|T(	RAT.fasta	19 462,9	100,0 %	2	3	4
sp|Q63544|S	RAT.fasta	12 918,2	100,0 %	6	9	16
sp|Q9JK11|R	RAT.fasta	126 391,0	100,0 %	6	6	8
tr|D3ZQN7|D	RAT.fasta	202 784,2	7,2 %	0	0	0
sp|P85971|6I	RAT.fasta	27 235,0	100,0 %	2	3	4
sp|P62963|PI	RAT.fasta	14 957,3	100,0 %	7	9	10
tr|Q63910|Q(	RAT.fasta	15 525,0	100,0 %	3	3	4
sp|P07895|S(	RAT.fasta	24 674,3	100,0 %	3	3	3
sp|P48679|LI	RAT.fasta	74 325,0	100,0 %	6	7	7
sp|P97697|IN	RAT.fasta	30 495,6	100,0 %	2	2	3
sp|Q08163|C	RAT.fasta	51 588,9	100,0 %	5	6	8
sp|P68511|14	RAT.fasta	28 212,6	100,0 %	3	3	5
sp|P36972|AI	RAT.fasta	19 546,6	35,9 %	0	0	0
tr|B5DF65|B5	RAT.fasta	22 093,7	100,0 %	2	2	2
tr|D3ZWH5|D	RAT.fasta	28 014,0	100,0 %	2	2	4
sp|P51635|AI	RAT.fasta	36 506,2	100,0 %	3	3	5
sp|Q9Z0W7|(	RAT.fasta	28 634,4	100,0 %	2	2	3
sp|Q5U300|U	RAT.fasta	117 788,8	100,0 %	3	3	3
sp|Q2PQA9|K	RAT.fasta	109 531,6	100,0 %	2	2	2
sp|P17475|A1	RAT.fasta	46 137,6	100,0 %	15	19	28
sp|P30120|TI	RAT.fasta	23 794,2	19,5 %	0	0	0
tr|F1M853|F1	RAT.fasta	170 987,4	100,0 %	2	2	2
tr|Q9QX80|Q	RAT.fasta	30 853,6	100,0 %	2	2	2
sp|P05065|AI	RAT.fasta	39 352,6	100,0 %	26	32	52
sp|Q64559-1|	RAT.fasta	37 560,3	100,0 %	3	3	3
sp|Q05982|N	RAT.fasta	17 193,0	100,0 %	10	12	22
sp|Q6AY84|S	RAT.fasta	46 396,0	100,0 %	5	5	7
sp|P85108|TI	RAT.fasta	49 907,1	100,0 %	23	33	52
sp|P52759|U	RAT.fasta	14 303,8	100,0 %	3	3	5
sp|Q5I0D5|LI	RAT.fasta	29 190,3	100,0 %	2	2	2
tr|D4A5L9|D4	RAT.fasta	11 636,0	100,0 %	4	5	8
sp|P22057|P	RAT.fasta	21 301,7	100,0 %	4	4	5
sp|P14841|C	RAT.fasta	15 436,8	100,0 %	9	11	19
sp|P11442|CI	RAT.fasta	0,0	100,0 %	7	7	9

sp|P08932|KI	RAT.fasta	47 704,1	100,0 %	28	41	97
sp|Q6MGD0|	RAT.fasta	18 658,7	100,0 %	2	3	3
tr|Q62669|Q	RAT.fasta	16 022,6	100,0 %	7	8	10
tr|F1M566|F:	RAT.fasta	230 834,3	100,0 %	13	13	20
tr|Q6P9V6|Q	RAT.fasta	26 411,3	100,0 %	2	3	4
sp|Q64303|P.	RAT.fasta	57 962,1	100,0 %	2	2	3
sp|P50229|C(	RAT.fasta	10 335,1	100,0 %	3	3	3
tr|D3ZVQ0|D	RAT.fasta	95 780,8	100,0 %	2	2	2
sp|P00762|TI	RAT.fasta	25 959,1	98,7 %	1	2	6
sp|Q9EPH1|A	RAT.fasta	56 479,3	100,0 %	10	11	15
sp|P02650|AI	RAT.fasta	35 753,4	100,0 %	5	5	14
sp|P06866|H	RAT.fasta	38 563,0	100,0 %	18	22	42
sp|Q78P75|D	RAT.fasta	10 350,1	100,0 %	3	3	5
sp|Q4QRB4|T	RAT.fasta	50 418,7	100,0 %	9	10	12
tr|F1LRZ7|F1I	RAT.fasta	114 411,8	100,0 %	25	31	37
sp|P19944|RI	RAT.fasta	11 498,1	100,0 %	2	2	2
sp|Q5QD51-2	RAT.fasta	173 057,0	100,0 %	4	4	5
sp|P02262|H:	RAT.fasta	14 078,0	100,0 %	7	9	13
sp|Q5XIF6|TE	RAT.fasta	49 924,6	100,0 %	3	4	5
tr|F1LPR6|F1	RAT.fasta	41 307,3	100,0 %	3	3	5
sp|O35987|N	RAT.fasta	40 680,2	100,0 %	3	3	3
sp|Q99PS8|H	RAT.fasta	59 047,3	100,0 %	7	7	10
sp|Q63691|C	RAT.fasta	40 055,2	100,0 %	4	5	7
tr|D3ZHA0|D:	RAT.fasta	290 978,7	100,0 %	15	15	19
tr|D3ZFY8|D3	RAT.fasta	16 354,8	100,0 %	4	4	5
tr|D3ZD09|D:	RAT.fasta	10 071,5	100,0 %	2	2	2
sp|P80254|D(	RAT.fasta	13 133,9	100,0 %	7	9	12
sp|P19804|N	RAT.fasta	17 283,3	100,0 %	4	7	8
sp|B2RYG6|O	RAT.fasta	31 270,5	100,0 %	2	2	3
sp|P27139|C/	RAT.fasta	29 114,3	100,0 %	7	8	10
sp|P01835|K/	RAT.fasta	11 600,7	100,0 %	8	12	27
sp|P31044|PI	RAT.fasta	20 801,4	100,0 %	17	21	32
sp|P49911|AI	RAT.fasta	28 565,3	100,0 %	2	2	3
sp|Q6IG03|K:	RAT.fasta	60 388,4	98,7 %	1	1	3
sp|P62815|V/	RAT.fasta	56 552,3	100,0 %	3	4	4
sp|P04764|EI	RAT.fasta	47 129,0	100,0 %	27	34	50
sp|Q62813-2|	RAT.fasta	37 324,0	100,0 %	4	6	7
tr|Q6P6G4|Q	RAT.fasta	30 077,3	100,0 %	3	3	4
sp|P62161|C/	RAT.fasta	16 838,0	100,0 %	3	5	8
sp|P62959|H	RAT.fasta	13 777,2	100,0 %	3	3	4
sp|P55053|F/	RAT.fasta	15 059,3	100,0 %	7	7	10
sp|P21575-3|	RAT.fasta	92 507,4	100,0 %	2	2	2
sp|Q8VI04|A!	RAT.fasta	34 410,7	100,0 %	4	4	11
tr|F1LSW0|F1LSW0_RAT		0,0	99,9 %	1	1	2
sp|P07632|S(	RAT.fasta	15 910,7	100,0 %	9	15	33
sp|P41498-2|	RAT.fasta	18 151,8	100,0 %	2	2	2
sp|O88600|H	RAT.fasta	94 057,4	100,0 %	4	4	6
sp|P68370|TI	RAT.fasta	50 135,7	100,0 %	2	3	3
tr|D3ZHM9|D	RAT.fasta	12 439,8	100,0 %	2	2	3
sp|Q62658|FI	RAT.fasta	11 922,7	100,0 %	3	3	5
sp|Q68FQ2|J.	RAT.fasta	34 782,6	100,0 %	2	2	3

sp|P01830|TI	RAT.fasta	18 172,8	100,0 %	7	9	11
sp|P82995|H!	RAT.fasta	84 818,3	100,0 %	11	13	18
sp|Q9R063|P	RAT.fasta	22 178,5	100,0 %	9	11	14
sp|P10960|S/	RAT.fasta	61 122,8	100,0 %	7	9	15
sp|P23562-2|	RAT.fasta	103 177,5	100,0 %	2	2	3
sp|P54690|B(	RAT.fasta	46 047,0	100,0 %	2	2	2
sp|P07151|B:	RAT.fasta	13 720,0	98,7 %	1	1	1
sp|P62329|T\	RAT.fasta	5 052,9	100,0 %	10	13	25
sp|P25304-2|	RAT.fasta	208 638,9	100,0 %	2	2	2
sp|Q9EQX9|L	RAT.fasta	17 124,6	100,0 %	5	5	7

et1

M (Oxidation), +42 on n (Acetyl), +80 on Y (Phospho)

Percentage of	Percentage sequence coverage
0,00265%	27,5%
0,00159%	11,6%
0,00106%	21,4%
0,000%	0,000%
0,00530%	9,73%
0,00318%	20,4%
0,000%	0,000%
0,00371%	4,86%
0,00530%	14,6%
0,000%	0,000%
0,000%	0,000%
0,000%	0,000%
0,00106%	9,38%
0,00106%	7,45%
0,000%	0,000%
0,000%	0,000%
0,00265%	19,0%
0,00371%	5,67%
0,00902%	5,29%
0,0414%	25,9%
0,00318%	5,88%
0,00106%	8,81%
0,000%	6,12%
0,00849%	27,9%
0,000530%	0,560%
0,000%	12,1%
0,00637%	30,0%
0,0541%	23,3%
0,000%	0,000%
0,00106%	8,43%
0,000%	0,000%
0,00424%	0,000%
0,000%	0,000%
0,000%	0,000%
0,000%	0,000%
0,00212%	19,1%
0,532%	88,5%
0,000%	0,000%
0,00106%	1,09%
0,000%	0,000%
0,000%	0,000%
0,000530%	3,39%
0,00265%	31,5%
0,00159%	3,97%

0,000%	0,000%
0,000%	0,000%
0,00955%	22,8%
0,000%	0,000%
0,00265%	0,000%
0,000%	0,000%
0,000%	0,000%
0,00159%	21,2%
0,00424%	26,1%
0,000%	0,000%
0,00530%	44,0%
0,00212%	9,30%
0,00265%	0,000%
0,000530%	6,95%
0,000%	0,000%
0,000%	4,23%
0,000%	0,000%
0,00106%	2,46%
0,00265%	18,8%
0,00106%	1,01%
0,0822%	60,1%
0,00106%	7,07%
0,000%	0,000%
0,00106%	7,09%
0,0361%	63,4%
0,0154%	54,1%
0,00265%	16,9%
0,00796%	6,02%
0,00371%	0,000%
0,000%	0,000%
0,00265%	18,3%
0,000%	0,000%
0,000%	0,000%
0,00106%	31,6%
0,000%	0,000%
0,000%	0,000%
0,000%	0,000%
0,000%	0,000%
0,00318%	17,6%
0,00530%	12,9%
0,00106%	9,89%
0,00106%	3,23%
0,000%	0,000%
0,00212%	12,9%
0,000%	0,000%
0,000530%	2,71%
0,00318%	1,83%
0,000%	0,000%
0,00106%	12,8%
0,000%	0,000%
0,00106%	2,42%

0,00212%	20,1%
0,000%	0,000%
0,00212%	3,83%
0,0557%	64,0%
0,00318%	5,84%
0,00690%	27,2%
0,000%	0,000%
0,000%	0,000%
0,0180%	54,1%
0,000%	0,000%
0,0276%	50,2%
0,000%	0,000%
0,00106%	6,86%
0,000%	0,000%
0,000%	0,000%
0,00106%	6,08%
0,00265%	3,60%
0,00318%	19,9%
0,000%	0,000%
0,000%	0,000%
0,528%	80,7%
0,00743%	29,0%
0,000%	0,000%
0,00265%	10,9%
0,000530%	1,78%
0,00159%	8,51%
0,00424%	9,51%
0,000%	0,000%
0,000%	0,000%
0,00106%	10,4%
0,00159%	5,56%
0,00212%	12,9%
0,000%	0,000%
0,00265%	9,53%
0,000%	0,000%
0,00796%	15,0%
0,000%	0,000%
0,00371%	6,85%
0,000530%	1,01%
0,00318%	25,5%
0,000%	0,000%
0,000%	0,000%
0,00477%	10,9%
0,00318%	23,2%
0,00212%	2,20%
0,000%	0,000%
0,0286%	33,9%
0,000%	0,000%
0,000%	0,000%
0,00584%	23,6%
0,0143%	16,5%

0,00424%	18,7%
0,000%	0,000%
0,00530%	46,7%
0,00637%	0,000%
0,00371%	3,89%
0,000%	0,000%
0,000%	0,000%
0,0233%	45,7%
0,000%	0,000%
0,000%	0,000%
0,000%	0,000%
0,179%	47,7%
0,000%	0,000%
0,000%	0,000%
0,00106%	0,637%
0,00849%	33,5%
0,000530%	14,0%
0,000%	0,000%
0,00106%	56,6%
0,00371%	0,000%
0,00106%	15,2%
0,00159%	11,3%
0,000%	0,000%
0,000%	0,000%
0,000530%	5,56%
0,000530%	6,67%
0,00477%	3,73%
0,000530%	2,08%
0,000%	0,000%
0,00637%	46,6%
0,00106%	9,73%
0,000530%	1,96%
0,000%	0,000%
0,00743%	15,5%
0,000530%	0,985%
0,00318%	13,0%
0,00477%	37,7%
0,00212%	19,5%
0,000%	7,09%
0,0138%	32,1%
0,0154%	41,5%
0,000%	0,000%
0,00212%	20,9%
0,000%	0,000%
0,000%	0,000%
0,000%	0,000%
0,00477%	16,6%
0,000%	0,000%
0,00902%	0,846%
0,0584%	27,1%
0,00106%	16,7%

0,00106%	0,000%
0,0143%	18,1%
0,0313%	52,1%
0,0228%	12,1%
0,00159%	6,36%
0,000%	0,000%
0,000%	0,000%
0,000%	0,000%
0,00159%	0,000%
0,00318%	10,4%
0,000%	0,000%
0,00265%	3,80%
0,000%	0,000%
0,00212%	3,70%
0,00318%	57,2%
0,000%	0,000%
0,000530%	7,37%
0,000%	0,000%
0,00212%	13,0%
0,000%	0,000%
0,00106%	3,68%
0,0127%	26,1%
0,000530%	0,733%
0,000%	0,000%
0,0122%	2,56%
0,00265%	17,5%
0,000%	0,000%
0,00212%	20,6%
0,00106%	4,61%
0,000%	0,000%
0,000530%	1,95%
0,00637%	6,12%
0,00371%	32,5%
0,000%	0,000%
0,000%	0,000%
0,000%	0,000%
0,000%	0,000%
0,0170%	4,93%
0,00477%	38,9%
0,00371%	42,6%
0,00477%	37,0%
0,00159%	4,86%
0,0260%	44,5%
0,000530%	1,51%
0,000530%	0,727%
0,000%	0,000%
0,00212%	21,0%
0,000%	0,000%
0,00212%	12,6%
0,00212%	15,6%
0,0111%	31,0%

0,00106%	8,35%
0,000%	0,000%
0,000%	0,000%
0,000%	0,000%
0,000%	0,000%
0,000%	0,000%
0,000%	0,000%
0,000%	0,000%
0,000530%	4,04%
0,000%	0,000%
0,00212%	0,000%
0,0366%	10,6%
0,000%	0,000%
0,000%	0,000%
0,0255%	32,4%
0,000%	0,000%
0,000530%	10,8%
0,00265%	20,6%
0,000%	0,000%
0,000%	0,000%
0,000%	0,000%
0,00318%	42,0%
0,000%	0,000%
0,000%	0,000%
0,00106%	21,5%
0,000%	0,000%
0,000%	0,000%
0,0175%	44,0%
0,00159%	19,8%
0,0472%	50,1%
0,00106%	1,70%
0,00265%	0,812%
0,000%	0,000%
0,000%	0,000%
0,000530%	0,000%
0,000%	0,000%
0,0265%	12,3%
0,00106%	4,33%
0,000%	0,000%
0,000%	0,000%
0,00637%	32,3%
0,0106%	20,9%
0,00106%	3,59%
0,00318%	23,8%
0,00106%	8,52%
0,0244%	36,1%
0,00424%	10,1%
0,00690%	13,8%
0,00265%	4,13%
0,000%	0,000%
0,000%	0,000%

0,241%	61,4%
0,0653%	38,4%
0,000%	0,000%
0,000%	0,000%
0,000%	0,000%
0,00106%	0,000%
0,00796%	26,1%
0,00159%	12,6%
0,00106%	3,72%
0,00212%	21,6%
0,00106%	5,67%
0,000%	0,000%
0,000%	0,000%
0,000530%	1,70%
0,00159%	68,4%
0,0971%	31,2%
0,000%	0,000%
0,000%	0,000%
0,000530%	2,95%
0,000%	0,000%
0,00212%	13,3%
0,00159%	10,5%
0,000%	0,000%
0,000%	0,000%
0,00265%	11,9%
0,00106%	2,37%
0,00637%	26,6%
0,00106%	0,000%
0,0308%	0,000%
0,000530%	8,70%
0,000530%	1,97%
0,00690%	30,4%
0,000%	0,000%
0,00159%	11,5%
0,000530%	1,98%
0,00106%	10,1%
0,000%	0,000%
0,000530%	1,55%
0,000%	0,000%
0,00106%	6,26%
0,000%	0,000%
0,00212%	11,4%
0,000%	0,000%
0,00159%	8,09%
0,0647%	9,76%
0,000%	0,000%
0,00265%	6,97%
0,000%	3,55%
0,00106%	0,000%
0,00477%	30,5%
0,00955%	65,7%

0,00265%	19,0%
0,000%	0,000%
0,000%	0,000%
0,00796%	17,3%
0,00318%	4,58%
0,00159%	8,78%
0,000%	0,000%
0,00159%	0,000%
0,000%	0,000%
0,0133%	41,4%
0,00477%	17,4%
0,00796%	56,3%
0,000%	0,000%
0,00212%	11,8%
0,0122%	48,6%
0,00584%	7,70%
0,00637%	0,000%
0,000%	0,000%
0,000%	0,000%
0,000%	0,000%
0,000%	0,000%
0,00159%	4,20%
0,0493%	0,000%
0,000%	0,000%
0,00477%	4,52%
0,000%	0,000%
0,00106%	0,000%
0,00106%	7,73%
0,000%	0,000%
0,000%	0,000%
0,000530%	0,000%
0,00796%	23,1%
0,000%	0,000%
0,000%	0,000%
0,000%	0,000%
0,00318%	4,85%
0,00318%	33,6%
0,000530%	0,592%
0,000530%	6,10%
0,00212%	35,7%
0,0106%	15,9%
0,000%	0,000%
0,000530%	3,68%
0,00159%	9,89%
0,000%	0,000%
0,0218%	70,5%
0,000%	0,000%
0,000530%	2,46%
0,00159%	2,69%
0,00106%	11,3%
0,00159%	33,9%

0,00159%	14,8%
0,0117%	6,18%
0,000%	0,000%
0,000530%	2,29%
0,000%	0,000%
0,105%	44,2%
0,00637%	13,8%
0,0271%	70,1%
0,00106%	0,614%
0,000%	0,000%
0,000530%	2,74%
0,0196%	10,8%
0,000%	0,000%
0,00106%	1,36%
0,00106%	5,96%
0,00159%	11,0%
0,00371%	13,8%
0,127%	65,9%
0,00477%	37,8%
0,00265%	28,6%
0,00106%	8,61%
0,000530%	7,80%
0,000%	0,000%
0,00212%	86,7%
0,00212%	27,1%
0,000530%	0,765%
0,000%	9,22%
0,000%	0,000%
0,000%	0,000%
0,000%	4,25%
0,000%	0,000%
0,000%	0,000%
0,00611%	7,65%
0,000%	0,000%
0,00917%	8,91%
0,00611%	3,40%
0,00611%	0,000%
0,000%	0,000%
0,00611%	4,26%
0,00306%	6,31%
0,00611%	0,000%
0,00306%	2,43%
0,0122%	53,9%
0,00611%	3,06%
0,00306%	7,24%
0,00306%	2,09%
0,00611%	0,000%
0,00611%	4,88%
0,000%	0,000%
0,000%	0,000%
0,00917%	5,73%

0,00306%	5,51%
0,00306%	2,33%
0,000%	0,000%
0,00917%	6,90%
0,000%	0,000%
0,00917%	1,83%
0,000%	0,000%
0,00917%	25,8%
0,00611%	0,812%
0,00611%	10,4%
0,00611%	5,58%
0,00611%	1,51%
0,00611%	14,0%
0,0275%	27,6%
0,00611%	4,73%
0,0153%	16,2%
0,0795%	17,7%
0,000%	0,000%
0,00917%	1,48%
0,177%	60,1%
0,00917%	9,16%
0,0122%	11,3%
0,0122%	23,3%
0,00306%	10,6%
0,0245%	50,7%
0,00611%	9,80%
0,410%	34,3%
0,00306%	1,13%
0,000%	0,000%
0,000%	0,000%
0,000%	0,000%
0,00306%	2,48%
0,00306%	7,89%
0,0153%	8,09%
0,000%	0,000%
0,00917%	8,08%
0,00306%	1,96%
0,00917%	8,68%
0,0306%	30,4%
0,00611%	14,1%
0,00306%	10,5%
0,0275%	6,51%
0,000%	1,78%
0,000%	0,000%
0,0673%	60,5%
0,0428%	9,36%
0,00306%	5,53%
0,000%	0,000%
0,00611%	0,000%
0,00917%	19,6%
0,0734%	37,2%

0,0336%	17,2%
0,000%	0,000%
0,0122%	23,8%
0,0153%	10,2%
0,0183%	49,6%
0,0275%	33,0%
0,0520%	2,54%
0,000%	0,000%
0,0367%	19,6%
0,00306%	2,07%
0,000%	0,000%
0,00917%	2,41%
0,104%	45,3%
0,00917%	32,9%
0,0397%	26,7%
0,0122%	19,3%
0,0183%	23,4%
0,00611%	8,85%
0,00917%	0,000%
0,0153%	11,7%
0,000%	0,000%
0,0153%	22,9%
0,0948%	0,000%
0,113%	14,4%
0,00306%	5,51%
0,00917%	21,5%
0,00306%	6,43%
0,00306%	1,09%
0,000%	8,12%
0,0183%	0,000%
0,00306%	14,4%
0,000%	0,000%
0,0122%	15,8%
0,00611%	14,4%
0,0367%	29,5%
0,0122%	7,38%
0,00611%	0,000%
0,0153%	5,35%
0,0642%	36,1%
0,0428%	16,4%
0,0122%	25,3%
0,0459%	12,2%
0,00917%	10,1%
0,00306%	0,733%
0,000%	0,000%
0,0183%	10,3%
0,00306%	5,25%
0,0122%	2,74%
1,39%	77,7%
0,0153%	0,000%
0,0367%	51,7%

0,0183%	22,5%
0,0183%	3,62%
0,0367%	19,9%
0,000%	0,404%
0,00917%	7,42%
0,000%	0,000%
0,00306%	1,62%
0,00611%	27,6%
0,0122%	10,7%
0,810%	56,3%
0,0122%	6,33%
0,0336%	11,1%
0,0795%	12,2%
0,00611%	7,23%
0,0887%	46,6%
0,0306%	42,1%
0,00306%	4,62%
0,00611%	19,4%
0,000%	0,000%
0,327%	59,3%
0,00306%	77,0%
0,0214%	8,13%
0,0183%	10,9%
0,0397%	60,8%
0,0122%	12,8%
0,000%	0,000%
0,0306%	7,82%
0,00306%	7,83%
0,0489%	59,9%
0,000%	0,000%
0,00611%	0,000%
0,0153%	12,8%
0,0153%	13,0%
0,00306%	6,95%
0,00306%	4,81%
0,00306%	11,1%
0,128%	57,5%
0,00917%	9,73%
0,000%	0,000%
0,000%	0,000%
0,0275%	14,1%
0,000%	0,000%
0,000%	5,97%
0,0367%	35,7%
0,0245%	0,000%
0,0245%	7,96%
0,00611%	9,41%
0,0825%	21,2%
0,00917%	9,95%
0,00306%	3,28%
0,0183%	11,1%

0,00917%	20,4%
0,00306%	5,51%
0,00611%	0,000%
0,0581%	24,1%
0,0306%	15,6%
0,00306%	0,610%
0,000%	0,000%
0,0245%	37,9%
0,00917%	0,951%
0,0306%	11,5%
0,000%	0,846%
0,0734%	0,000%
0,000%	0,000%
0,00611%	20,4%
0,107%	48,5%
0,0917%	16,5%
0,00306%	8,70%
0,00917%	7,12%
0,00306%	6,48%
0,00611%	0,000%
0,00917%	19,0%
0,00611%	18,8%
0,202%	32,5%
0,0153%	86,4%
0,00917%	2,21%
0,00611%	12,0%
0,00917%	10,4%
0,00917%	4,55%
0,0336%	34,0%
0,000%	0,000%
0,000%	0,000%
0,000%	0,000%
0,0795%	0,000%
0,00917%	31,6%
0,0367%	0,000%
0,00306%	6,80%
0,00611%	3,64%
0,150%	22,4%
0,000%	0,000%
0,0122%	4,08%
0,00611%	6,40%
0,00611%	3,47%
0,00306%	2,02%
0,00306%	0,000%
0,000%	0,000%
0,495%	45,7%
0,00611%	0,000%
0,0489%	22,3%
0,00306%	13,0%
0,104%	28,5%
0,144%	20,5%

1,06%	82,1%
0,00306%	5,67%
0,000%	0,000%
0,00611%	25,6%
0,00611%	8,39%
0,00306%	1,57%
0,00306%	3,00%
0,00306%	5,71%
0,107%	47,7%
0,00306%	3,33%
0,00917%	9,59%
0,00306%	2,01%
0,0122%	30,9%
0,000%	0,000%
0,000%	0,000%
0,00917%	1,95%
0,0153%	10,4%
0,00306%	3,36%
0,0183%	28,0%
0,0978%	9,12%
0,0183%	9,17%
0,00306%	6,57%
0,00917%	0,000%
0,0795%	23,4%
0,00611%	1,49%
0,00611%	0,000%
0,0214%	11,8%
0,000%	0,000%
0,0306%	3,48%
0,0214%	28,5%
0,00306%	2,25%
0,000%	2,11%
0,0489%	27,0%
0,0275%	14,6%
0,00306%	4,08%
0,0336%	9,97%
0,0122%	10,6%
0,00917%	0,000%
0,0275%	22,6%
0,00306%	1,06%
0,00306%	3,59%
0,000%	0,000%
0,0764%	49,8%
0,00917%	11,8%
0,0153%	3,45%
0,00306%	6,24%
0,00611%	21,4%
0,00917%	25,3%
0,0428%	38,5%
0,0122%	0,000%
0,0214%	30,7%

0,370%	40,4%
0,116%	58,1%
0,00611%	9,43%
0,0245%	6,47%
0,00611%	2,05%
0,0489%	9,78%
0,00611%	20,5%
0,0122%	4,76%
0,00611%	19,2%
0,00306%	2,90%
0,00550%	4,98%
0,00367%	0,000%
0,0642%	54,0%
0,0220%	7,46%
0,00734%	8,80%
0,00550%	17,8%
0,0239%	15,2%
0,00550%	51,6%
0,00367%	1,06%
0,0550%	51,6%
0,00367%	6,57%
0,0367%	54,0%
0,0789%	63,5%
0,00367%	0,000%
0,0110%	21,6%
0,00734%	13,6%
0,00550%	6,28%
0,00183%	0,675%
0,0110%	24,2%
0,0147%	15,2%
0,00550%	18,1%
0,0128%	4,95%
0,0459%	45,3%
0,0459%	35,7%
0,134%	59,6%
0,000%	2,21%
0,00550%	9,06%
0,00917%	8,39%
0,00367%	9,31%
0,117%	43,0%
0,0110%	11,2%
0,000%	2,31%
0,00550%	4,27%
0,00734%	10,8%
0,130%	32,1%
0,0202%	25,6%
0,0183%	25,7%
0,00550%	29,8%
0,00367%	15,6%
0,158%	43,3%
0,0569%	6,65%

0,00550%	1,43%
0,0239%	16,7%
0,0495%	36,2%
0,0165%	26,0%
0,00367%	4,27%
0,0110%	11,8%
0,00550%	14,0%
0,0165%	0,000%
0,158%	49,4%
0,0440%	37,5%
0,0128%	41,9%
0,0165%	33,0%
0,0128%	13,0%
0,0716%	45,4%
0,0716%	62,7%
0,0862%	83,4%
0,00367%	15,2%
0,0110%	6,10%
0,00367%	22,8%
0,0275%	53,1%
0,00183%	8,61%
0,0550%	40,3%
0,174%	90,1%
0,00917%	32,5%
0,0917%	36,0%
0,0128%	24,9%
0,00550%	8,52%
0,0899%	24,3%
0,00734%	12,5%
0,0661%	53,9%
0,0275%	62,4%
0,0459%	47,1%
0,308%	64,3%
0,0128%	9,11%
0,0239%	34,0%
0,00367%	13,1%
0,000%	0,000%
0,0128%	7,75%
0,00550%	2,97%
0,0110%	29,0%
0,0257%	73,6%
0,00367%	9,78%
0,0422%	16,8%
0,00734%	49,5%
0,00917%	2,57%
0,0128%	10,2%
0,00734%	22,3%
0,00550%	11,6%
0,00550%	8,51%
0,0220%	20,0%
0,00367%	3,99%

0,0110%	17,5%
0,0275%	34,0%
0,00367%	5,97%
0,0917%	83,9%
0,0275%	14,5%
0,000%	0,000%
0,00367%	0,000%
0,0110%	51,1%
0,0385%	0,000%
0,0165%	0,000%
0,0734%	32,5%
0,0202%	29,4%
0,00367%	0,000%
0,0128%	21,7%
0,00367%	6,63%
0,00550%	10,5%
0,0385%	28,2%
0,0110%	14,3%
0,00734%	4,35%
0,000%	0,000%
0,0183%	24,9%
0,0183%	12,3%
0,00734%	5,26%
0,00367%	5,37%
0,00367%	12,8%
0,00734%	5,51%
0,0367%	75,8%
0,0440%	0,000%
0,0183%	18,3%
0,00917%	16,5%
0,0128%	5,09%
0,00183%	2,46%
0,0128%	43,3%
0,0110%	3,38%
0,112%	0,000%
0,0514%	72,6%
0,0679%	43,3%
0,000%	0,000%
0,0110%	16,3%
0,0165%	36,5%
0,0789%	39,8%
0,141%	45,6%
0,00183%	8,09%
0,00367%	6,97%
0,00367%	9,31%
0,0844%	63,2%
0,0110%	20,4%
0,0110%	27,4%
0,00550%	9,72%
0,00917%	16,0%
0,00917%	19,7%

0,00917%	38,4%
0,0128%	5,38%
0,000%	1,86%
0,0165%	0,000%
0,00734%	15,5%
0,00734%	13,9%
0,0404%	13,2%
0,00183%	4,62%
0,00367%	1,06%
0,00550%	31,3%
0,00917%	0,000%
0,0128%	24,6%
0,00917%	34,3%
0,0349%	72,9%
0,00917%	16,0%
0,000%	0,000%
0,0165%	21,4%
0,114%	63,5%
0,00550%	20,5%
0,0239%	37,6%
0,0165%	23,7%
0,00734%	5,03%
0,000%	0,000%
0,00550%	5,83%
0,00367%	7,30%
0,000%	0,000%
0,706%	76,2%
0,0165%	27,5%
0,0477%	51,2%
0,00550%	39,0%
0,0550%	14,8%
0,0128%	17,0%
0,00550%	4,92%
0,0128%	39,0%
0,00367%	6,99%
0,0477%	58,3%
0,00734%	29,9%
0,0147%	15,2%
0,0183%	18,0%
0,00367%	63,8%
0,00367%	6,94%
0,0972%	64,0%
0,0128%	12,5%
0,00917%	10,3%
0,000%	0,000%
0,0183%	18,8%
0,0183%	34,1%
0,0239%	28,7%
0,0367%	22,8%
0,00734%	0,000%
0,00917%	14,0%

0,0330%	41,0%
0,0550%	74,4%
0,0110%	22,4%
0,00734%	26,7%
0,00550%	2,49%
0,00367%	4,56%
0,00183%	2,08%
0,00183%	1,42%
0,0275%	0,000%
0,0110%	17,5%
0,0349%	20,5%
0,0514%	96,6%
0,00550%	7,09%
0,0183%	25,7%
0,0257%	41,0%
0,000%	0,000%
0,0202%	30,2%
0,0385%	37,4%
0,00367%	6,65%
0,0477%	48,2%
0,000%	0,000%
0,000%	0,000%
0,000%	0,000%
0,00367%	2,08%
0,00734%	5,76%
0,0147%	11,1%
0,00367%	11,0%
0,0147%	20,6%
0,0679%	32,2%
0,00734%	11,0%
0,0128%	0,000%
0,0275%	29,0%
0,00734%	10,0%
0,0257%	43,4%
0,000%	0,000%
0,00550%	8,68%
0,00550%	6,41%
0,00183%	1,97%
0,0220%	18,5%
0,00367%	47,1%
0,0110%	19,7%
0,0183%	20,1%
0,00917%	16,4%
0,0257%	39,9%
0,00550%	7,74%
0,0183%	45,7%
0,00550%	6,32%
0,00183%	15,3%
0,00550%	12,0%
0,0147%	40,7%
0,0404%	23,7%

0,0147%	35,7%
0,00183%	1,84%
0,00917%	14,5%
0,0514%	48,2%
0,00367%	1,69%
0,0165%	11,9%
0,00550%	2,04%
0,00550%	8,64%
0,0294%	35,9%
0,0605%	18,6%
0,00367%	5,83%
0,0183%	24,6%
0,0422%	23,0%
0,00183%	7,17%
0,0972%	17,4%
0,000%	0,000%
0,00550%	19,7%
0,000%	0,000%
0,0294%	8,24%
0,0165%	0,000%
0,00917%	7,66%
0,0550%	58,6%
0,0789%	0,000%
0,0183%	4,79%
0,0110%	35,7%
0,0239%	11,6%
0,00734%	34,7%
0,000%	0,000%
0,0128%	10,8%
0,0495%	26,0%
0,0239%	18,8%
0,00367%	32,1%
0,00367%	27,3%
0,0165%	30,2%
0,00367%	8,66%
0,00367%	8,72%
0,00367%	0,000%
0,0110%	16,4%
0,00183%	12,8%
0,268%	96,6%
0,00367%	27,6%
0,00734%	3,02%
0,00734%	11,0%
0,000%	0,000%
0,00367%	6,83%
0,00917%	8,78%
0,0275%	21,9%
0,0605%	28,2%
0,0128%	51,3%
0,00734%	20,4%
0,0330%	55,1%

0,00550%	29,6%
0,00550%	13,9%
0,0716%	55,2%
0,00367%	8,82%
0,00917%	30,1%
0,0128%	31,3%
0,0147%	28,6%
0,0110%	3,37%
0,00550%	21,8%
0,0220%	76,9%
0,00734%	4,01%
0,0110%	14,8%
0,00734%	6,74%
0,0367%	31,7%
0,00734%	49,4%
0,00917%	16,0%
0,0128%	39,2%
0,00734%	15,7%
0,0294%	35,0%
0,0147%	7,48%
0,000%	0,000%
0,00734%	23,3%
0,0183%	65,0%
0,00734%	39,4%
0,00550%	19,4%
0,0128%	10,7%
0,00550%	5,78%
0,0147%	24,3%
0,00917%	23,2%
0,000%	0,000%
0,00367%	9,22%
0,00734%	14,7%
0,00917%	11,7%
0,00550%	15,8%
0,00550%	4,44%
0,00367%	2,49%
0,0514%	34,3%
0,000%	0,000%
0,00367%	1,71%
0,00367%	15,1%
0,0954%	71,7%
0,00550%	10,9%
0,0404%	73,0%
0,0128%	18,8%
0,0954%	55,3%
0,00917%	33,6%
0,00367%	15,9%
0,0147%	24,8%
0,00917%	28,6%
0,0349%	54,3%
0,0165%	6,21%

0,178%	53,5%
0,00550%	18,1%
0,0183%	68,0%
0,0367%	6,88%
0,00734%	12,9%
0,00550%	3,82%
0,00550%	34,8%
0,00367%	2,68%
0,0110%	8,13%
0,0275%	25,1%
0,0257%	15,4%
0,0771%	66,0%
0,00917%	27,0%
0,0220%	48,7%
0,0679%	29,6%
0,00367%	42,1%
0,00917%	3,80%
0,0239%	50,8%
0,00917%	51,1%
0,00917%	12,4%
0,00550%	9,46%
0,0183%	16,6%
0,0128%	17,5%
0,0349%	8,62%
0,00917%	27,2%
0,00367%	27,9%
0,0220%	70,3%
0,0147%	68,4%
0,00550%	11,4%
0,0183%	46,5%
0,0495%	81,1%
0,0587%	88,8%
0,00550%	17,4%
0,00550%	2,17%
0,00734%	9,59%
0,0917%	64,3%
0,0128%	18,6%
0,00734%	20,5%
0,0147%	30,9%
0,00734%	32,5%
0,0183%	44,4%
0,00367%	4,42%
0,0202%	19,8%
0,00367%	0,000%
0,0605%	84,4%
0,00367%	13,3%
0,0110%	6,79%
0,00550%	62,7%
0,00550%	13,0%
0,00917%	50,9%
0,00550%	10,00%

0,0202%	26,1%
0,0330%	31,1%
0,0257%	49,3%
0,0275%	14,4%
0,00550%	3,66%
0,00367%	7,54%
0,00183%	7,56%
0,0459%	93,2%
0,00367%	1,59%
0,0128%	40,1%
